# Owned House Cats Show No Preference for Specific Land Cover Types When Roaming Outdoors

**DOI:** 10.3390/ani16060864

**Published:** 2026-03-10

**Authors:** Lyan Wolovelsky, Noy Kadosh, Moshe Gish

**Affiliations:** School of Environmental Sciences, Faculty of Social Sciences, University of Haifa, Abba Khoushy Ave. 199, Haifa 3103301, Israel

**Keywords:** cat roaming, domestic cats, GPS tracking, habitat selection, home range, resource availability, resource selection

## Abstract

Owned house cats negatively affect wildlife when roaming outdoors, yet it remains unclear whether their movements are determined by selection for specific land cover types. We GPS-tracked 49 neutered, owned cats with daily outdoor access in a Mediterranean habitat, monitoring each for seven days. For each cat, we created a detailed, fine-scale land cover map from near-contemporaneous aerial imagery. We delineated each cat’s home range and compared its land cover composition to that of an equal-area circle centered on the median x and y coordinates of GPS fixes, representing directionally unbiased (isotropic) roaming. Home ranges were small (mean 0.85 ± 0.06 ha; median 0.73 ha; approximately 50 m radius), and cats whose ranges included nearby natural or semi-natural open areas had larger home ranges than cats in urban environments. Home range size was not affected by sex or season and decreased with age. Despite detailed, accurate mapping, no land cover class was found to be over- or under-represented within cat home ranges relative to the null expectation. These results, although limited to harness-tolerant cats, suggest that owned cats in our study area do not preferentially select specific land covers when roaming outdoors.

## Simple Summary

Pet cats that are allowed outdoors are known to hunt wildlife, but it remains unclear whether they prefer to roam in areas that offer more hunting opportunities. In this study, we fitted pet cats with GPS harnesses to track their movements around their homes. Using detailed, up-to-date land cover maps, we found that cats usually stayed very close to home and ranged only moderately farther when natural open areas were nearby. However, cats showed no preference for any particular habitat or land cover type. These findings suggest that the roaming behavior of pet cats is not primarily driven by a search for hunting grounds, but rather reflects general exploration of their environment. Therefore, buffer zones around human settlements may not need to be extensive to reduce the impact of pet cats on natural habitats, since cats are not expected to preferentially venture into these areas.

## 1. Introduction

House cats (*Felis catus*) are among the most widespread companion animals globally, occupying a unique socio-ecological niche as both household members and free-ranging predators [1,2,3]. Behavioral studies reveal that cats living in human households retain their hunting instincts when outdoors, despite being regularly fed by their owners [4,5,6]. Given their vast numbers, cats are believed to contribute significantly to wildlife decline within and around human settlements [1,2,3,7]. This impact is particularly pronounced in locations where residential neighborhoods border remnant native vegetation, protected areas, or other habitats of conservation importance. Several studies of free-ranging cats, including both owned and unowned individuals, have shown that they roam into protected areas and may occasionally travel more than 500 m from houses into rural and natural environments [8,9,10,11]. As a result, outdoor-roaming cats, both owned and unowned, have become a major concern in debates on the conservation of biodiversity within and around human settlements [3,12,13,14].

Recognizing that cats are ubiquitous in human-inhabited areas, management strategies generally focus on compromise rather than outright prohibition, promoting measures such as encouraging owners to provide high-meat-protein food and object play, implementing nighttime curfews, establishing exclusion zones near sensitive habitats, and maintaining buffer distances [1,2,3,4,5,10,12,15]. For instance, Palomares & Sanglas [10] recommend buffer zones of at least 2.5 km around conservation sites. The success of such interventions, however, depends on a thorough understanding of cat movement patterns and habitat use, which has been studied in various settings for pet cats [2,6,16,17,18].

Cats that roam outdoors, whether owned or unowned, affect wildlife not only through predation or injury, but also through indirect mechanisms [3,7,12,13,19]; even in the absence of frequent kills, their presence can alter prey behavior, increase vigilance and stress, reduce foraging efficiency, and displace native species from preferred habitats [5,12]. These impacts justify precaution even when population-level consequences are uncertain, particularly in habitats that support threatened or declining species [8,9,14,19,20].

The concept of a cat’s home range (the spatial area regularly used for exploration, foraging, and seeking mates) is central to understanding the ecological influence of roaming cats. While large home ranges increase the influence range of cats, small home ranges, as reported in many studies, e.g., [6,18,19,21], (see also Table 1), concentrate a cat’s predation pressure in a smaller area. For the purpose of biodiversity protection in natural habitats, home range size is often used as a simple proxy for the potential ecological footprint of cats, and it informs management practices such as determining buffer zone size [10,15]. Early radio telemetry studies reported cat home ranges spanning from less than 1 ha to around 30 ha [22,23]. More recent studies that performed GPS tracking of owned cats have found median home ranges of approximately one to two hectares in urban areas, with larger ranges, often reaching tens of hectares, observed in rural or natural environments [2,10,17]. Studies on free-ranging or unowned cats in protected or semi-natural areas often reveal even greater spatial use [8,9,10].

Considerable individual variation in home range size has also been documented, even among cats living in the same neighborhood [1,2,6,18]. Both intrinsic and extrinsic factors influence home range size. Intrinsic drivers include age, sex, and reproductive status: some studies have found that males or intact individuals roam more widely, while others have observed only weak or context-dependent patterns [18,28,29,30]. Extrinsic factors include cat density and resource availability, including owner-supplied food [4,15,22,27].

Home range size alone does not fully capture how cats utilize space. For effective conservation and management, it is often more informative to understand habitat selection, including whether cats use certain land cover types more than their availability would suggest. Research on owned cats demonstrates considerable variation in selection patterns. Some individuals spend most of their time in vegetated green spaces or habitat edges [17,23,27], while others remain close to buildings and modified environments [2,6,18,22]. Unowned cats, on the other hand, are often found to use natural habitats extensively, possibly drawn away from buildings by higher resource availability [8,9,10,20].

Various mechanisms may influence cats’ attraction to or avoidance of specific land cover types. Areas that offer increased prey encounters, such as gardens, natural habitat edges, or mixed mosaics, are thought to be preferred by cats [15,27,31]. Structurally complex habitats may enhance hunting success by providing cover and reducing detection [15,17,23]. Roads and high-traffic areas may be avoided because of collision risk or their function as barriers, though some cats may use linear features for movement [2,16,17,22,32]. In regions with larger predators or high cat density, cats may reduce their use of exposed green spaces, presumably to avoid conflict [10,32].

Identifying specific land covers that are disproportionately used by cats may help guide management efforts at a fine scale. When cats use natural fragments, riparian corridors, or protected areas, these locations could become priority zones for mitigation, such as buffer zones, curfews, or specific design interventions [8,9,10,15,17,20]. If cats mainly remain within gardens and built environments, management could instead focus on interventions like confining cats during critical periods, modifying gardens and fences, or collar-mounted deterrents [1,2,3,17]. Identifying which land cover types are most strongly selected by which cats, and under what conditions, is therefore essential for effective, context-specific management.

Although many studies have quantified home range size and habitat use of owned cats, land cover within those ranges is often characterized too coarsely for the fine spatial scale at which owned cats actually operate. Large-scale syntheses and multi-site studies frequently rely on proxy measures of “urbanization” (e.g., human population density) and simplify environments into broad habitat classes such as “disturbed” versus “natural”, derived from generalized land cover layers [2,18]. Even within single-region studies, urbanization is typically described using settlement-level metrics (e.g., households per hectare or the proportion of constructed or impervious surfaces) rather than fine-grained land cover composition surrounding each tracked individual [25]. Likewise, habitat selection analyses often collapse space into a small number of categories, such as green-space and urban [1,33] or impervious, roads, green-space, and agriculture, usually based on low-resolution, high-error land cover maps [17]. In some cases, habitat preference is inferred without accounting for habitat availability [18]. Although coarse, low-resolution land cover classification can facilitate comparisons of home range size across environments, it offers limited insight into actual cat preferences, which require accurate, detailed, up-to-date, high-resolution land cover classification within each cat’s home range.

Some studies have mapped habitats within individual ranges using aerial imagery [16,34], but even these typically employ relatively low resolutions and broad thematic categories, which can obscure the fine-scale heterogeneity that may be important within very small home ranges. This limitation is especially relevant because, despite indications from a few older studies, recent research has shown that owned cats often remain close to home, with median maximum distances of travel from the house on the order of 100 m [1,25], and most outdoor time occurring within 50 m of the house [6,18]. However, even the highest quality land cover maps cannot resolve another significant issue: at the small spatial scale of an owned cat’s range, highly distinct features such as gardens, hedges, driveways, alleys, and small patches of tree cover are likely to blend together due to measurement noise. GPS errors from commonly used tracking devices can be on the order of tens of meters depending on environmental conditions [1,25,30], which can shift fixes across adjacent polygons and significantly inflate noise.

Therefore, the goal of this study was to investigate land cover preferences of GPS-tracked owned cats using a method that is less sensitive to these limitations. Using high-resolution, contemporaneous aerial imagery to build per-cat, detailed land cover maps within each individual cat’s roaming area, we tested whether the shape and placement of each cat’s home range were influenced by the fine-scale composition of land covers present within the home range.

## 2. Materials and Methods

### 2.1. Study Area and Volunteer Recruitment

The study was conducted in the Mediterranean region of Israel (northern and central Israel) over a one-year period (March 2019–February 2020). Volunteer cat owners were recruited through word-of-mouth and social-media advertisements. Inclusion criteria were: (i) owned cats allowed daily outdoor access, (ii) adult cats aged 1–12 years, (iii) neutered, and (iv) body mass ≥3 kg. A total of 49 cats (23 females, 26 males) met these criteria and completed the full tracking protocol. Mean age was 4.8 ± 0.5 years (SE); median = 4. Individual cat characteristics and spatial data are provided in Supplementary Table S1. Owners received written instructions on fitting the GPS harness and monitoring cat welfare, and additional guidance was provided by phone or in person as needed. Owners were instructed to keep their cats indoors under supervision for several hours after fitting the harness and to terminate participation immediately if signs of distress or substantial behavioral change occurred.

### 2.2. Tracking Devices

We tracked cats using I-gotU 600 GPS data loggers (Mobile Action Technology, Taipei, Taiwan). Each unit was housed in a 3D-printed thermoplastic polyurethane (TPU) case and mounted on an adjustable cat harness designed to position the device on the cat’s back rather than hanging from the neck (which may be less comfortable for the cat and may have worse satellite reception in built-up areas), similar to setups used in previous cat GPS-tracking studies [20,33,35]. The combined mass of the logger, casing, and harness was 65 g, which is consistent with conservative guidelines for cat tracking devices [36].

### 2.3. GPS Data Collection

Owners completed a brief questionnaire about their cat and fitted the harness once it had been fully charged. The harness remained on the cat for eight consecutive days, with no need for additional charging. Owners were asked to keep their cats indoors for several hours after fitting the harness, to allow acclimation and to ensure cats were not distressed [37]. Because most cats (30 of 49) did not routinely wear a collar or harness, the first day was treated as an acclimation day, and its data were excluded from analyses to reduce potential acclimation effects [24]. The GPS units were configured to record locations every three minutes with motion detection enabled [18].

Season was classified as follows: Since autumn and spring are very short in the study area (the Mediterranean region of Israel, where winters normally last from December to February, and autumn and spring typically last roughly one and a half months and are characterized by extremely variable weather), we chose a dichotomous classification of seasons: “winter” or “summer”. A tracking session was assigned to “winter” if it occurred between December and February, included at least one rainy day, or if the mean maximum daytime temperature during the session did not exceed 20 °C. All other sessions were classified as “summer”.

Cats that showed signs of discomfort or distress had their harness removed and their participation in the experiment was terminated (nine cats were excluded from this study for this reason).

### 2.4. GPS Sample Cleaning and Delineation of Roaming Area

The accuracy of GPS trackers is impaired when the device’s direct view of the sky is obstructed. For instance, when a cat is indoors or under dense vegetation, GPS accuracy is often reduced. Additional inaccuracies may occur when the tracker is first initialized or when it reconnects after losing satellite reception.

To clean the data and remove erroneously logged points (noise), we excluded the 5% of fixes farthest from the center, retaining the central 95% (95% MCP; Supplementary Figure S1). This was accomplished using a One-class Support Vector Machine (OC-SVM) with a Radial Basis Function (RBF) kernel, as implemented in scikit-learn version 1.4.0, running in Python 3.12 (Python Software Foundation, Beaverton, OR, USA) [38]. The nu parameter of the OC-SVM was set to 0.05 (the fraction of data considered as outliers). For the RBF kernel, we used a kernel’s bandwidth of γ = 0.1. After training the OC-SVM, we were able to separate fixes into outliers and inliers. The Area of Interest (AOI) was then determined as the outer perimeter of the polygon containing all inlier fixes.

The γ parameter of the RBF kernel in a one-class SVM controls the effective bandwidth of the decision boundary: larger γ yields a more complex and irregular boundary that closely follows the data, whereas smaller γ produces a smoother boundary. In our implementation, we set γ = 0.1, which is lower than the common heuristic of 1 divided by the number of features. Here, the number of features refers to the number of dimensions per observation; in our case, each GPS fix is a 2-D point (latitude and longitude), so there are two features. This heuristic and its default settings are widely used in the machine-learning community and documented in scikit-learn, a standard open-source library [39,40]. We chose a smaller γ value to favor a smoother boundary and reduce sensitivity to GPS noise. In practice, however, the exact γ value has limited impact on the final outcome, because the home range is ultimately estimated via a convex hull (MCP) over the selected inlier points, which makes the results robust to moderate variations in γ.

For the purposes of this research, noise that falls within the cat’s home range is immaterial, as we examined the size of the AOI and the composition of land covers it contains, rather than the time spent in specific areas or the exact locations of individual GPS fixes within it.

### 2.5. Aerial Photographs: Collection, Processing, and Land Cover Labeling

#### 2.5.1. Imagery Source and Timing

High-resolution land cover-labeled maps were not available for the study region at the spatial scale required for owned cats’ small home ranges. We therefore manually created custom land cover maps based on high-resolution aerial photographs (resolution: 0.6 m per pixel) obtained from Israel’s governmental mapping portal (www.govmap.gov.il). Because cat movement data were collected between March 2019 and February 2020, and the aerial photographs were taken at the beginning of 2020, the imagery may be considered near-contemporaneous with the tracking data.

#### 2.5.2. Automated Image Processing and Polygonization

**Figure 1:**
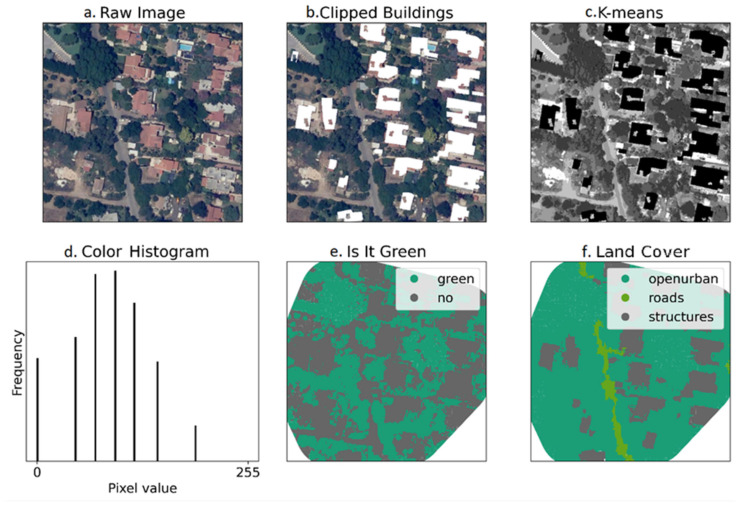
Workflow for deriving polygon land cover layers from aerial imagery for each cat’s roaming area. (**a**) Raw aerial photograph. (**b**) Removal of buildings. (**c**) Classifying the images by color values using K-means. (**d**) Color histogram for seven shades of gray. (**e**) Separation of sections of vegetation from all other areas, clipped to the cat’s area of interest (AOI). (**f**) Final land cover layer after manual polygon labeling in QGIS using the six-class land cover scheme (Table 2).

For each cat, we processed aerial imagery covering the AOI using a Python workflow designed to produce a polygon layer suitable for manual land cover labeling. The workflow consisted of four steps:

1. Removal of buildings from the raw image (Figure 1a): This step was necessary because roof colors often blend with the colors of nearby soil, unpaved roads, or parking lots, which could cause several land covers to merge into a single polygon during later processing. Although it is possible to analyze aerial images without first removing the buildings, this would require more manual correction at later stages to split building polygons into separate land covers. Since govmap.gov.il also provides maps with building polygons, it was simpler to remove the buildings from the image initially and add them back in at a later step.
2. Denoising the image: To label each area with its corresponding land cover category (e.g., road, structure, or open area), it was first necessary to divide the image into polygons. Initial separation of land covers was achieved using image colors, where similar colors were assumed to indicate similar land covers. We applied a Non-local Means Denoising smoothing filter to reduce noise, which resulted in more continuous segments.
3. Classifying the images by color values using K-means: After denoising, we classified image pixels into seven classes using the K-means algorithm, based on the image’s RGB color bands. Through preliminary trial and error, we selected K = 7 as the optimal number of clusters, so that each pixel was classified into one of seven classes. This classification is illustrated in the “K-means” and “Color Histogram” panels in Figure 1c,d, where the “K-means” image contains only seven shades of gray.
4. Polygonizing: After segmenting the aerial image with K-means, we transformed the segments into GIS polygons, based on the labeled matrix of the image. For each cat, the process used three inputs: the AOI, the aerial image of the AOI, and a geographical map of the same area with buildings included. This produced a GIS map labeled by color values and ready for further manual processing.

**Table 2:** Land cover classes used for polygon labeling.

Label	Polygon Description
Roads	Paved roads
Structures	Houses, buildings and other structures
Open urban	Open areas inside residential areas with modified terrain (e.g., gardens, parks, planted vegetation, other open spaces), excluding paved roads
Open urban natural	Open areas inside residential areas, with natural terrain
Natural	Open areas outside residential areas, with natural terrain
Agriculture	Open area outside residential area, with agricultural terrain

#### 2.5.3. Manual Labeling of Polygons

We manually classified the land covers found within each cat’s AOI using two attributes: “Is It Green” and “Land cover.” The “Is It Green” attribute distinguished sections of vegetation from all other areas, while the “Land cover” attribute assigned a specific land cover type to each polygon using QGIS 3.8 (Figure 1e,f). For the “Is It Green” attribute, vegetation was separated very effectively by the K-means algorithm and required no manual adjustment. The only manual task was to select the appropriate “green” class (or classes) from the K-means clustering and label them as “green,” with the remainder labeled as “not green.”

**Figure 2:**
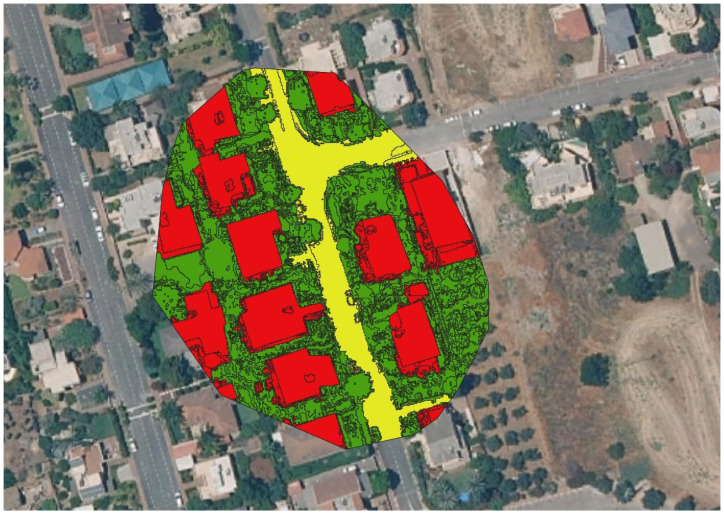
An example of a labeled home range map of a cat. Land cover types are labeled with different colors: Yellow = roads; red = structures; green = open urban; see Table 2 for label definitions.

While the “Is It Green” attribute could be applied automatically, land cover classification required manual input to ensure maximum accuracy. To assign the correct land cover label to each polygon, the original aerial image was visually compared to the polygonised segments. Labels from a preset table (Table 2) were then applied to each polygon. The results for each cat’s land covers are presented in Supplementary Table S1. An example of the final product (a labeled map after manual adjustments) is shown in Figure 2.

### 2.6. Land Cover Availability and Range Bias

To test whether cats’ roaming extents were biased toward or away from particular land cover types, we compared the composition of land covers within each observed AOI to the composition expected under a simple null model of directionally unbiased movement around the cat’s activity center. Conceptually, this approach corresponds to third-order selection in a use/availability framework [41], which is commonly applied in cat studies by comparing land covers “used” by GPS or telemetry fixes to land cover availability within a home-range boundary (for example, using selection ratios, Jacobs’ index, or resource selection functions; [15,16,17,24,25,33]). However, in our analysis, the “use” unit was the realized roaming extent (the AOI polygon), rather than point-level time allocation [34]. We therefore tested whether the placement and shape of the AOI were associated with particular land covers beyond what would be expected from a directionally symmetric baseline.

For each cat, we created an equal-area circular region (hereafter “equal-area circle”) intended to represent isotropic, non-selective roaming around the activity center. The equal-area circle was centered at the median x and median y coordinates of that cat’s cleaned (inlier) GPS fixes and was assigned an area equal to that of the cat’s AOI. Using the same land cover mapping workflow described above, we calculated, separately for the AOI and the equal-area circle, the total area of each land cover class (Table 2) and the total area of vegetation (“Is It Green”). For each cat and each land cover class, we quantified range bias as the difference in land cover area between the AOI and the equal-area circle. Land covers were considered over-represented in the cat’s home range when their area within the AOI exceeded their area within the equal-area circle, and under-represented when the opposite was observed.

It should be noted that the equal-area circle method assumes that within a typical neighborhood, most nearby micro-habitats that are not buildings are accessible to cats (a relatively permeable matrix). Across all cats, differences between AOI and equal-area-circle land cover areas were evaluated using paired tests (see Section 2.8).

### 2.7. Effect of Natural Open Areas on Roaming-Area Size

To test whether the presence of natural open areas influenced roaming-area size, we classified cats into two groups based on whether their AOI contained natural open areas, specifically “Open urban natural” or “Natural” (Table 2). Cats whose AOI included “Agriculture” (9 cats) were excluded from this comparison, as agricultural open spaces differ structurally from natural open spaces, and the sample size in this category was too small for separate analysis.

### 2.8. Statistical Analyses

All analyses were conducted in JMP Pro 14.3.0 (SAS Institute Inc., Cary, NC, USA). Area-based variables (AOI area and land cover areas) were log-transformed prior to analysis when needed, to improve normality. When assumptions for parametric tests were not met after transformation, non-parametric alternatives were used as described below.

We performed three types of tests on the dataset:

1. Matched pairs comparisons between land cover areas within the AOI and land cover areas in the equal-area circle, to test for land cover preferences. Paired *t*-tests were used for parametric variables, and Wilcoxon Signed Rank tests were used for non-parametric variables.
2. Assessment of the impact of individual cat variables (nominal and continuous) on the total size of the cat’s home range, using the Wilcoxon Rank Test and Spearman correlation, respectively.
3. Analysis of the effect of natural open areas on the total size of the cat’s home range, using *t*-tests.

## 3. Results

### 3.1. Home Range Size

**Figure 3:**
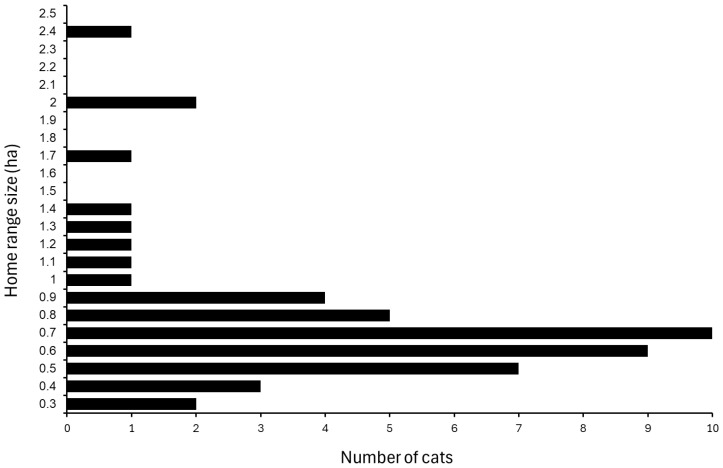
Total home range sizes of free-ranging owned cats.

Across the 49 tracked cats, mean AOI area was 0.851 ± 0.063 ha (SE) and the median was 0.728 ha (Figure 3). The average radius of the equal-area circles was 50.68 ± 1.7 m and the median was 48.15 m.

Cats whose AOIs included natural open areas had larger roaming areas than cats whose AOIs did not. Mean AOI size was 0.981 ± 0.107 ha (median: 0.839 ha) for cats with natural open areas in their AOI (*n* = 24) and 0.725 ± 0.062 ha (median: 0.711 ha) for cats without natural open areas in their AOI (*n* = 25), and this difference was significant (Mann–Whitney U test, U = 192, *p* = 0.031). The average radius of the equal-area circles of cats whose AOIs included/did not include natural open areas were 54.48 ± 3.23 m/46.87 ± 1.08 m, respectively, and the median was 51.68 m and 47.57 m, respectively.

AOI size did not differ by sex (Wilcoxon rank-sum test, Z = −0.781, *p* = 0.435) or by season (Wilcoxon rank-sum test, Z = 0.306, *p* = 0.760). Age showed a moderate significant negative correlation with AOI area, with older cats tending to have smaller roaming areas (Spearman correlation, ρ = −0.407, *p* = 0.004).

There was no visible trend in AOI size from day 2 to day 8. The centroids of the daily AOIs were very close to one another (Supplementary Material S2).

### 3.2. Land Cover Bias (AOI vs. Equal-Area Circle)

Comparisons between the land cover composition of each cat’s AOI and the corresponding equal-area circle showed no evidence of consistent attraction to or avoidance of any land cover class. For all land cover categories, the areas within the AOI and the equal-area circle did not differ significantly (Table 3).

## 4. Discussion

Cats in this study exhibited remarkably small home ranges, with most individuals restricting their outdoor activity to within just a few dozen meters of their homes. This limited roaming is consistent with multiple GPS-based studies, which likewise report that free-roaming cats typically spend the majority of their time in close proximity to their residence [6,15,18,25]. While our findings are at the lower end of the reported range for free-roaming cats, other studies have documented substantially larger home ranges, particularly outside dense urban cores or where cats have access to expansive green or semi-natural habitats [2,16,26,27]. Such variation among studies likely reflects both genuine ecological differences (e.g., housing density, landscape permeability, cat density, availability of vegetative cover and prey) and methodological distinctions [2,10,18,25,33].

Most cats adapted well to the harness-mounted GPS devices and completed the tracking period. Additionally, there was no visible trend in AOI size over time, and cats demonstrated a consistent center of activity (Supplementary Material S2). However, it is important to acknowledge a potential selection bias: restricting the sample to harness-tolerant cats may have influenced the results, as these individuals could differ systematically in movement behavior from cats that were less tolerant of the devices.

The modest but statistically significant increase in home range size for cats whose roaming areas included natural open habitats aligns with a well-documented pattern: where accessible open or semi-natural areas exist near their homes, cats often expand their home ranges. Comparable effects have been observed in studies where cats situated at urban edges or near reserves, greenspaces, or boundary habitats tended to range farther than those embedded within denser residential matrices [15,25,26,27]. One interpretation is that open habitats provide continuous movement corridors and greater hunting opportunities, allowing cats that utilize them to extend their overall ranging area. Conversely, densely built residential areas, high road densities, and fragmented micro-habitats may constrain movement and keep home ranges compact, even when outdoor access is unrestricted [18,25].

No significant differences in home range size were detected between female and male cats, nor between “summer” and “winter” tracking sessions. This is consistent with several GPS or telemetry studies of free-roaming, neutered cats, which also report weak or non-significant sex effects on home range extent [25,26,33]. Similarly, the lack of seasonal variation in home range size mirrors findings from some prior studies [33], though not all [26]. The absence of a seasonal effect in our study may be attributable to the region’s very mild winter. In contrast, age showed a negative association with home range size in our data, with older cats tending to have smaller roaming areas, a pattern also reported in large, multi-site datasets of owned cats [18,30,31].

Across the 49 tracked cats, we found no evidence of consistent attraction to or avoidance of any mapped land cover class: the composition of land covers within realized home ranges did not differ from what would be expected under the equal-area circle baseline. In other words, at the fine spatial scale of these cats’ roaming extents, the placement of home ranges was not systematically shifted toward or away from particular land cover types, despite the detailed land cover mapping used in this study.

This result is consistent with some prior studies [35], but contrasts with others that reported non-random habitat use within larger home ranges of free-roaming owned cats. For example, some studies have found disproportionate use of gardens, vegetated greenspaces, and structural edges [24,27,33], while others report that cats spend much of their time in disturbed habitats near buildings [6,15,18]. Recent urban GPS studies also identify habitat selection patterns, though the direction and strength of such selection vary between study systems [16,17,27,33].

Taken together, our results suggest that, at least for the small roaming extents observed in this study, micro-scale land cover composition may not be a strong determinant of where cats establish their roaming footprint, even when land cover is mapped in considerable detail.

## 5. Conclusions

In this study, we combined GPS tracking of owned cats with high-resolution, up-to-date land-cover mapping to test whether selection for specific land-cover types determines the shape and placement of cats’ home ranges. Overall, home ranges were very small, and cats with natural and semi-natural habitats near their homes tended to range over larger areas than cats without such habitat nearby. However, cats were not disproportionately drawn to these open habitats, and the land-cover composition within each home range largely reflected what was locally available. Even with fine-scale, accurate land-cover classification, we found no evidence of selection for particular land-cover types within the cats’ relatively small home ranges.

Taken together, these findings provide some indication that roaming by owned cats may not be primarily driven by attempts to maximize hunting opportunities; instead, predation on local fauna may be incidental, occurring when cats encounter prey while moving within their home ranges for reasons other than actively seeking prey. Consequently, in areas where cats have very small home ranges (as in this study), buffer zones in which cat ownership is prohibited, aimed at reducing the impact of owned cats on natural habitats, may not need to be extensive, as cats may not preferentially venture into these areas. Conversely, in areas where cats have larger home ranges (Table 1), buffers may be essential for protecting natural habitats. It should be noted, however, that our conclusions are based on the results of a study that was conducted in a small region, and that did not record hunting data. Therefore, for conservation practitioners to consider adopting narrower buffer zones, which could be easier to implement while still effectively protecting wild populations of small animals, future studies that use fine-scale, up-to-date and accurate land-cover classification should be conducted across various different habitats and climatic zones using various additional tracking methods.

## 6. Limitations

Several limitations should be considered when interpreting the results of our study. First, our analyses evaluate whether the composition of land covers within each cat’s realized home range (AOI) differs from an equal-area circular baseline, rather than quantifying time-weighted habitat use. In principle, a time-use approach would have been highly informative, as it could have distinguished brief transits from sustained use of particular land covers and provided a more direct measure of habitat selection. However, estimating time-use within land-cover polygons was not feasible for two reasons: (i) The GPS tracking units were set to “motion-detection mode,” which prolonged battery life but limited recordings to periods when cats were moving; therefore, the data could not be used to quantify time use. (ii) Since the areas of home ranges and land-cover polygons were very small, and GPS error was of the same order as many polygon boundaries, point-to-polygon assignment would have been unreliable.

Second, although the harness-mounted GPS (also used in the Kays et al. 2020 [18] large-scale study) was selected to improve comfort relative to collar-hung devices, we cannot exclude the possibility that wearing the harness influenced behavior, including movement patterns and space use, in ways that we were unaware of and therefore could not account for.

Third, our analysis method assumes that, aside from buildings and structures, cats could access all land covers within their home ranges. This assumption is reasonable because cats are generally capable climbers and can likely pass or circumvent many obstacles in residential settings, and because large, clearly impassable barriers such as highways were unlikely to be relevant here since none of the tracked cats lived near such features. However, impermeable micro-barriers (e.g., tall fences, gates) that are typically not visible in aerial imagery, and therefore could not be accounted for, could still have constrained movement by blocking passage.

Finally, this study did not measure hunting attempts, prey capture, wildlife responses, or time-weighted habitat use. Therefore, it cannot support strong mechanistic claims regarding whether roaming is motivated by hunting, whether predation is “incidental,” or whether buffer zones near natural habitats can be reduced; at most, our findings are consistent with these possibilities and indicate priorities for future work.

## Figures and Tables

**Table 1:** Home range areas of owned house cats in past GPS-tracking studies. Various estimators were used for estimating home ranges.

Study	Study Location	*n*	MCP Estimate (ha)	Kernel/Modern Estimate (ha)
This study	Israel	49	Mean 0.85 Median 0.73 95% MCP	—
van Heezik et al. 2010 [24]	New Zealand	32	Mean 3.2 (median 2.2) 100% MCP	Mean 1.5 (median 0.6) KDE
Hanmer et al. 2017 [25]	UK	38	Mean 1.18 (median 0.95) 95% MCP	Mean 1.66 (median 1.28) 95% KDE
Kays et al. 2020 [18]	Australia; New Zealand; USA; UK; Canada; Denmark	925	—	Mean 3.6 95% KDE
Pisanu et al. 2020 [26]	France	30	—	Rural: mean 3.5 Suburban: 2.1 Urban: 1.4 RD-MKDE
Cecchetti et al. 2022 [1]	UK	72	—	Median: 1.51 (IQR 0.76–2.38) 95% AKDE
Pirie et al. 2022 [27]	UK	79	—	Boundary: mean 3.42 Non-boundary: mean 2.01 95% KDE
Jensen et al. 2022 [2]	Denmark	97	—	Median 5.0 (IQR 2.9–8.5) 95% BBKDE
Bischof et al. 2022 [6]	Norway	92	—	Mean 2.6 (IQR 0.7–3.2) 95% BBMM
Simmons et al. 2023 [11]	South Africa	23	Summer: mean 31.65 Winter: 3.44 100% MCP	Summer: mean 3.00 Winter: 0.87 95% KDE
Dunford et al. 2024 [16]	UK	56	Mean 8.63 100% MCP	—
Pyott et al. 2024 [17]	Canada	42	Median 4.4 (range 0.34–38.45) 100% MCP	Median 1.36 (range 0.27–11.18) 95% KDE
Palomares & Sanglas 2025 [10]	Spain	64	Mean 66.1 (100% MCP) Mean 7.5 (95% MCP)	Mean 10.9 (95% KDE) Mean 1.6 (50% KDE)

MCP: Minimum Convex Polygon; KDE: Kernel Density Estimator; RD-MKDE: Residence-Distance Movement-based Kernel Density Estimator; BBKDE: Brownian bridge Kernel Density Estimator; IQR: Interquartile Range; BBMM: Brownian Bridge Movement Model; AKDE: Autocorrelated Kernel Density Estimator.

**Table 3:** Land cover preferences of owned cats. Descriptions of land cover classes (rows 1–6) are given in Table 2. Lines 7–11 contain a general classification to green/not green, and different combinations of land cover classes. All data were log-transformed and analyzed using paired-samples *t*-tests (test statistic t) or Wilcoxon Signed Rank test (test statistic z). *n* = the number of cats that had that land cover class within their home range or within their corresponding equal-area circle. The detailed, untransformed data are given in Supplementary Table S1.

	Variable	Description	*n*	Equal-Area Circle Mean	Home Range Mean	Test Statistic	*p*-Value
1	Roads	See Table 2	49	6.573	6.632	z = 26.5	0.795
2	Structures		49	7.234	7.223	z = 1.5	0.988
3	Open urban		46	8.055	8.043	z = 30.5	0.742
4	Open urban natural		17	7.487	7.42	t = −0.48	0.637
5	Natural		10	7.207	7.145	z = −1.5	0.921
6	Agriculture		9	7.112	7.145	t = 0.271	0.792
7	Green	All areas with vegetation	49	8.014	8.003	t = −0.762	0.449
8	Not green	All areas with no vegetation	49	8.39	8.405	t = 1.492	0.142
9	Open all	All open areas (Open Urban + Natural + Agriculture + Open urban natural)	49	8.542	8.54	t = 0.329	0.743
10	Open urban all	All open areas within settlement borders (Open urban + Open urban natural)	48	8.388	8.391	z = −62	0.530
11	Open outside settlement	All open areas outside settlement borders (Natural + Agriculture)	19	7.162	7.145	z = −2	0.953

## Data Availability

The raw data supporting the conclusions of this article will be made available by the corresponding author upon request. The code used in the study is openly available on GitHub at https://github.com/NoyKad/cat-landcover-effects (accessed on 22 January 2026).

## Supplementary Materials

The following supporting information can be downloaded at https://www.mdpi.com/article/10.3390/ani16060864/s1, Figure S1: An example of the removal of 5% of GPS fixes furthest from the center; Table S1: Individual cat data; Supplementary Material S2: Temporal changes in AOI size and shape during the tracking period.
