# Supplementary material for: Owned House Cats Show No Preference for Specific Land Cover Types When Roaming Outdoors
Source: Animals (Basel). 2026 Mar 10;16(6):864. doi: 10.3390/ani16060864 (PMC13023274; doi:10.3390/ani16060864)
Supplement: Supplementary file 1 [file animals-16-00864-s001.zip › Supplementary material S1.pdf]

Supplementary material S1

Roaming owned house cats show no preference for specific land cover types

Lyan Wolovelsky, Noy Kadosh and Moshe Gish

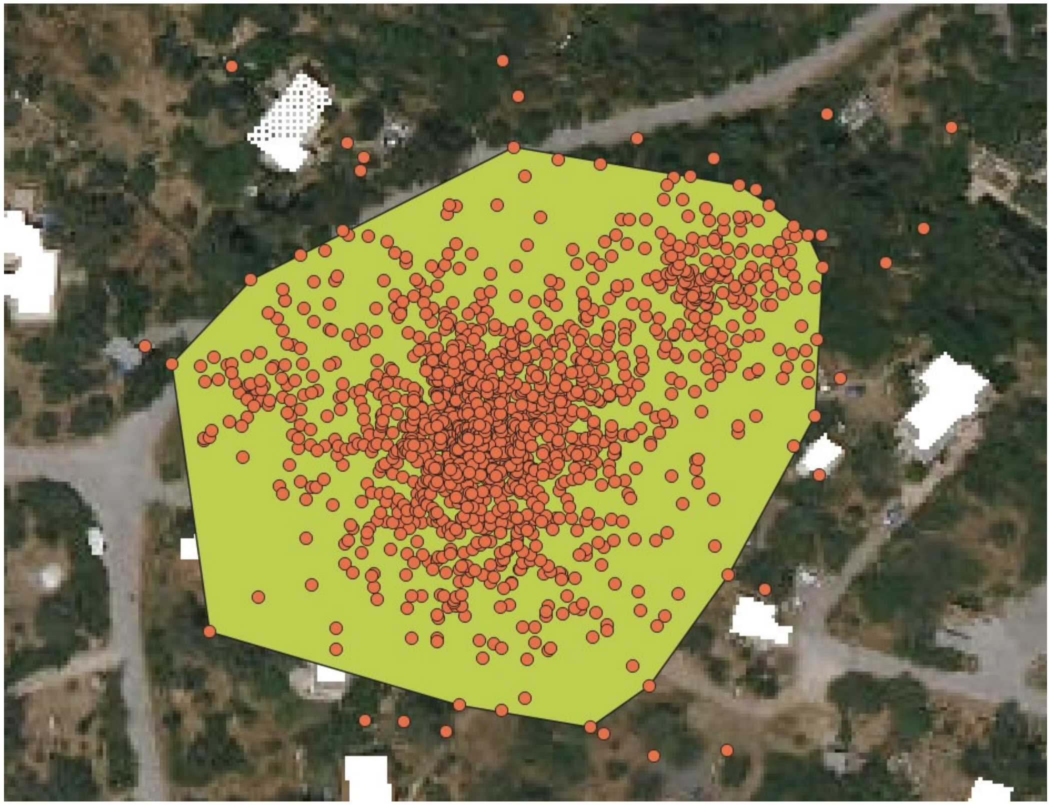

Figure S1: An example of the removal of 5% of GPS fixes furthest from the center.

Table S1: Individual cat data.

Cat #	Age (years)	Sex	Weight (kg)	Season	Log Start	Log End	Settlement name	Total home range area (m2)	%green	Open urban (m^2)	Open urban natural (m^2)	Natural (m^2)	Agriculture (m^2)	Structures (m^2)	Roads (m^2)
1	8	f	> 3	Summer	14/06/2019	23/06/2019	Haifa	3176.86	50.89	1197.08	0	795.81	0	1053.81	130.16
2	12	m	6	summer	01/07/2019	09/07/2019	Kamon	3215.78	18.65	0	81.68	2443.71	0	300.36	390.02
3	3.5	m	3.5	summer	13/09/2019	20/09/2019	Givat Ada	4058.34	56.4	2233.96	0	0	919.34	762.76	142.29
4	8.5	f	3	summer	29/07/2019	06/08/2019	Kamon	4818.17	49.72	3568.41	0	582.49	0	583.32	83.96
5	6	f	> 3	winter	23/02/2020	03/03/2020	Shadmot Dvora	4860.04	35.04	2485.97	0	0	0	1882.17	491.9
6	10	m	4.3	summer	09/06/2019	17/06/2019	Hod Hasharon	5058.78	35.71	3540.23	0	0	0	810.35	708.21
7	3	f	3.5	summer	08/07/2019	15/07/2019	Nazareth Illit	5163.44	29.41	3971.32	0	0	0	575.97	616.15
8	6	f	> 3	winter	23/02/2020	03/03/2020	Shadmot Dvora	5278.18	34.25	2361.08	0	0	0	2287.26	629.84
9	3.5	f	3.5	summer	31/08/2019	07/09/2019	Givat Ada	5350.71	53.37	3144.34	0	0	973.98	929.69	302.69
10	12	f	5	summer	03/07/2019	12/07/2019	Sde Yaakov	5441.57	43.86	2711.49	0	0	1012.12	717.51	1000.45
11	6	f	4	summer	31/6/19	09/07/2019	Givat Ada	5592.94	54.23	3332.92	0	0	1018.45	916.38	325.2
12	10	m	4	summer	12/06/2019	20/06/2019	Nataf	5920.77	44.32	499.11	4881.67	0	0	175.49	364.5
13	10.5	m	> 3	summer	17/07/2019	27/07/2019	Modiin	6027.65	23.42	2825.75	0	0	0	2396.91	805
14	8	m	5	winter	17/02/2020	25/02/2020	Kfar Tavor	6093.83	35.51	4168.67	91.2	0	0	1447.29	386.67
15	6	f	4.5	winter	04/12/2019	12/12/2019	Alonei Aba	6095.8	41.15	3918.9	0	0	0	1583.27	593.63
16	1	m	5.7	winter	30/03/2019	09/04/2019	Nahariya	6279.72	51.98	4458.73	0	0	0	1414.29	406.7
17	6	m	7.5	winter	04/12/2019	12/12/2019	Alonei Aba	6473.95	39.2	4139.09	0	0	0	1721.86	613.01
18	1	m	8	winter	05/02/2020	15/02/2020	Ramot Hashavim	6656.61	42.34	4804.79	0	0	0	1484.31	367.53
19	2	m	> 3	winter	30/01/2020	09/02/2020	Kfar Tavor	6836.19	45.49	1542.98	158.88	0	2290.44	1273.12	1570.77
20	2	f	3.5	summer	15/09/2019	23/09/2019	Kamon	6841.12	20.02	3075.04	456.11	1224.03	0	774.37	1311.59
21	2	f	> 3	summer	06/07/2019	15/07/2019	Nataf	6847.98	24.04	0	2994.03	2800.3	0	184.72	868.94

22	3	f	> 3	summer	28/08/2019	05/09/2019	Haifa	7109.86	42.23	3108.45	0	0	0	3564.18	437.23
23	6	m	7.2	summer	19/07/2019	27/07/2019	Kiryat Tivon	7138.64	57.85	1201.97	4170.04	0	0	1417.73	348.9
24	2	f	3.5	summer	27/06/2019	05/07/2019	Nataf	7224.32	55.76	5777.97	0	0	0	1031.26	415.09
25	2	f	3	winter	13/01/2020	20/01/2020	Kamon	7284.61	13.89	1580.67	0	3536.59	0	816.78	1350.57
26	1	m	4.7	winter	30/03/2019	11/04/2019	Nahariya	7626.56	53.67	5427.02	0	0	0	1806.53	393.01
27	6	m	7	summer	15/06/2019	22/06/2019	Haifa	7668.25	42.14	3805.53	0	0	0	3323.31	539.41
28	3	f	4	summer	14/06/2019	22/06/2019	Haifa	7673.54	35.24	2691.97	0	0	0	3837.65	1143.93
29	5	m	5.5	summer	15/06/2019	22/06/2019	Haifa	7703.28	44.73	3980.34	0	0	0	2314.95	1408
30	1	f	4	winter	21/02/2020	01/03/2020	Sharona	7794.95	45.85	2611.07	1839.84	0	972.14	1378.42	993.47
31	8	m	> 3	summer	18/07/2019	26/08/2019	Manof	7832.55	43.79	3973.28	0	1261.81	0	939.47	1657.98
32	10	m	6	summer	20/06/2019	29/06/2019	Sde Yaakov	8052	50.03	4893.07	0	0	186.13	1242.96	1729.84
33	3	m	> 3	summer	26/04/2019	08/05/2019	Mesilot	8147.58	40.15	5031.31	0	0	0	1754.31	1361.96
34	2.5	f	4	summer	19/07/2019	27/09/2019	Kamon	8391.53	29.12	3132.24	0	3457.91	0	723.7	1077.68
35	5	f	3.5	winter	07/02/2020	14/02/2020	Kfar Tavor	8690.4	35.47	5659.99	0	0	0	2088.32	942.09
36	1.3	m	> 3	winter	22/01/2020	30/01/2020	Kfar Tavor	8923.24	37.99	5659.55	0	0	0	1890.14	1373.55
37	8	m	6	winter	06/02/2020	14/02/2020	Kfar Tavor	9440.8	28.92	4703.37	0	68.8	0	3376.71	1291.93
38	4	f	> 3	summer	15/07/2019	23/07/2019	Manof	9453.56	54.67	4690.7	1374.1	0	0	1619.64	1769.12
39	2	f	5.3	summer	16/06/2019	25/06/2019	Ramot Menashe	9706.24	46.98	7660.77	0	0	0	1586.58	458.89
40	5	m	4	summer	05/09/2019	13/09/2019	Kamon	9802.12	40.67	250.13	5379.53	2614.56	0	1022.48	535.42
41	9	m	6.5	winter	17/02/2020	25/02/2020	Kfar Tavor	10003.02	28.23	4605.58	2845.54	0	0	2272.6	279.3
42	6	m	> 3	winter	30/01/2020	09/02/2020	Kfar Tavor	11425.93	45.47	2823.79	69.1	0	3640.87	2360.72	2531.46
43	1	m	> 3	Summer	11/07/2019	19/07/2019	Haifa	12666.8	59.34	626.81	8919.89	0	0	1966.53	1153.58
44	2.5	f	4.5	winter	20/01/2020	30/01/2020	Kfar Tavor	13795.22	21.16	2053.59	6153.65	0	0	2568.83	3019.15
45	1.5	m	> 3	summer	24/06/2019	03/07/2019	Haifa	14182.74	56.29	1426.92	9157.02	0	0	2391.58	1207.22
46	1	m	4.5	summer	26/07/2019	04/08/2019	Nazareth Illit	17396.29	34.63	10885.44	647.52	0	0	3003.26	2860.06
47	8	m	5.7	summer	22/07/2019	30/07/2019	Ein Hanatziv	20284.44	28	7515.75	840.4	0	6099.18	2998.86	2830.24
48	2	f	> 3	summer	14/06/2019	21/06/2019	Haifa	20456.19	52.83	5377.38	9003.4	0	0	3067.02	3008.4
49	1	f	> 3	summer	22/07/2019	30/07/2019	Nataf	24786.11	44.45	0	20681.7	0	0	1629.23	2475.18
