# Supplementary material for: Owned House Cats Show No Preference for Specific Land Cover Types When Roaming Outdoors
Source: Animals (Basel). 2026 Mar 10;16(6):864. doi: 10.3390/ani16060864 (PMC13023274; doi:10.3390/ani16060864)
Supplement: Supplementary file 1 [file animals-16-00864-s001.zip › Supplementary material S2.pdf]

Supplementary material S2

Roaming owned house cats show no preference for specific land cover types

Lyan Wolovelsky, Noy Kadosh and Moshe Gish

A basic qualitative assessment of temporal drift in cat movement. For each cat, the AOI for each tracking day is displayed in a different color. No clear temporal trend in AOI size is evident. However, the first day of tracking (the acclimation day) appears to yield a smaller AOI for many cats; data from day 1 were excluded from the analyses. From day 2 onward, cat behavior did not appear to be affected by the GPS harness. Notably, the centroids of the daily AOIs are very close to one another, indicating a consistent center of activity. The resolutions of the aerial photographs were deliberately reduced to protect participant privacy.

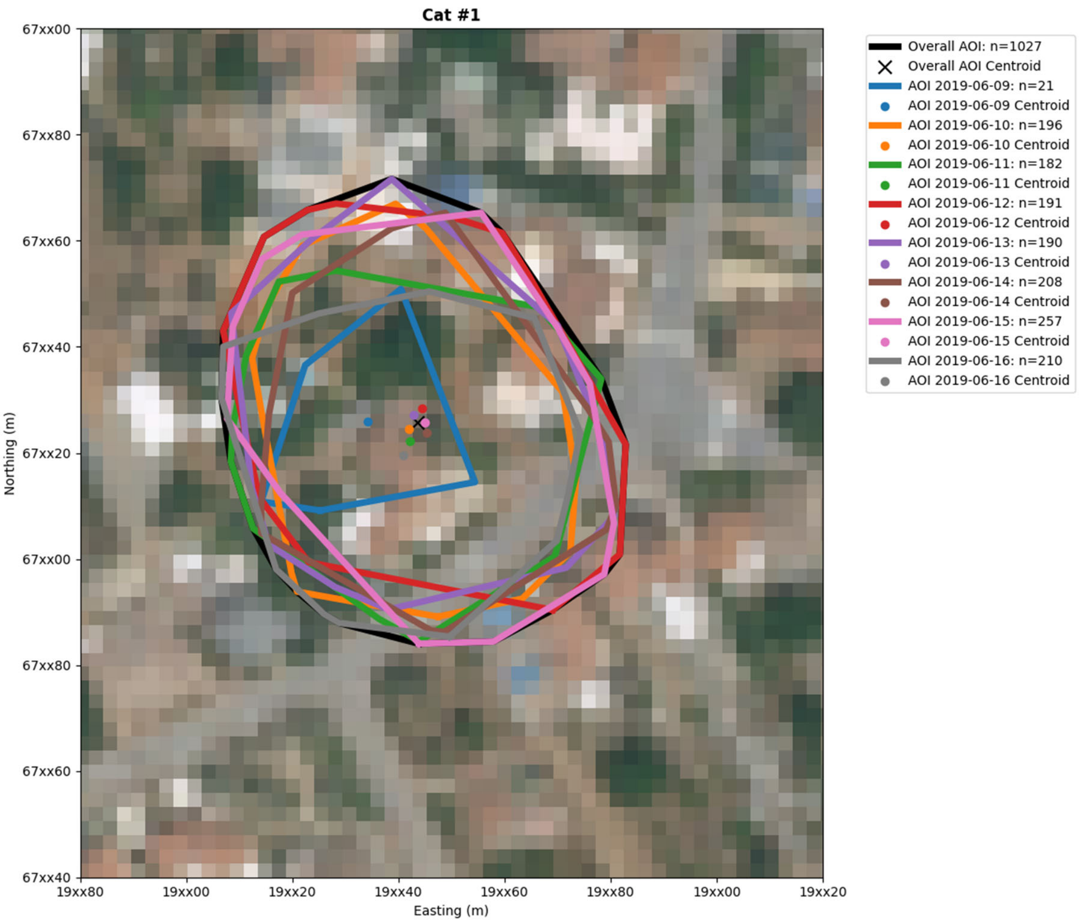

Cat #1
Overall AOI: n=1027
Overall AOI Centroid
AOI 2019-06-09: n=21
AOI 2019-06-09 Centroid
AOI 2019-06-10: n=196
AOI 2019-06-10 Centroid
AOI 2019-06-11: n=182
AOI 2019-06-11 Centroid
AOI 2019-06-12: n=191
AOI 2019-06-12 Centroid
AOI 2019-06-13: n=190
AOI 2019-06-13 Centroid
AOI 2019-06-14: n=208
AOI 2019-06-14 Centroid
AOI 2019-06-15: n=257
AOI 2019-06-15 Centroid
AOI 2019-06-16: n=210
AOI 2019-06-16 Centroid
Northing (m)
67xx00
67xx80
67xx60
67xx40
67xx20
67xx00
67xx80
67xx60
67xx40
Easting (m)
19xx80
19xx00
19xx20
19xx40
19xx60
19xx80
19xx00
19xx20

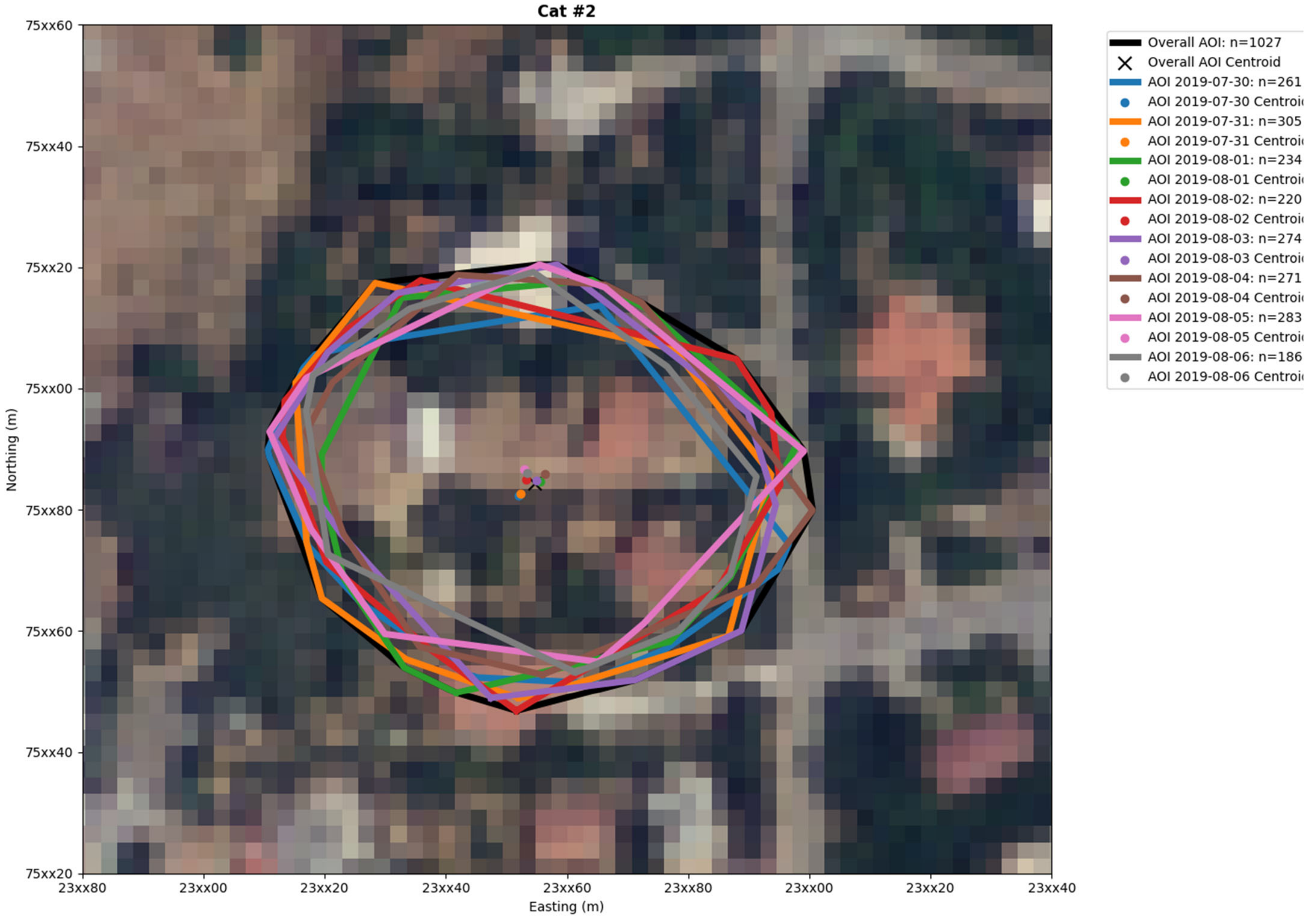

Cat #2
Overall AOI: n=1027
Overall AOI Centroid
AOI 2019-07-30: n=261
AOI 2019-07-30 Centroi
AOI 2019-07-31: n=305
AOI 2019-07-31 Centroi
AOI 2019-08-01: n=234
AOI 2019-08-01 Centroi
AOI 2019-08-02: n=220
AOI 2019-08-02 Centroi
AOI 2019-08-03: n=274
AOI 2019-08-03 Centroi
AOI 2019-08-04: n=271
AOI 2019-08-04 Centroi
AOI 2019-08-05: n=283
AOI 2019-08-05 Centroi
AOI 2019-08-06: n=186
AOI 2019-08-06 Centroi
Northing (m)
75xx60
75xx40
75xx20
75xx00
75xx80
75xx60
75xx40
75xx20
Easting (m)
23xx80
23xx00
23xx20
23xx40
23xx60
23xx80
23xx00
23xx20
23xx40

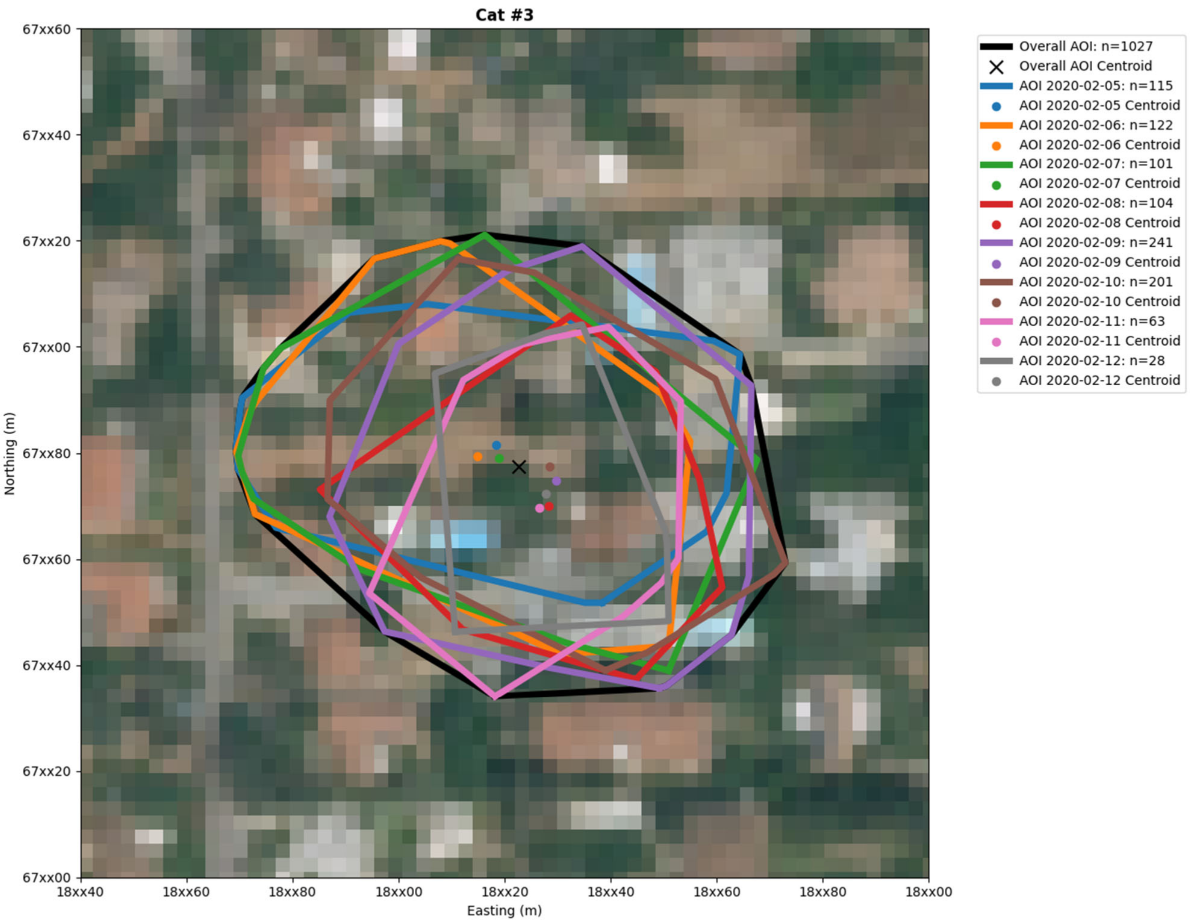
Cat #3
67xx60
67xx40
67xx20
67xx00
67xx80
67xx60
67xx40
67xx20
67xx00
Northing (m)
18xx40
18xx60
18xx80
18xx00
18xx20
18xx40
18xx60
18xx80
18xx00
Easting (m)
Overall AOI: n=1027
Overall AOI Centroid
AOI 2020-02-05: n=115
AOI 2020-02-05 Centroid
AOI 2020-02-06: n=122
AOI 2020-02-06 Centroid
AOI 2020-02-07: n=101
AOI 2020-02-07 Centroid
AOI 2020-02-08: n=104
AOI 2020-02-08 Centroid
AOI 2020-02-09: n=241
AOI 2020-02-09 Centroid
AOI 2020-02-10: n=201
AOI 2020-02-10 Centroid
AOI 2020-02-11: n=63
AOI 2020-02-11 Centroid
AOI 2020-02-12: n=28
AOI 2020-02-12 Centroid

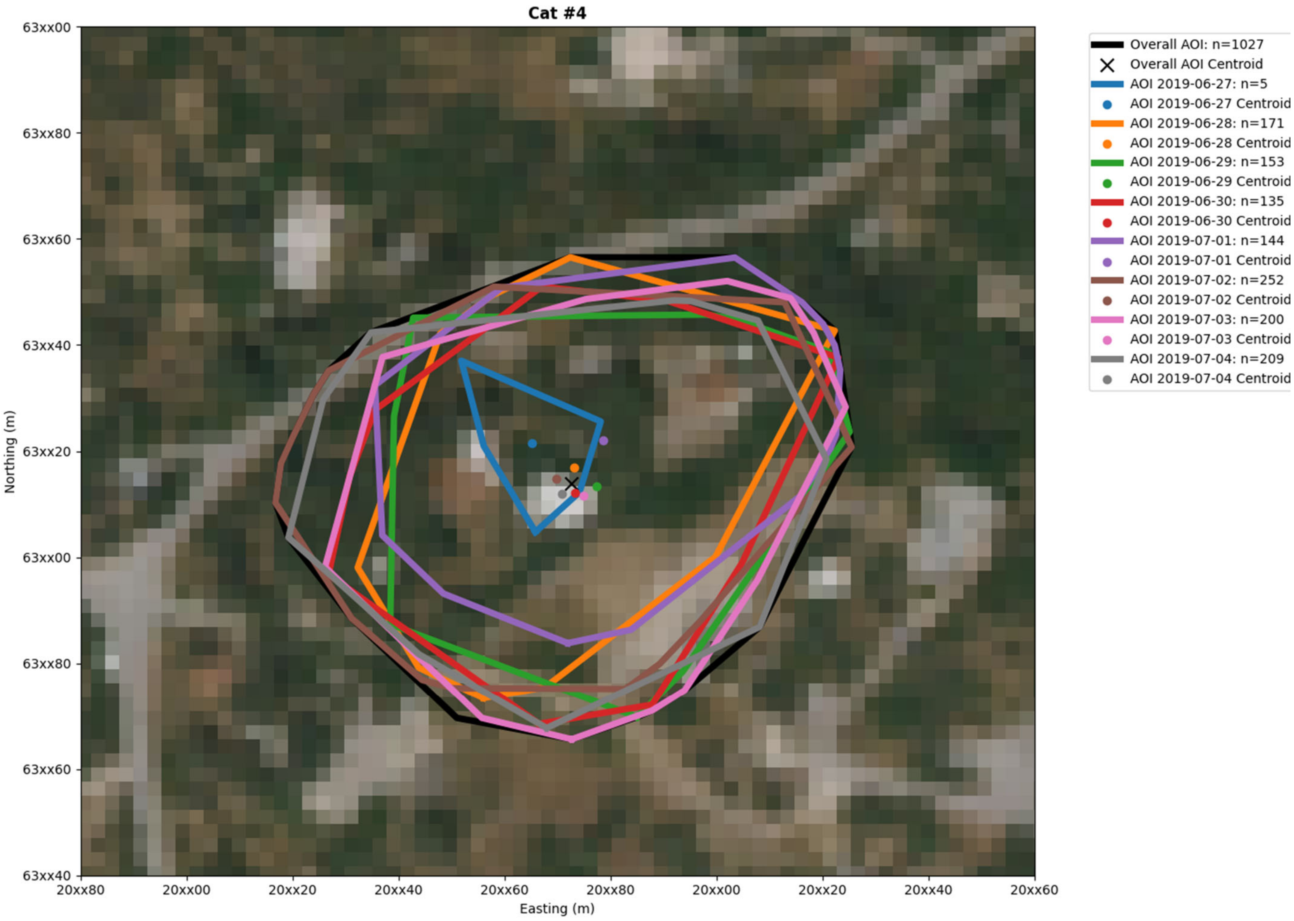
Cat #4
63xx00
63xx80
63xx60
63xx40
63xx20
63xx00
63xx80
63xx60
63xx40
Northing (m)
20xx80
20xx00
20xx20
20xx40
20xx60
20xx80
20xx00
20xx20
20xx40
20xx60
Easting (m)
Overall AOI: n=1027
Overall AOI Centroid
AOI 2019-06-27: n=5
AOI 2019-06-27 Centroid
AOI 2019-06-28: n=171
AOI 2019-06-28 Centroid
AOI 2019-06-29: n=153
AOI 2019-06-29 Centroid
AOI 2019-06-30: n=135
AOI 2019-06-30 Centroid
AOI 2019-07-01: n=144
AOI 2019-07-01 Centroid
AOI 2019-07-02: n=252
AOI 2019-07-02 Centroid
AOI 2019-07-03: n=200
AOI 2019-07-03 Centroid
AOI 2019-07-04: n=209
AOI 2019-07-04 Centroid

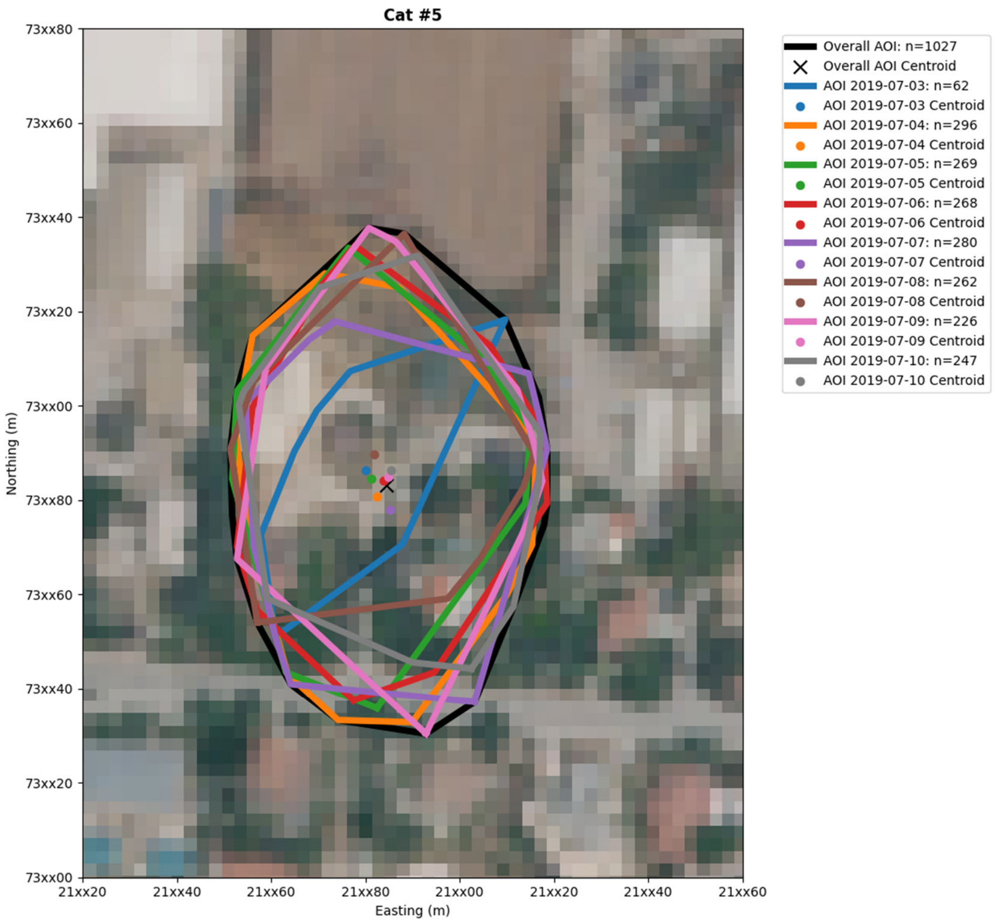
Cat #5
73xx80
73xx60
73xx40
73xx20
73xx00
73xx80
73xx60
73xx40
73xx20
73xx00
Northing (m)
21xx20
21xx40
21xx60
21xx80
21xx00
21xx20
21xx40
21xx60
Easting (m)
Overall AOI: n=1027
Overall AOI Centroid
AOI 2019-07-03: n=62
AOI 2019-07-03 Centroid
AOI 2019-07-04: n=296
AOI 2019-07-04 Centroid
AOI 2019-07-05: n=269
AOI 2019-07-05 Centroid
AOI 2019-07-06: n=268
AOI 2019-07-06 Centroid
AOI 2019-07-07: n=280
AOI 2019-07-07 Centroid
AOI 2019-07-08: n=262
AOI 2019-07-08 Centroid
AOI 2019-07-09: n=226
AOI 2019-07-09 Centroid
AOI 2019-07-10: n=247
AOI 2019-07-10 Centroid

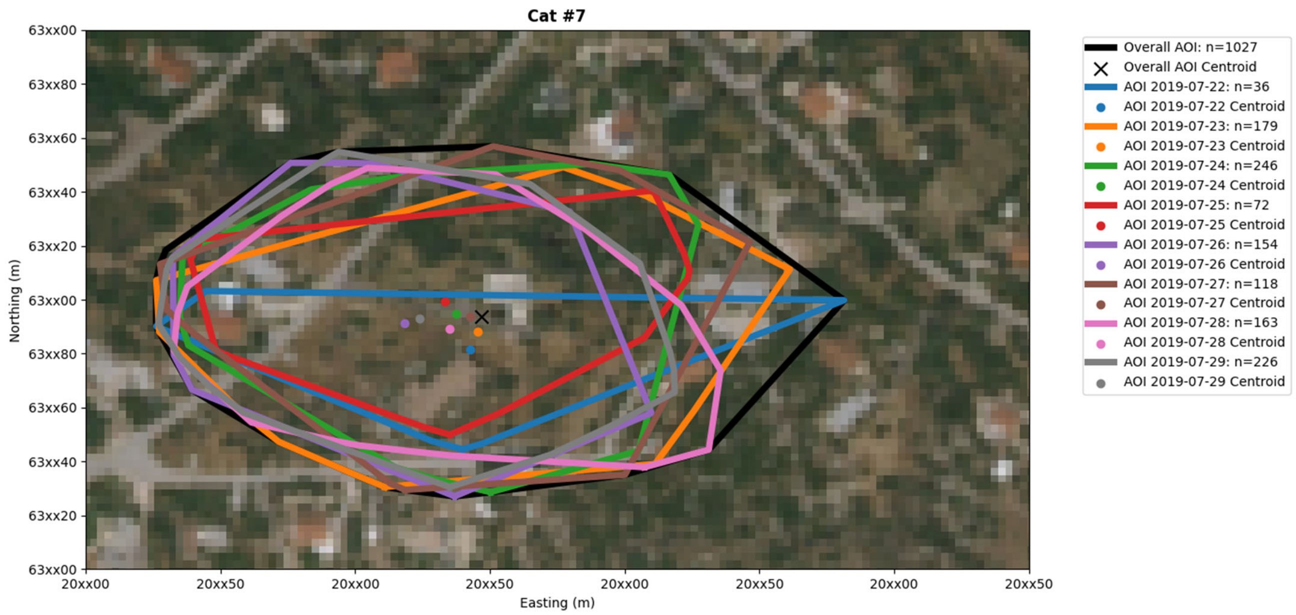
Cat #7
63xx00
63xx80
63xx60
63xx40
63xx20
63xx00
63xx80
63xx60
63xx40
63xx20
63xx00
Northing (m)
20xx00
20xx50
20xx00
20xx50
20xx00
20xx50
20xx00
20xx50
Easting (m)
Overall AOI: n=1027
Overall AOI Centroid
AOI 2019-07-22: n=36
AOI 2019-07-22 Centroid
AOI 2019-07-23: n=179
AOI 2019-07-23 Centroid
AOI 2019-07-24: n=246
AOI 2019-07-24 Centroid
AOI 2019-07-25: n=72
AOI 2019-07-25 Centroid
AOI 2019-07-26: n=154
AOI 2019-07-26 Centroid
AOI 2019-07-27: n=118
AOI 2019-07-27 Centroid
AOI 2019-07-28: n=163
AOI 2019-07-28 Centroid
AOI 2019-07-29: n=226
AOI 2019-07-29 Centroid

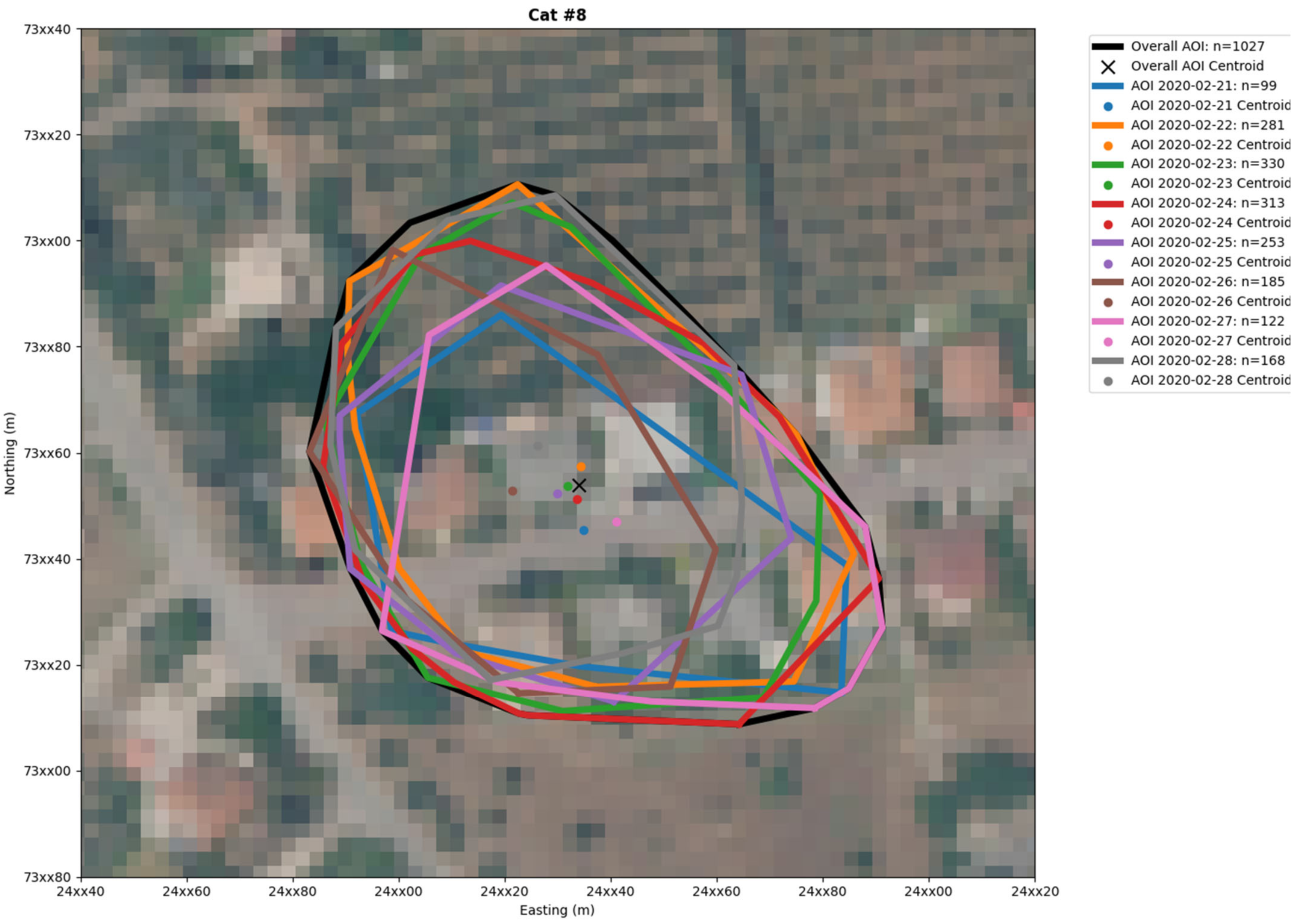
Cat #8
Overall AOI: n=1027
Overall AOI Centroid
AOI 2020-02-21: n=99
AOI 2020-02-21 Centroid
AOI 2020-02-22: n=281
AOI 2020-02-22 Centroid
AOI 2020-02-23: n=330
AOI 2020-02-23 Centroid
AOI 2020-02-24: n=313
AOI 2020-02-24 Centroid
AOI 2020-02-25: n=253
AOI 2020-02-25 Centroid
AOI 2020-02-26: n=185
AOI 2020-02-26 Centroid
AOI 2020-02-27: n=122
AOI 2020-02-27 Centroid
AOI 2020-02-28: n=168
AOI 2020-02-28 Centroid
Northing (m)
Easting (m)
73xx40
73xx20
73xx00
73xx80
73xx60
73xx40
73xx20
73xx00
73xx80
24xx40
24xx60
24xx80
24xx00
24xx20
24xx40
24xx60
24xx80
24xx00
24xx20

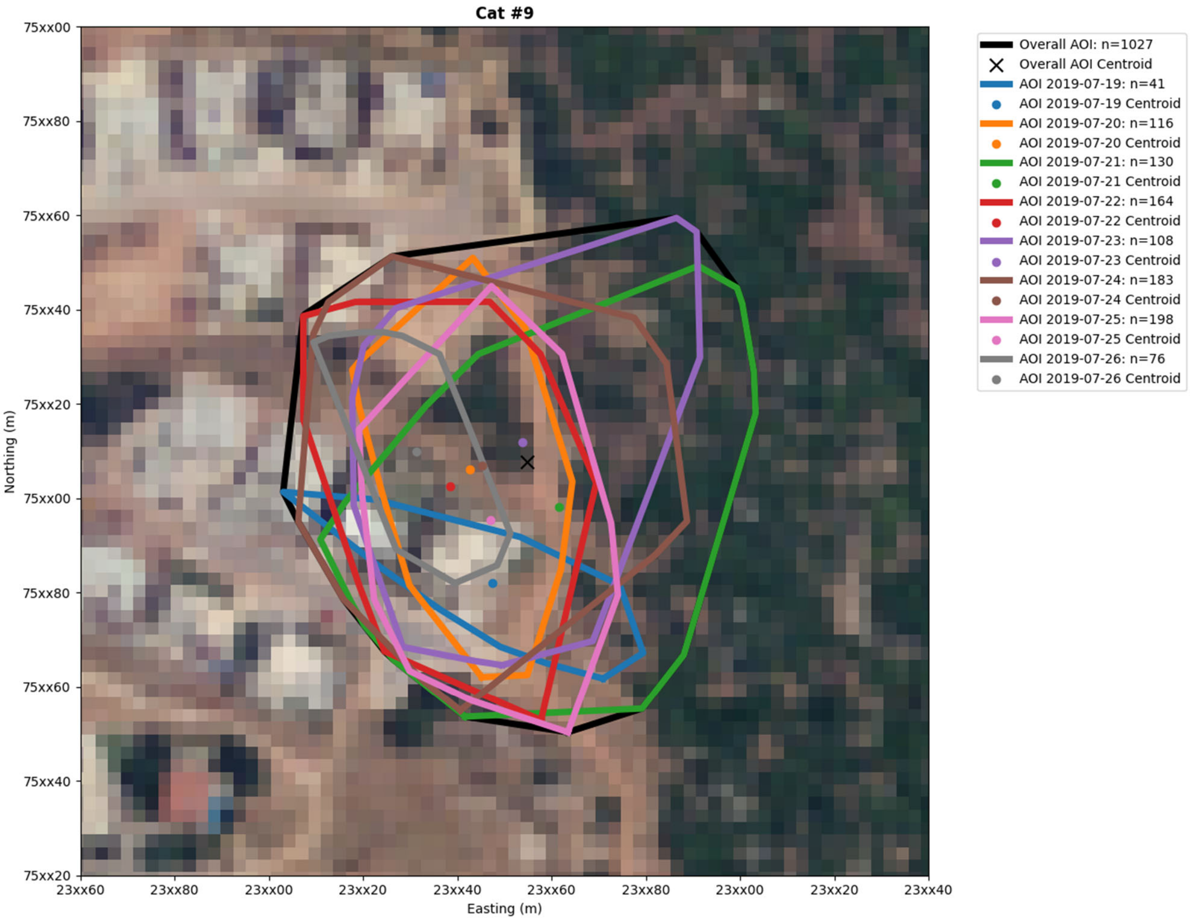
Cat #9
Overall AOI: n=1027
Overall AOI Centroid
AOI 2019-07-19: n=41
AOI 2019-07-19 Centroid
AOI 2019-07-20: n=116
AOI 2019-07-20 Centroid
AOI 2019-07-21: n=130
AOI 2019-07-21 Centroid
AOI 2019-07-22: n=164
AOI 2019-07-22 Centroid
AOI 2019-07-23: n=108
AOI 2019-07-23 Centroid
AOI 2019-07-24: n=183
AOI 2019-07-24 Centroid
AOI 2019-07-25: n=198
AOI 2019-07-25 Centroid
AOI 2019-07-26: n=76
AOI 2019-07-26 Centroid
Northing (m)
Easting (m)
75xx00
75xx80
75xx60
75xx40
75xx20
75xx00
75xx80
75xx60
75xx40
75xx20
23xx60
23xx80
23xx00
23xx20
23xx40
23xx60
23xx80
23xx00
23xx20
23xx40

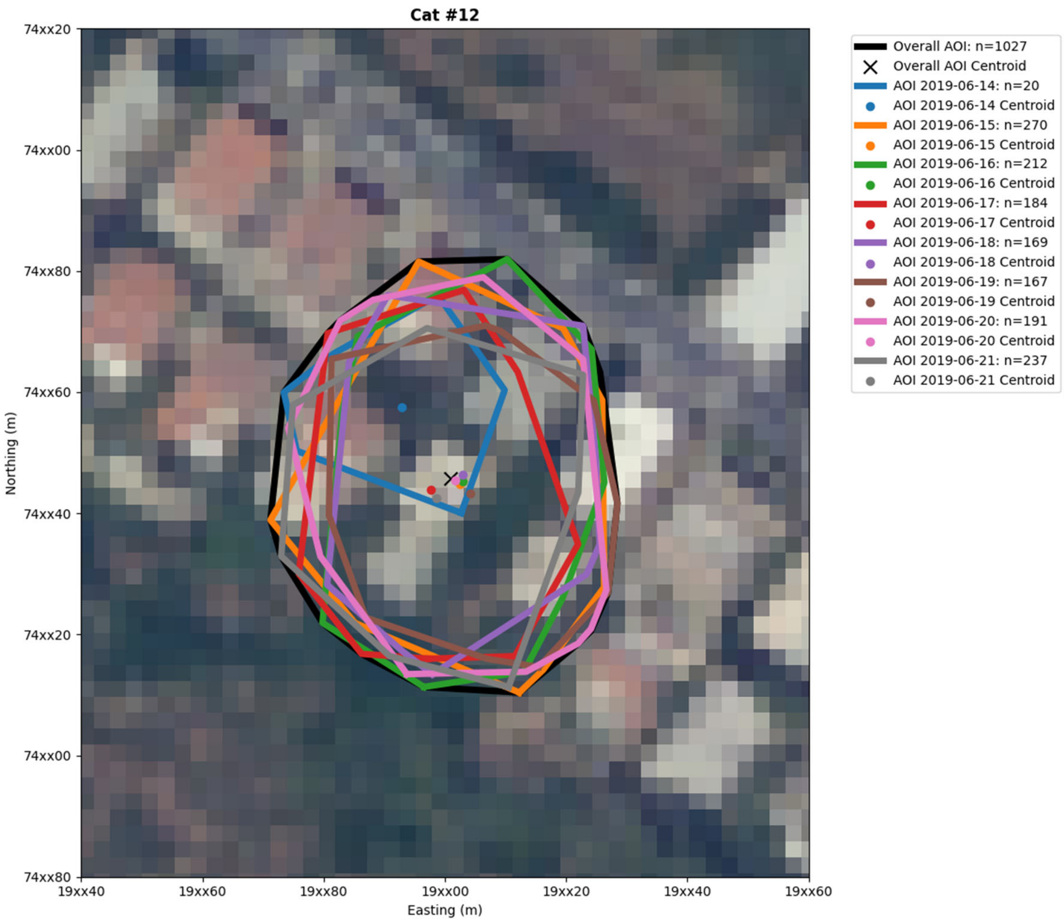
Cat #12
Overall AOI: n=1027
Overall AOI Centroid
AOI 2019-06-14: n=20
AOI 2019-06-14 Centroid
AOI 2019-06-15: n=270
AOI 2019-06-15 Centroid
AOI 2019-06-16: n=212
AOI 2019-06-16 Centroid
AOI 2019-06-17: n=184
AOI 2019-06-17 Centroid
AOI 2019-06-18: n=169
AOI 2019-06-18 Centroid
AOI 2019-06-19: n=167
AOI 2019-06-19 Centroid
AOI 2019-06-20: n=191
AOI 2019-06-20 Centroid
AOI 2019-06-21: n=237
AOI 2019-06-21 Centroid
Northing (m)
74xx20
74xx00
74xx80
74xx60
74xx40
74xx20
74xx00
74xx80
Easting (m)
19xx40
19xx60
19xx80
19xx00
19xx20
19xx40
19xx60

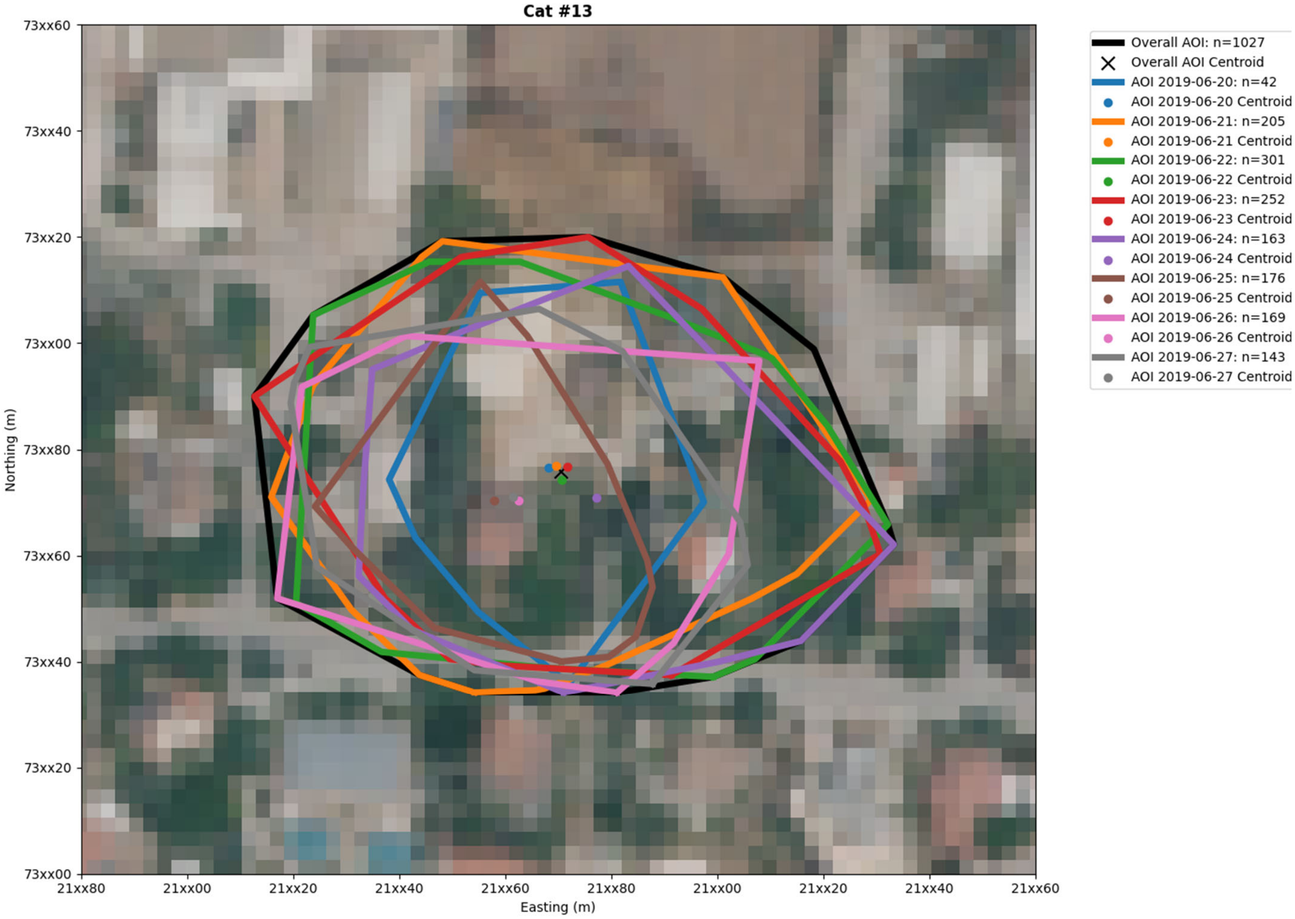
Cat #13
Overall AOI: n=1027
Overall AOI Centroid
AOI 2019-06-20: n=42
AOI 2019-06-20 Centroid
AOI 2019-06-21: n=205
AOI 2019-06-21 Centroid
AOI 2019-06-22: n=301
AOI 2019-06-22 Centroid
AOI 2019-06-23: n=252
AOI 2019-06-23 Centroid
AOI 2019-06-24: n=163
AOI 2019-06-24 Centroid
AOI 2019-06-25: n=176
AOI 2019-06-25 Centroid
AOI 2019-06-26: n=169
AOI 2019-06-26 Centroid
AOI 2019-06-27: n=143
AOI 2019-06-27 Centroid
Northing (m)
73xx60
73xx40
73xx20
73xx00
73xx80
73xx60
73xx40
73xx20
73xx00
Easting (m)
21xx80
21xx00
21xx20
21xx40
21xx60
21xx80
21xx00
21xx20
21xx40
21xx60

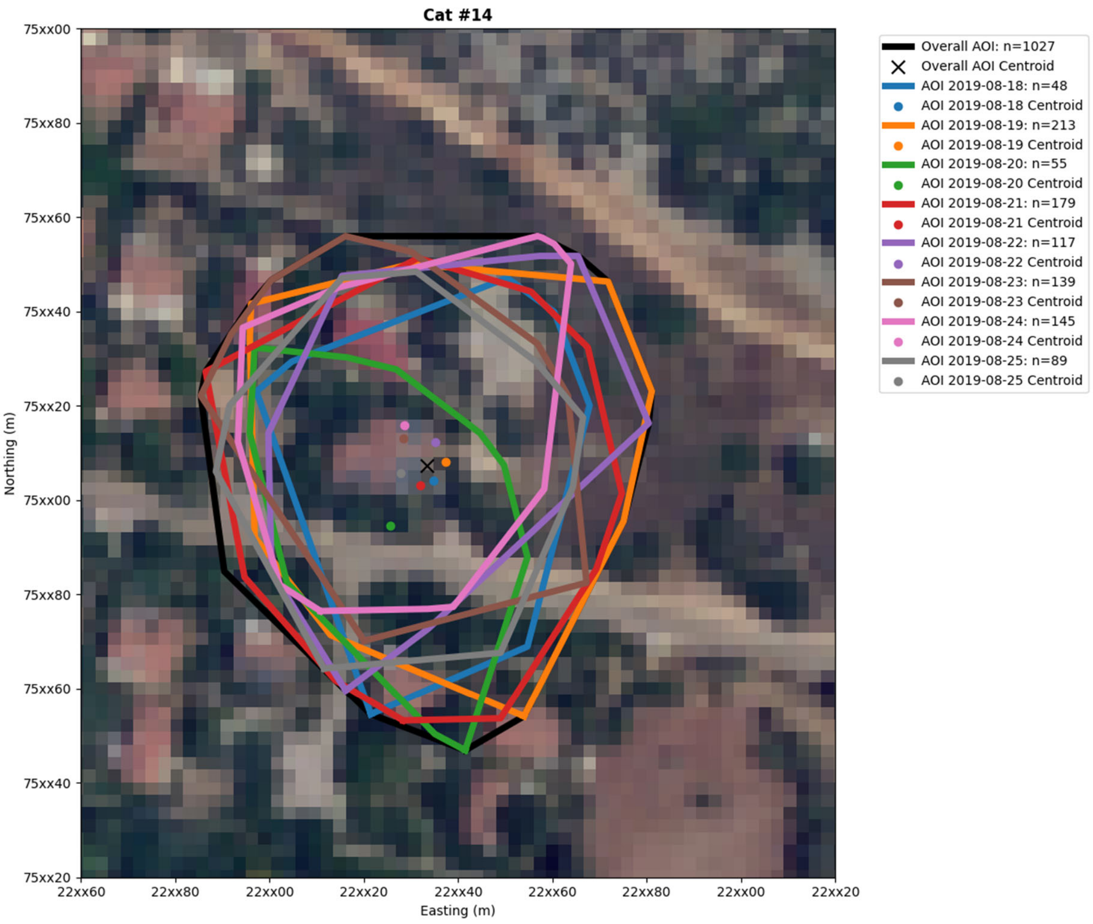
Cat #14
Overall AOI: n=1027
Overall AOI Centroid
AOI 2019-08-18: n=48
AOI 2019-08-18 Centroid
AOI 2019-08-19: n=213
AOI 2019-08-19 Centroid
AOI 2019-08-20: n=55
AOI 2019-08-20 Centroid
AOI 2019-08-21: n=179
AOI 2019-08-21 Centroid
AOI 2019-08-22: n=117
AOI 2019-08-22 Centroid
AOI 2019-08-23: n=139
AOI 2019-08-23 Centroid
AOI 2019-08-24: n=145
AOI 2019-08-24 Centroid
AOI 2019-08-25: n=89
AOI 2019-08-25 Centroid
Northing (m)
75xx00
75xx80
75xx60
75xx40
75xx20
75xx00
75xx80
75xx60
75xx40
75xx20
Easting (m)
22xx60
22xx80
22xx00
22xx20
22xx40
22xx60
22xx80
22xx00
22xx20

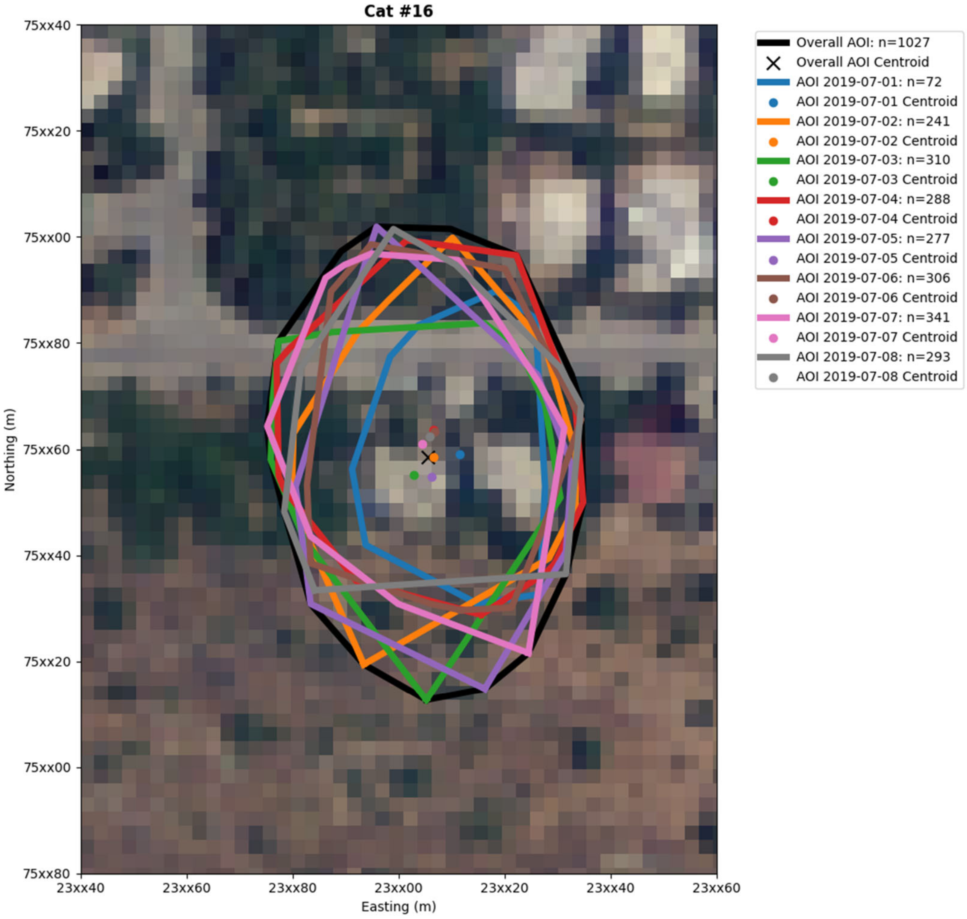
Cat #16
Overall AOI: n=1027
Overall AOI Centroid
AOI 2019-07-01: n=72
AOI 2019-07-01 Centroid
AOI 2019-07-02: n=241
AOI 2019-07-02 Centroid
AOI 2019-07-03: n=310
AOI 2019-07-03 Centroid
AOI 2019-07-04: n=288
AOI 2019-07-04 Centroid
AOI 2019-07-05: n=277
AOI 2019-07-05 Centroid
AOI 2019-07-06: n=306
AOI 2019-07-06 Centroid
AOI 2019-07-07: n=341
AOI 2019-07-07 Centroid
AOI 2019-07-08: n=293
AOI 2019-07-08 Centroid
Northing (m)
75xx40
75xx20
75xx00
75xx80
75xx60
75xx40
75xx20
75xx00
75xx80
Easting (m)
23xx40
23xx60
23xx80
23xx00
23xx20
23xx40
23xx60

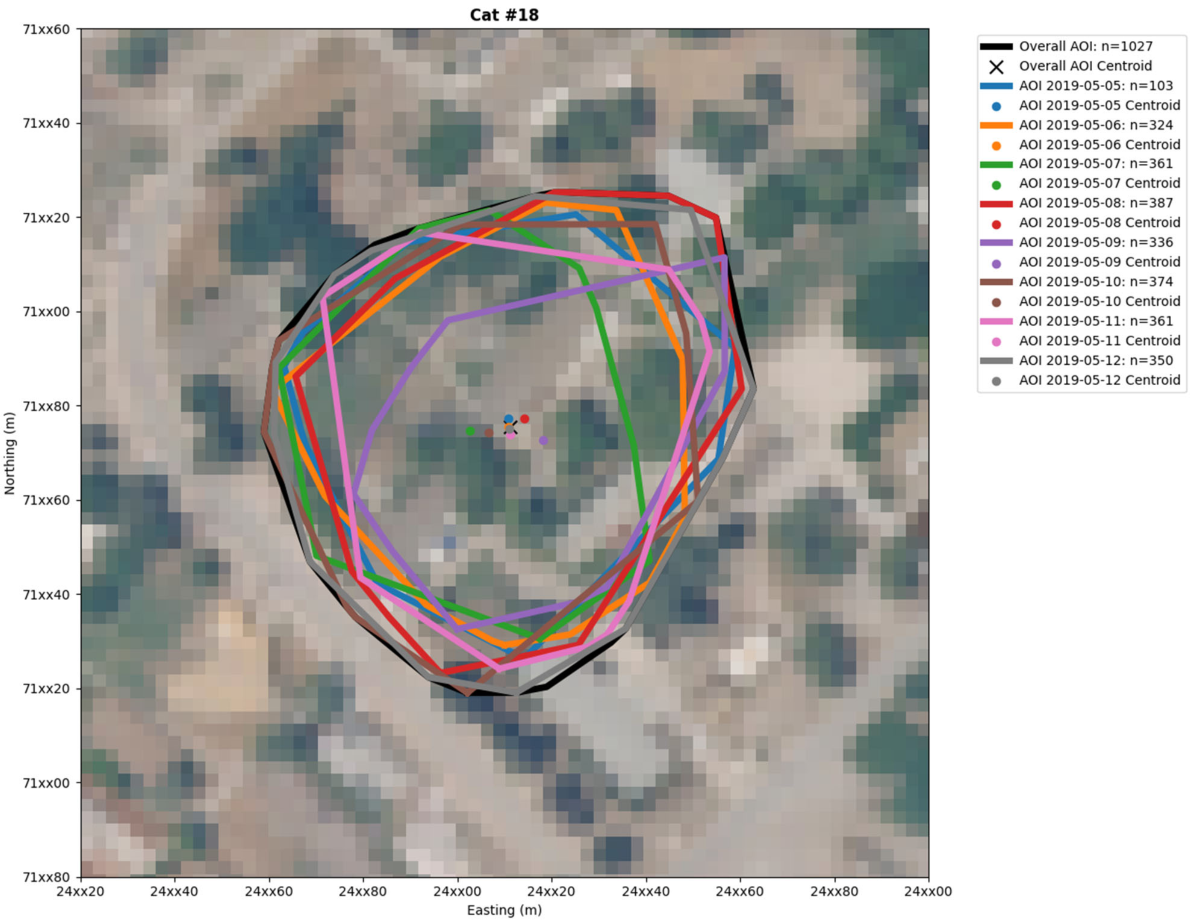
Cat #18
Overall AOI: n=1027
Overall AOI Centroid
AOI 2019-05-05: n=103
AOI 2019-05-05 Centroid
AOI 2019-05-06: n=324
AOI 2019-05-06 Centroid
AOI 2019-05-07: n=361
AOI 2019-05-07 Centroid
AOI 2019-05-08: n=387
AOI 2019-05-08 Centroid
AOI 2019-05-09: n=336
AOI 2019-05-09 Centroid
AOI 2019-05-10: n=374
AOI 2019-05-10 Centroid
AOI 2019-05-11: n=361
AOI 2019-05-11 Centroid
AOI 2019-05-12: n=350
AOI 2019-05-12 Centroid
Northing (m)
71xx60
71xx40
71xx20
71xx00
71xx80
71xx60
71xx40
71xx20
71xx00
71xx80
Easting (m)
24xx20
24xx40
24xx60
24xx80
24xx00
24xx20
24xx40
24xx60
24xx80
24xx00

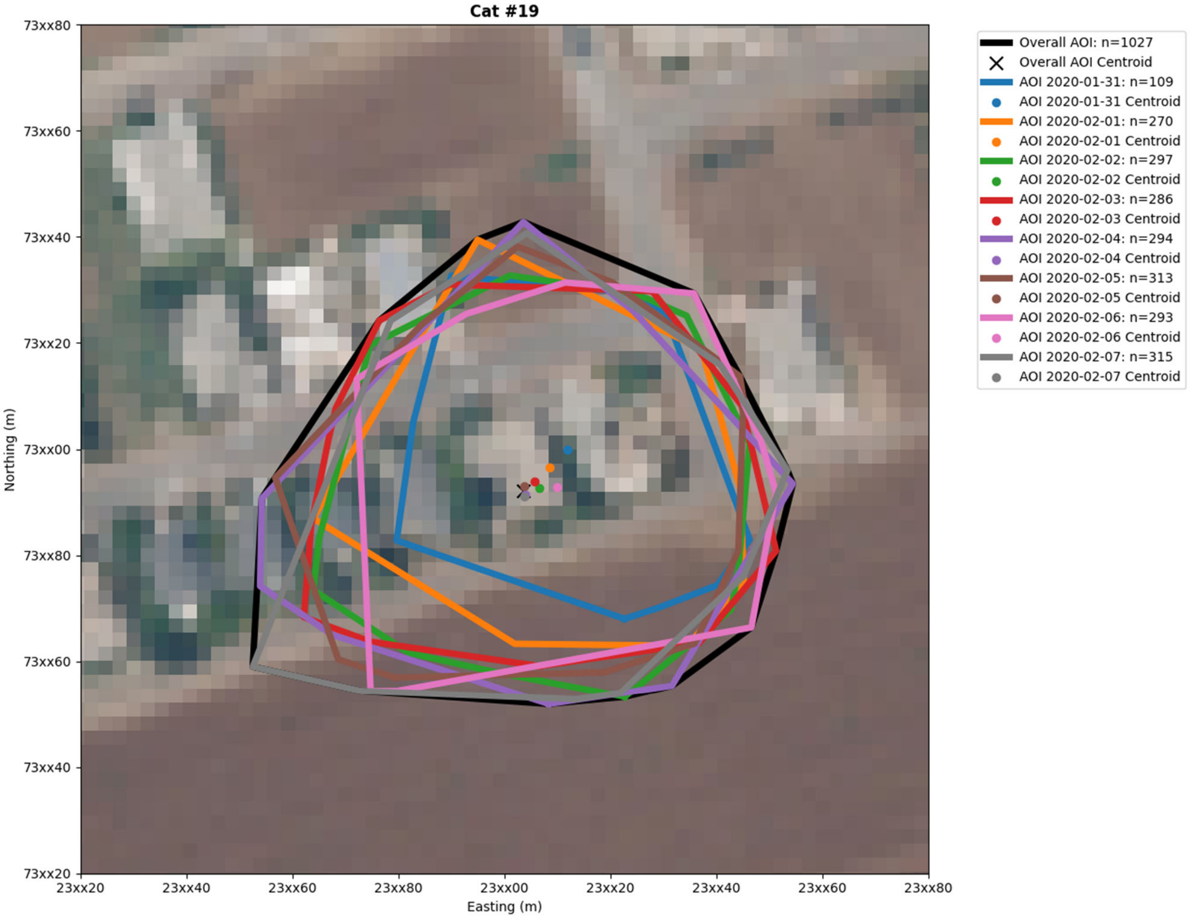
Cat #19
Overall AOI: n=1027
Overall AOI Centroid
AOI 2020-01-31: n=109
AOI 2020-01-31 Centroid
AOI 2020-02-01: n=270
AOI 2020-02-01 Centroid
AOI 2020-02-02: n=297
AOI 2020-02-02 Centroid
AOI 2020-02-03: n=286
AOI 2020-02-03 Centroid
AOI 2020-02-04: n=294
AOI 2020-02-04 Centroid
AOI 2020-02-05: n=313
AOI 2020-02-05 Centroid
AOI 2020-02-06: n=293
AOI 2020-02-06 Centroid
AOI 2020-02-07: n=315
AOI 2020-02-07 Centroid
Northing (m)
73xx80
73xx60
73xx40
73xx20
73xx00
73xx80
73xx60
73xx40
73xx20
Easting (m)
23xx20
23xx40
23xx60
23xx80
23xx00
23xx20
23xx40
23xx60
23xx80

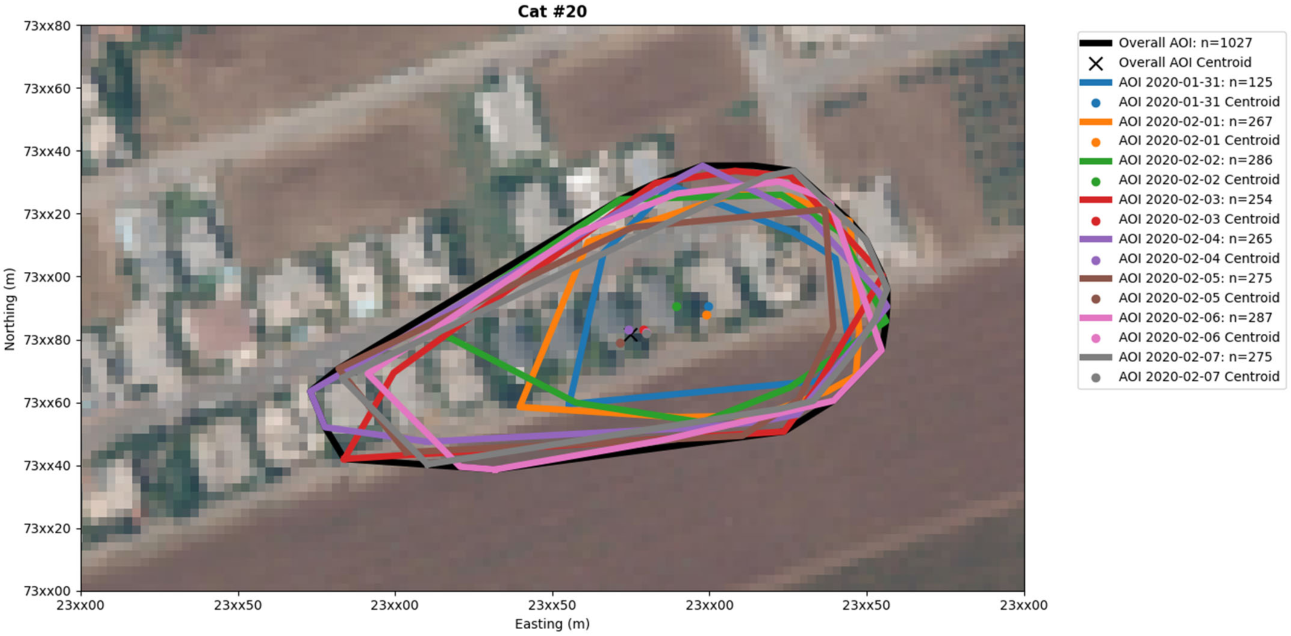
Cat #20
Northing (m)
Easting (m)
73xx80
73xx60
73xx40
73xx20
73xx00
73xx80
73xx60
73xx40
73xx20
73xx00
23xx00
23xx50
23xx00
23xx50
23xx00
23xx50
23xx00
Overall AOI: n=1027
Overall AOI Centroid
AOI 2020-01-31: n=125
AOI 2020-01-31 Centroid
AOI 2020-02-01: n=267
AOI 2020-02-01 Centroid
AOI 2020-02-02: n=286
AOI 2020-02-02 Centroid
AOI 2020-02-03: n=254
AOI 2020-02-03 Centroid
AOI 2020-02-04: n=265
AOI 2020-02-04 Centroid
AOI 2020-02-05: n=275
AOI 2020-02-05 Centroid
AOI 2020-02-06: n=287
AOI 2020-02-06 Centroid
AOI 2020-02-07: n=275
AOI 2020-02-07 Centroid

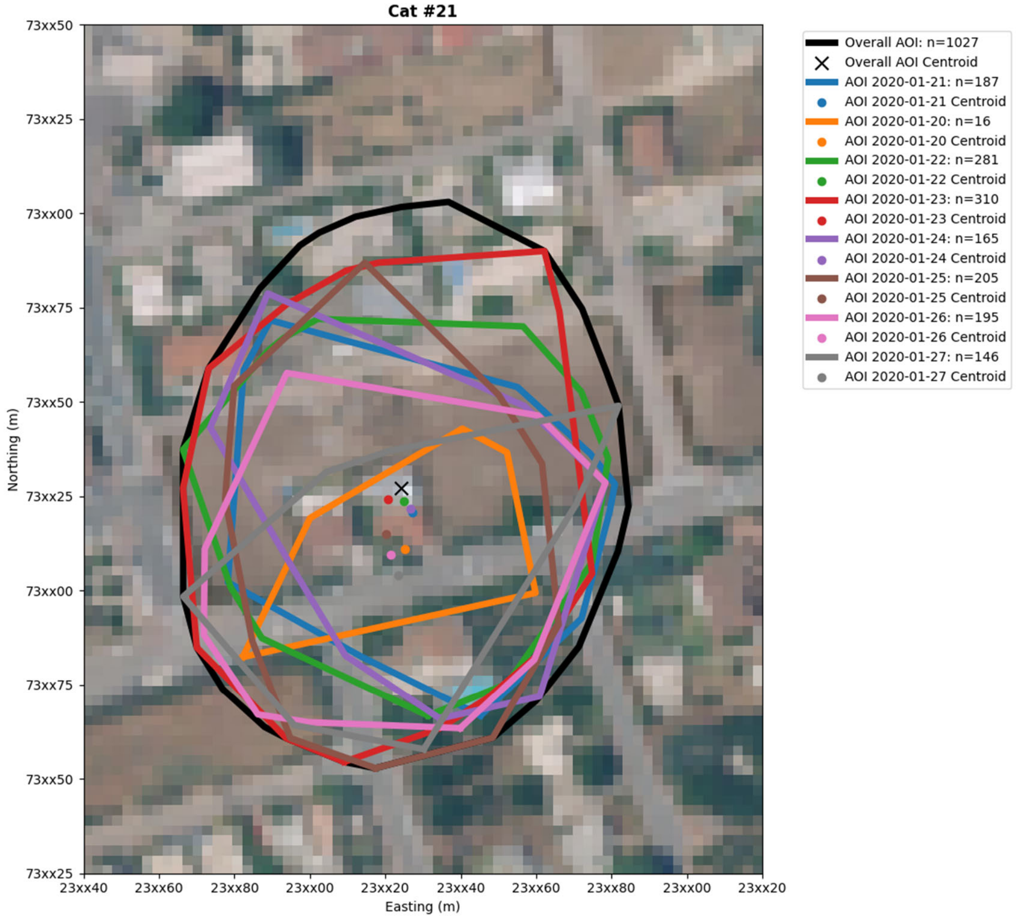
Cat #21
Northing (m)
Easting (m)
73xx50
73xx25
73xx00
73xx75
73xx50
73xx25
73xx00
73xx75
73xx50
73xx25
23xx40
23xx60
23xx80
23xx00
23xx20
23xx40
23xx60
23xx80
23xx00
23xx20
Overall AOI: n=1027
Overall AOI Centroid
AOI 2020-01-21: n=187
AOI 2020-01-21 Centroid
AOI 2020-01-20: n=16
AOI 2020-01-20 Centroid
AOI 2020-01-22: n=281
AOI 2020-01-22 Centroid
AOI 2020-01-23: n=310
AOI 2020-01-23 Centroid
AOI 2020-01-24: n=165
AOI 2020-01-24 Centroid
AOI 2020-01-25: n=205
AOI 2020-01-25 Centroid
AOI 2020-01-26: n=195
AOI 2020-01-26 Centroid
AOI 2020-01-27: n=146
AOI 2020-01-27 Centroid

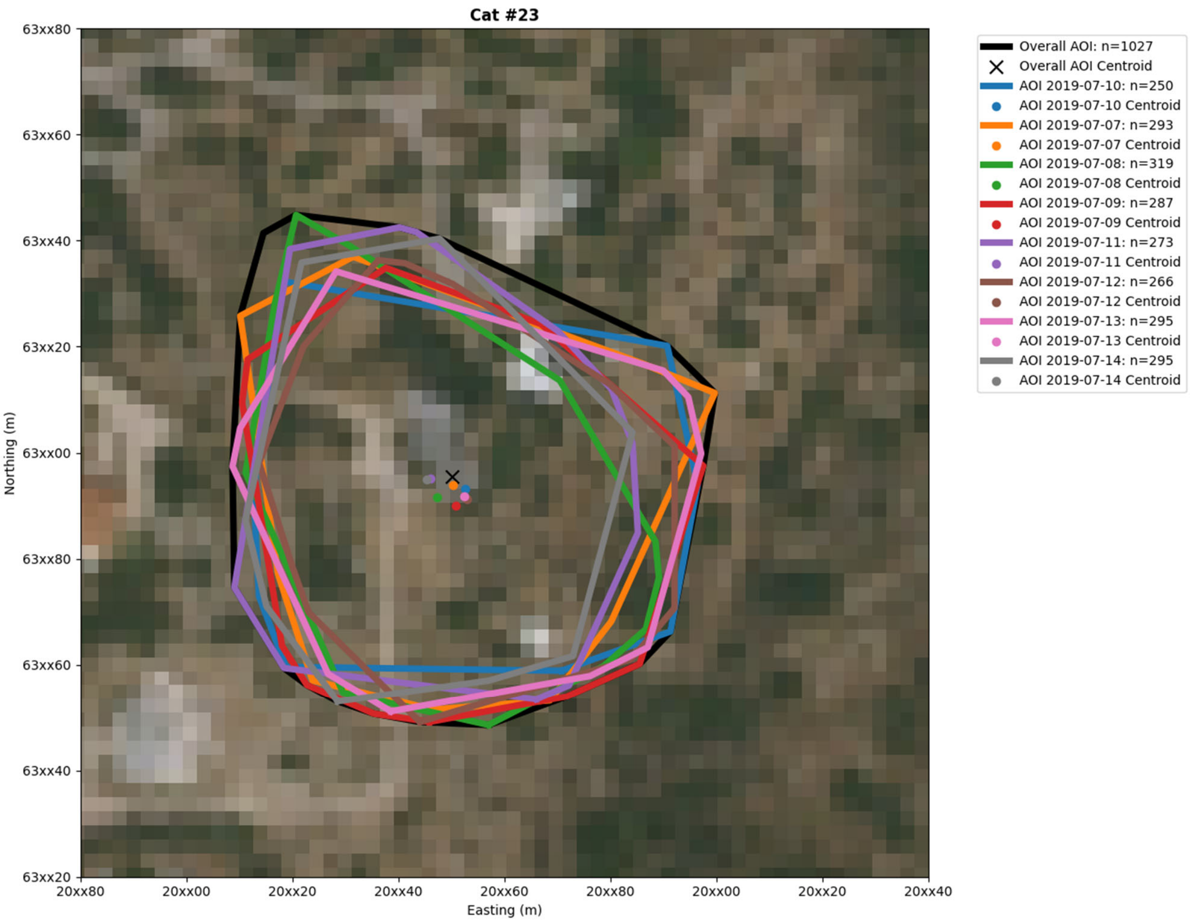

Cat #23
Northing (m)
Easting (m)
63xx80
63xx60
63xx40
63xx20
63xx00
63xx80
63xx60
63xx40
63xx20
20xx80
20xx00
20xx20
20xx40
20xx60
20xx80
20xx00
20xx20
20xx40
Overall AOI: n=1027
Overall AOI Centroid
AOI 2019-07-10: n=250
AOI 2019-07-10 Centroid
AOI 2019-07-07: n=293
AOI 2019-07-07 Centroid
AOI 2019-07-08: n=319
AOI 2019-07-08 Centroid
AOI 2019-07-09: n=287
AOI 2019-07-09 Centroid
AOI 2019-07-11: n=273
AOI 2019-07-11 Centroid
AOI 2019-07-12: n=266
AOI 2019-07-12 Centroid
AOI 2019-07-13: n=295
AOI 2019-07-13 Centroid
AOI 2019-07-14: n=295
AOI 2019-07-14 Centroid

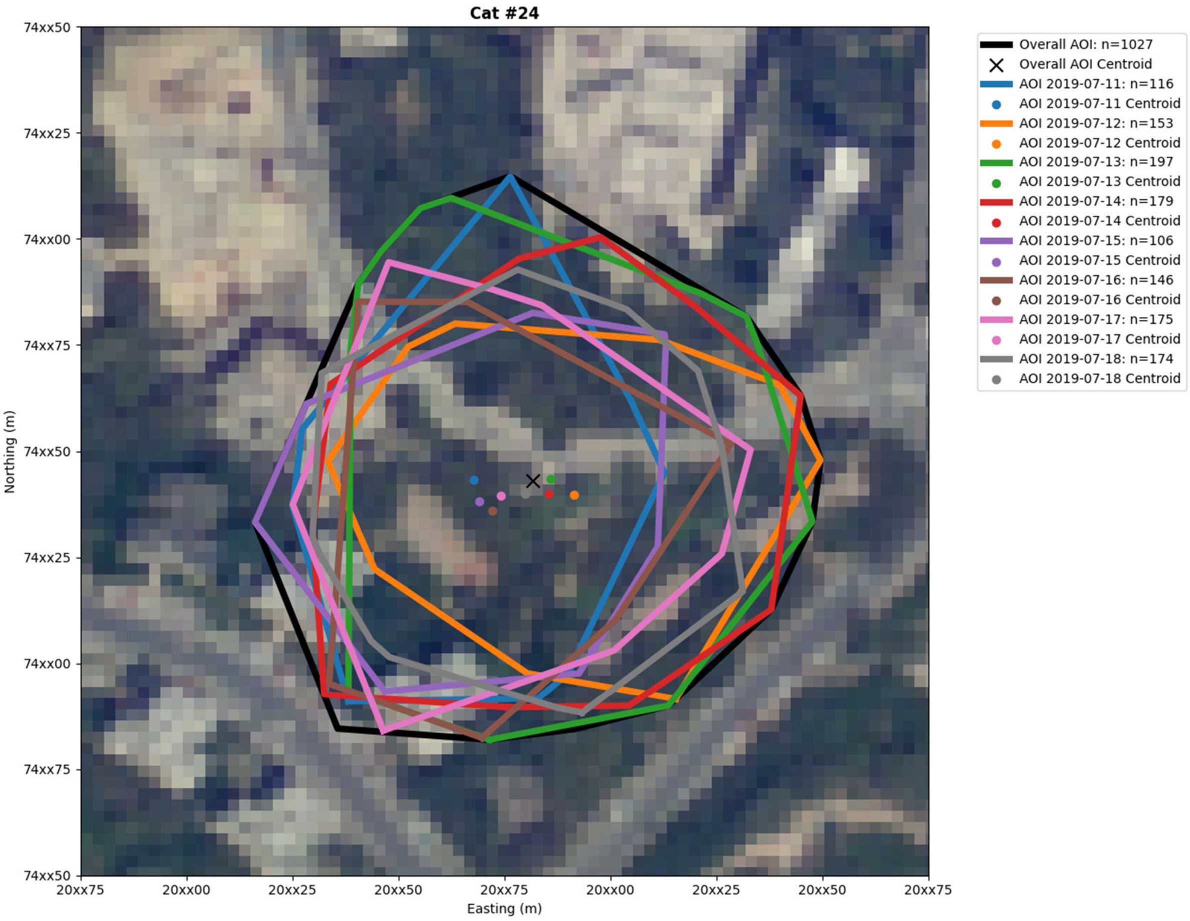

Cat #24
Northing (m)
Easting (m)
74xx50
74xx25
74xx00
74xx75
74xx50
74xx25
74xx00
74xx75
74xx50
20xx75
20xx00
20xx25
20xx50
20xx75
20xx00
20xx25
20xx50
20xx75
Overall AOI: n=1027
Overall AOI Centroid
AOI 2019-07-11: n=116
AOI 2019-07-11 Centroid
AOI 2019-07-12: n=153
AOI 2019-07-12 Centroid
AOI 2019-07-13: n=197
AOI 2019-07-13 Centroid
AOI 2019-07-14: n=179
AOI 2019-07-14 Centroid
AOI 2019-07-15: n=106
AOI 2019-07-15 Centroid
AOI 2019-07-16: n=146
AOI 2019-07-16 Centroid
AOI 2019-07-17: n=175
AOI 2019-07-17 Centroid
AOI 2019-07-18: n=174
AOI 2019-07-18 Centroid

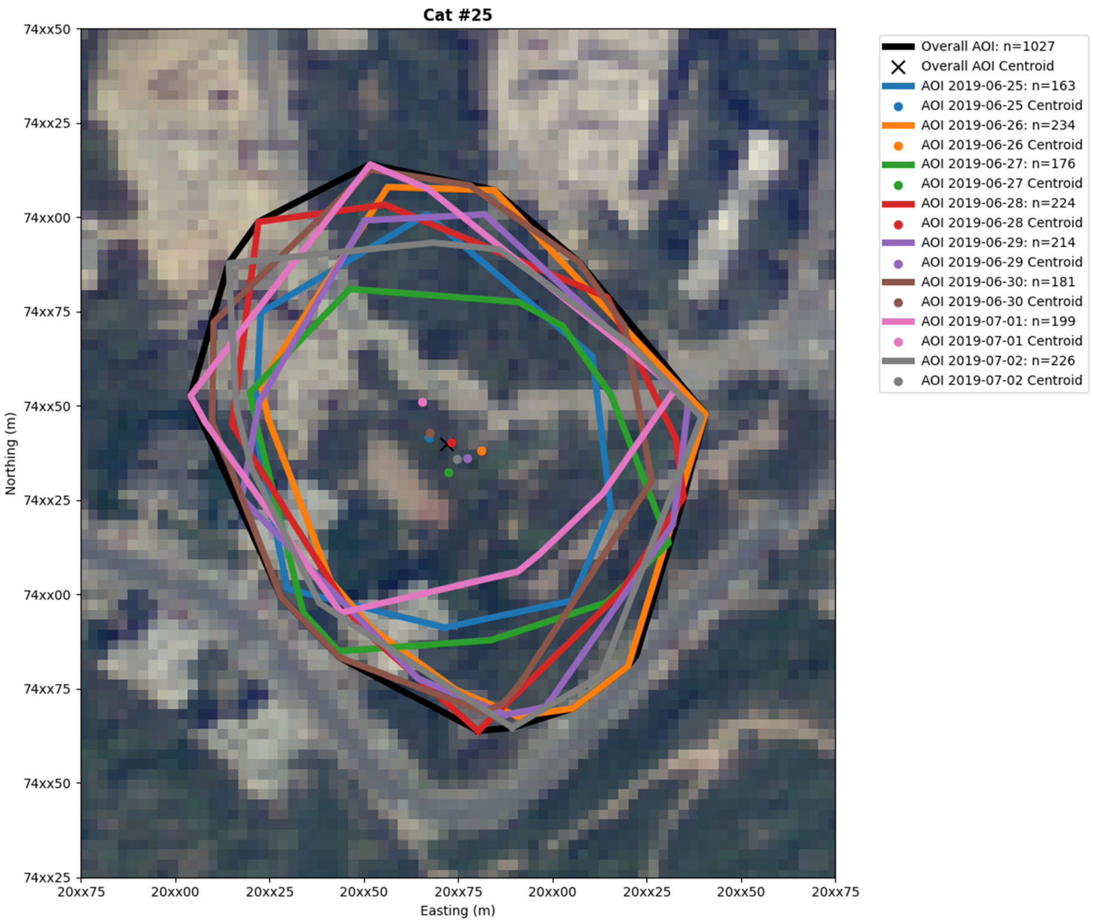
Cat #25
74xx50
74xx25
74xx00
74xx75
74xx50
74xx25
74xx00
74xx75
74xx50
74xx25
Northing (m)
20xx75
20xx00
20xx25
20xx50
20xx75
20xx00
20xx25
20xx50
20xx75
Easting (m)
Overall AOI: n=1027
Overall AOI Centroid
AOI 2019-06-25: n=163
AOI 2019-06-25 Centroid
AOI 2019-06-26: n=234
AOI 2019-06-26 Centroid
AOI 2019-06-27: n=176
AOI 2019-06-27 Centroid
AOI 2019-06-28: n=224
AOI 2019-06-28 Centroid
AOI 2019-06-29: n=214
AOI 2019-06-29 Centroid
AOI 2019-06-30: n=181
AOI 2019-06-30 Centroid
AOI 2019-07-01: n=199
AOI 2019-07-01 Centroid
AOI 2019-07-02: n=226
AOI 2019-07-02 Centroid

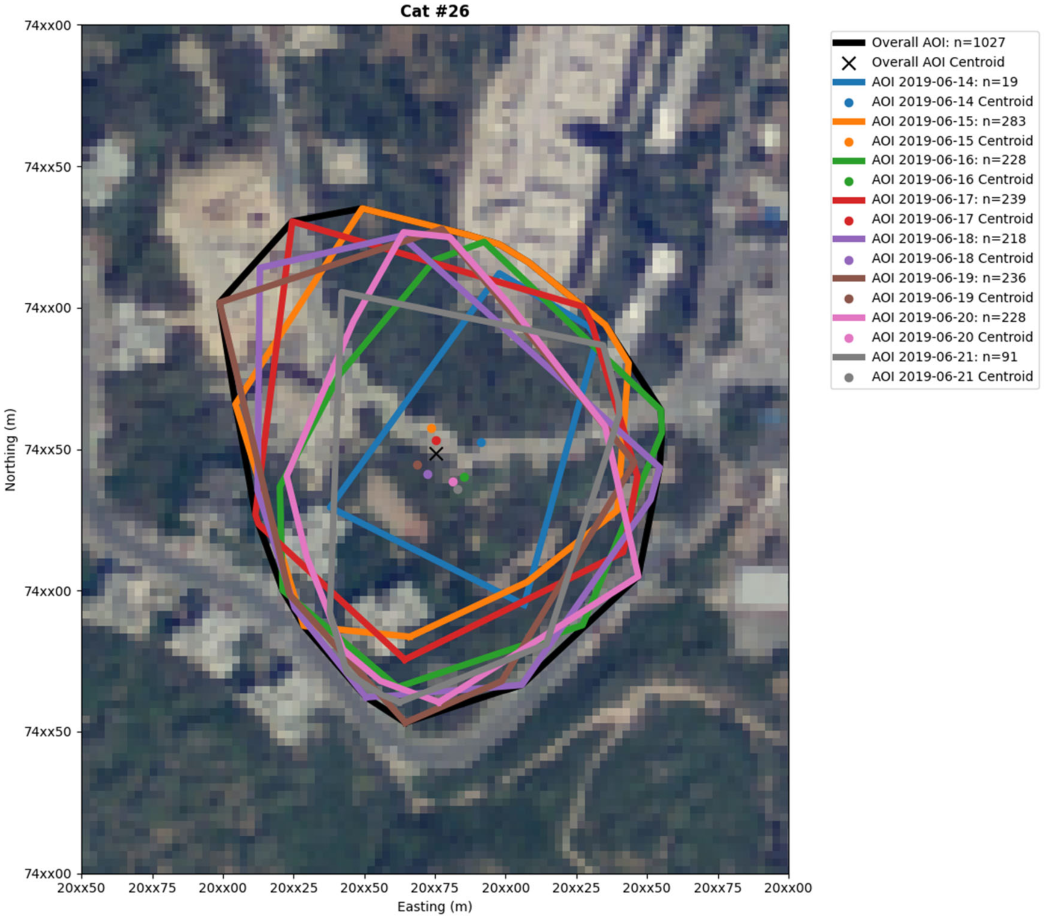
Cat #26
74xx00
74xx50
74xx00
74xx50
74xx00
74xx50
74xx00
Northing (m)
20xx50
20xx75
20xx00
20xx25
20xx50
20xx75
20xx00
20xx25
20xx50
20xx75
20xx00
Easting (m)
Overall AOI: n=1027
Overall AOI Centroid
AOI 2019-06-14: n=19
AOI 2019-06-14 Centroid
AOI 2019-06-15: n=283
AOI 2019-06-15 Centroid
AOI 2019-06-16: n=228
AOI 2019-06-16 Centroid
AOI 2019-06-17: n=239
AOI 2019-06-17 Centroid
AOI 2019-06-18: n=218
AOI 2019-06-18 Centroid
AOI 2019-06-19: n=236
AOI 2019-06-19 Centroid
AOI 2019-06-20: n=228
AOI 2019-06-20 Centroid
AOI 2019-06-21: n=91
AOI 2019-06-21 Centroid

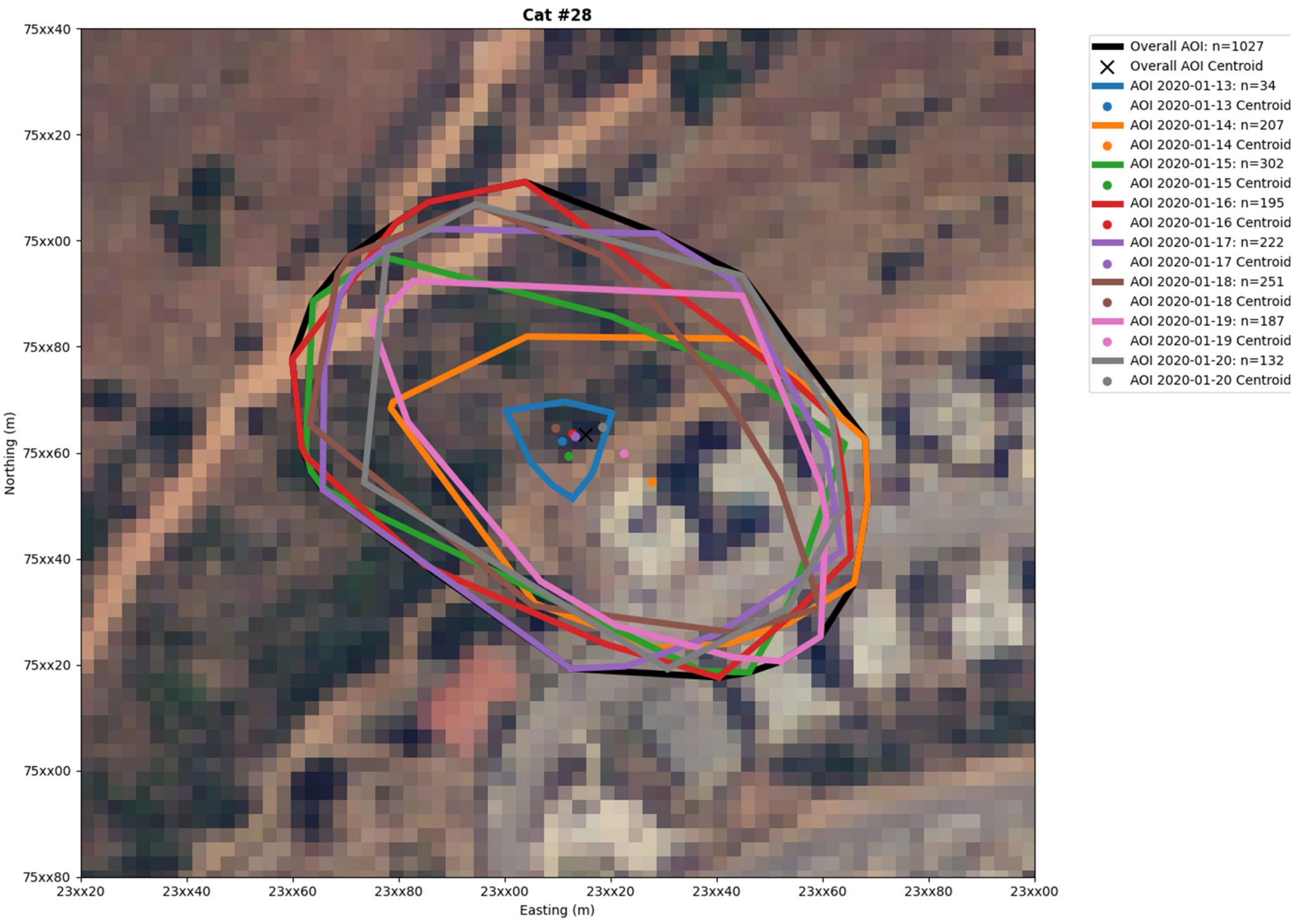
Cat #28
Overall AOI: n=1027
Overall AOI Centroid
AOI 2020-01-13: n=34
AOI 2020-01-13 Centroid
AOI 2020-01-14: n=207
AOI 2020-01-14 Centroid
AOI 2020-01-15: n=302
AOI 2020-01-15 Centroid
AOI 2020-01-16: n=195
AOI 2020-01-16 Centroid
AOI 2020-01-17: n=222
AOI 2020-01-17 Centroid
AOI 2020-01-18: n=251
AOI 2020-01-18 Centroid
AOI 2020-01-19: n=187
AOI 2020-01-19 Centroid
AOI 2020-01-20: n=132
AOI 2020-01-20 Centroid
Northing (m)
75xx40
75xx20
75xx00
75xx80
75xx60
75xx40
75xx20
75xx00
75xx80
Easting (m)
23xx20
23xx40
23xx60
23xx80
23xx00
23xx20
23xx40
23xx60
23xx80
23xx00

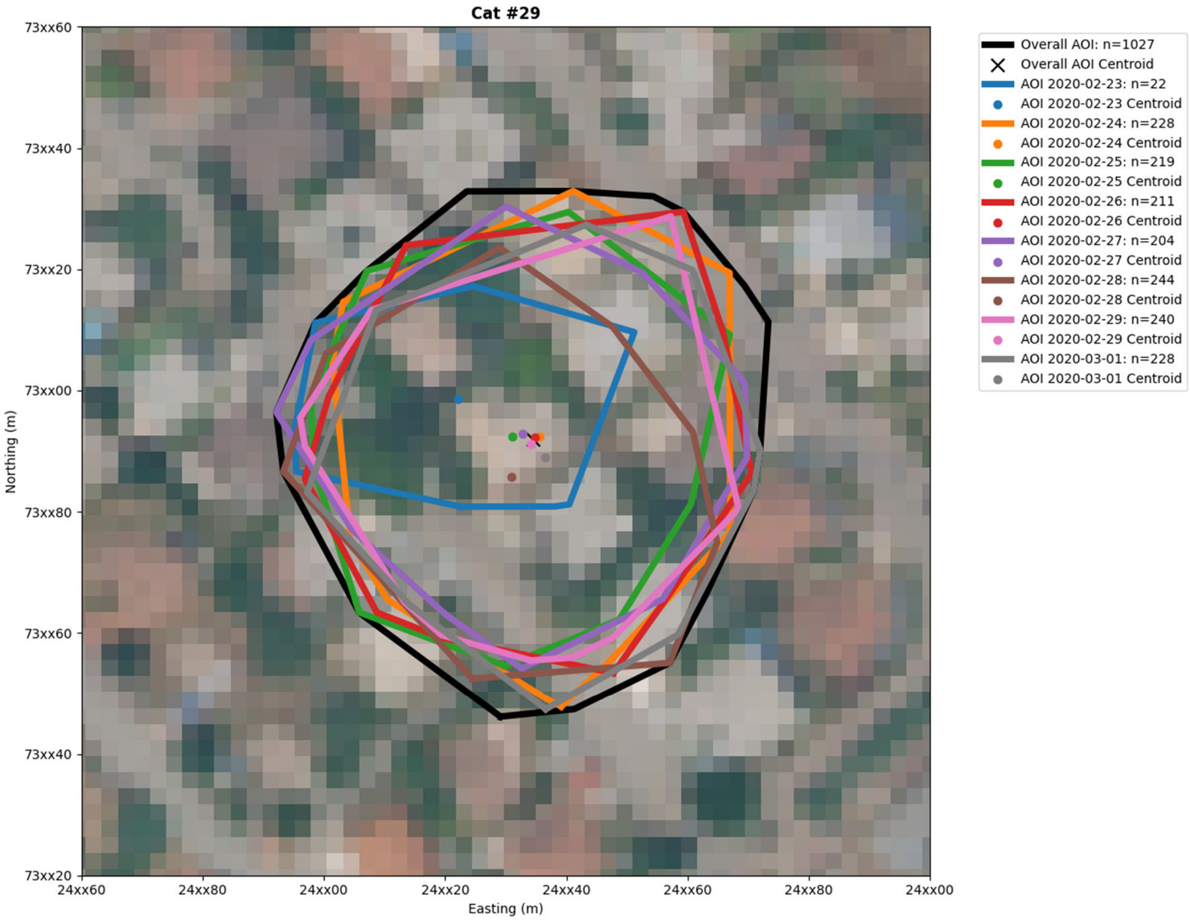
Cat #29
Overall AOI: n=1027
Overall AOI Centroid
AOI 2020-02-23: n=22
AOI 2020-02-23 Centroid
AOI 2020-02-24: n=228
AOI 2020-02-24 Centroid
AOI 2020-02-25: n=219
AOI 2020-02-25 Centroid
AOI 2020-02-26: n=211
AOI 2020-02-26 Centroid
AOI 2020-02-27: n=204
AOI 2020-02-27 Centroid
AOI 2020-02-28: n=244
AOI 2020-02-28 Centroid
AOI 2020-02-29: n=240
AOI 2020-02-29 Centroid
AOI 2020-03-01: n=228
AOI 2020-03-01 Centroid
Northing (m)
73xx60
73xx40
73xx20
73xx00
73xx80
73xx60
73xx40
73xx20
Easting (m)
24xx60
24xx80
24xx00
24xx20
24xx40
24xx60
24xx80
24xx00

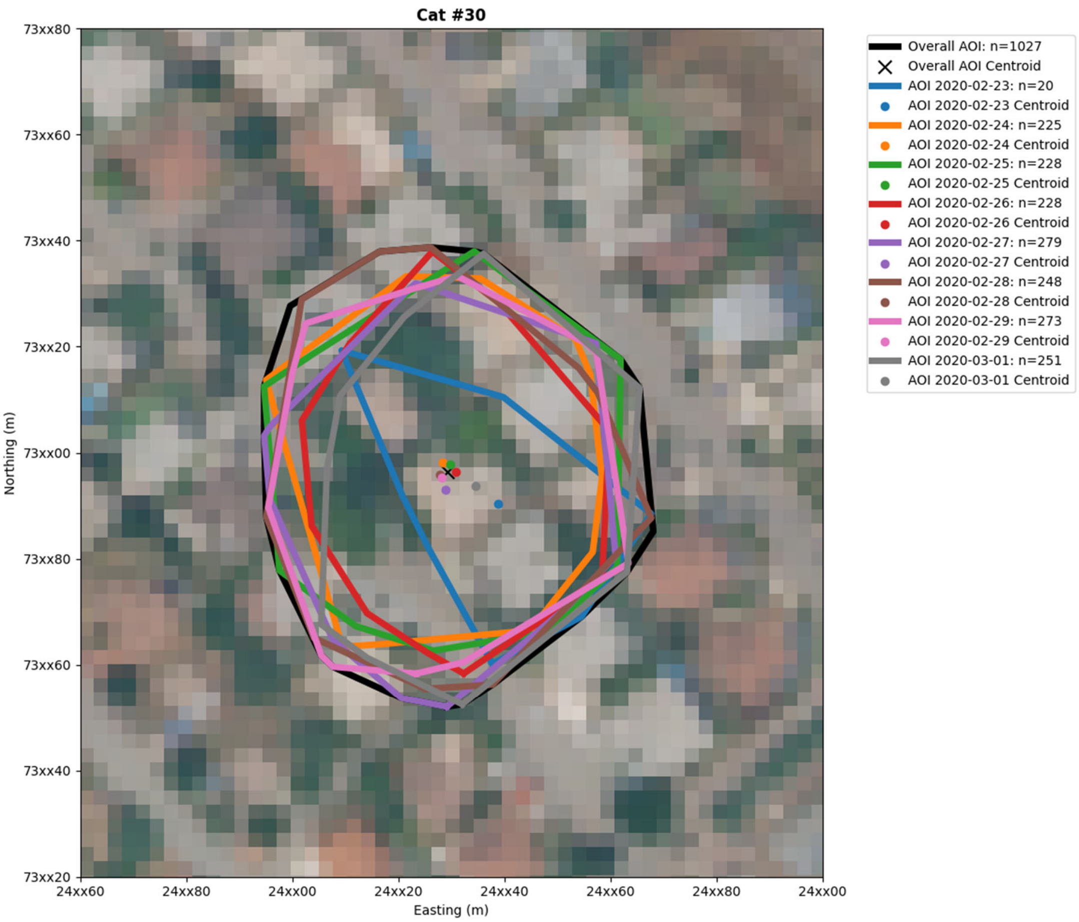
Cat #30
Overall AOI: n=1027
Overall AOI Centroid
AOI 2020-02-23: n=20
AOI 2020-02-23 Centroid
AOI 2020-02-24: n=225
AOI 2020-02-24 Centroid
AOI 2020-02-25: n=228
AOI 2020-02-25 Centroid
AOI 2020-02-26: n=228
AOI 2020-02-26 Centroid
AOI 2020-02-27: n=279
AOI 2020-02-27 Centroid
AOI 2020-02-28: n=248
AOI 2020-02-28 Centroid
AOI 2020-02-29: n=273
AOI 2020-02-29 Centroid
AOI 2020-03-01: n=251
AOI 2020-03-01 Centroid
Northing (m)
73xx80
73xx60
73xx40
73xx20
73xx00
73xx80
73xx60
73xx40
73xx20
Easting (m)
24xx60
24xx80
24xx00
24xx20
24xx40
24xx60
24xx80
24xx00

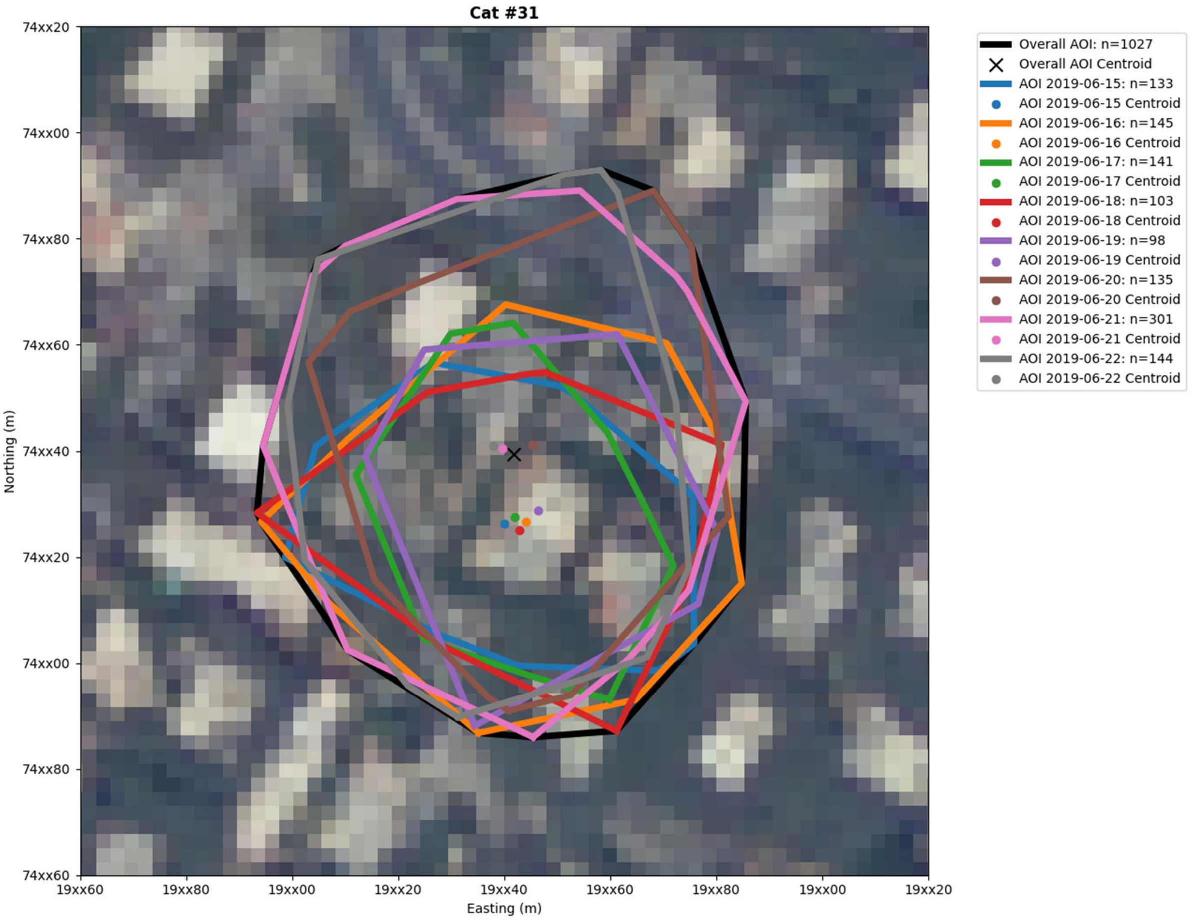
Cat #31
Overall AOI: n=1027
Overall AOI Centroid
AOI 2019-06-15: n=133
AOI 2019-06-15 Centroid
AOI 2019-06-16: n=145
AOI 2019-06-16 Centroid
AOI 2019-06-17: n=141
AOI 2019-06-17 Centroid
AOI 2019-06-18: n=103
AOI 2019-06-18 Centroid
AOI 2019-06-19: n=98
AOI 2019-06-19 Centroid
AOI 2019-06-20: n=135
AOI 2019-06-20 Centroid
AOI 2019-06-21: n=301
AOI 2019-06-21 Centroid
AOI 2019-06-22: n=144
AOI 2019-06-22 Centroid
Northing (m)
74xx20
74xx00
74xx80
74xx60
74xx40
74xx20
74xx00
74xx80
74xx60
Easting (m)
19xx60
19xx80
19xx00
19xx20
19xx40
19xx60
19xx80
19xx00
19xx20

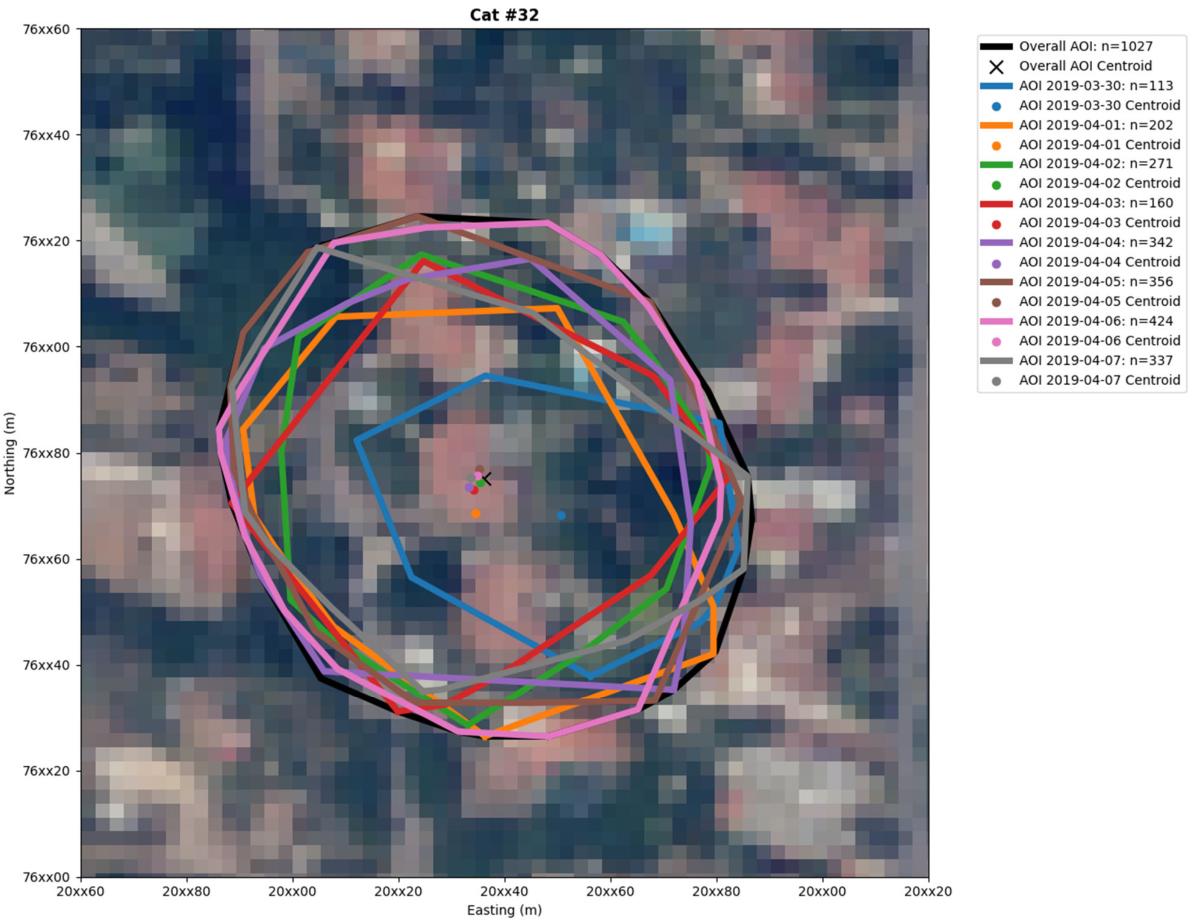
Cat #32
Overall AOI: n=1027
Overall AOI Centroid
AOI 2019-03-30: n=113
AOI 2019-03-30 Centroid
AOI 2019-04-01: n=202
AOI 2019-04-01 Centroid
AOI 2019-04-02: n=271
AOI 2019-04-02 Centroid
AOI 2019-04-03: n=160
AOI 2019-04-03 Centroid
AOI 2019-04-04: n=342
AOI 2019-04-04 Centroid
AOI 2019-04-05: n=356
AOI 2019-04-05 Centroid
AOI 2019-04-06: n=424
AOI 2019-04-06 Centroid
AOI 2019-04-07: n=337
AOI 2019-04-07 Centroid
Northing (m)
Easting (m)
76xx60
76xx40
76xx20
76xx00
76xx80
76xx60
76xx40
76xx20
76xx00
20xx60
20xx80
20xx00
20xx20
20xx40
20xx60
20xx80
20xx00
20xx20

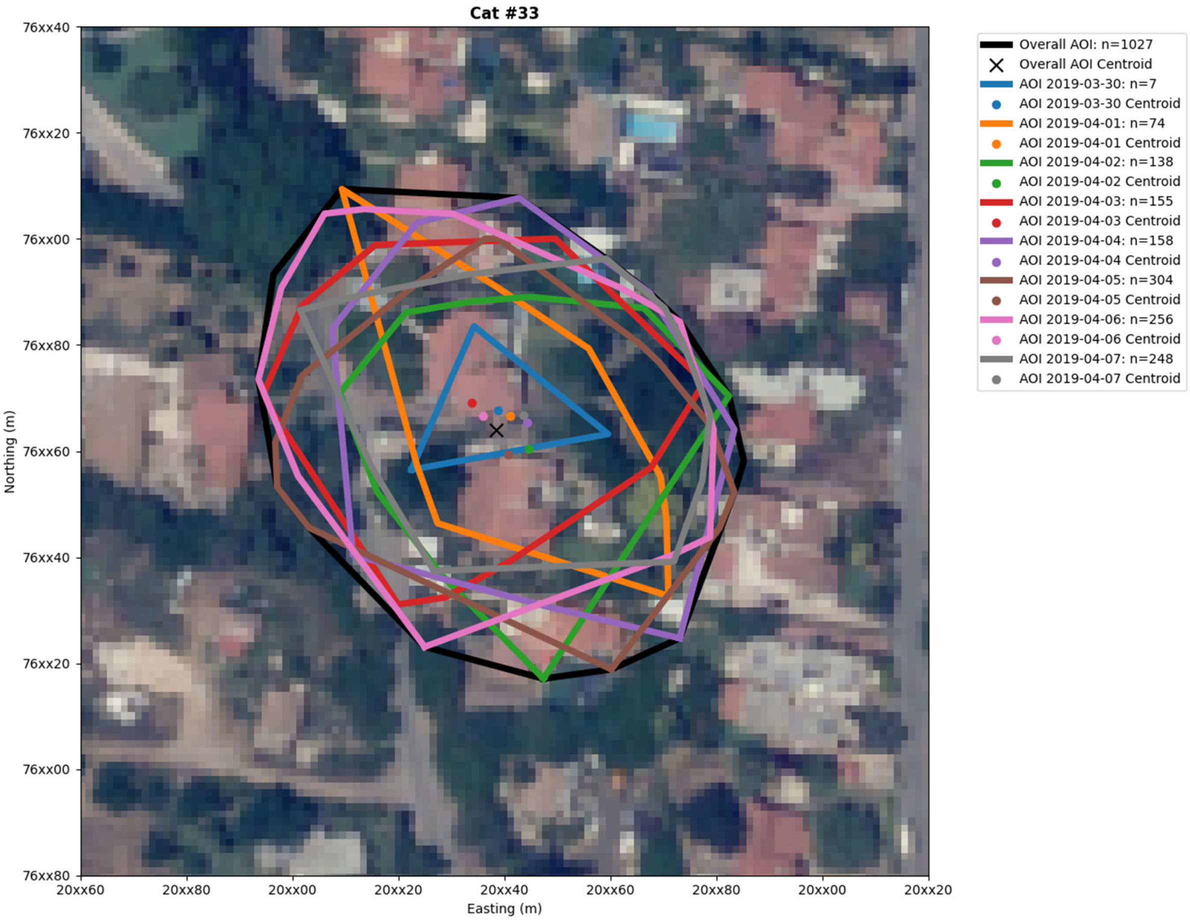
Cat #33
Overall AOI: n=1027
Overall AOI Centroid
AOI 2019-03-30: n=7
AOI 2019-03-30 Centroid
AOI 2019-04-01: n=74
AOI 2019-04-01 Centroid
AOI 2019-04-02: n=138
AOI 2019-04-02 Centroid
AOI 2019-04-03: n=155
AOI 2019-04-03 Centroid
AOI 2019-04-04: n=158
AOI 2019-04-04 Centroid
AOI 2019-04-05: n=304
AOI 2019-04-05 Centroid
AOI 2019-04-06: n=256
AOI 2019-04-06 Centroid
AOI 2019-04-07: n=248
AOI 2019-04-07 Centroid
Northing (m)
Easting (m)
76xx40
76xx20
76xx00
76xx80
76xx60
76xx40
76xx20
76xx00
76xx80
20xx60
20xx80
20xx00
20xx20
20xx40
20xx60
20xx80
20xx00
20xx20

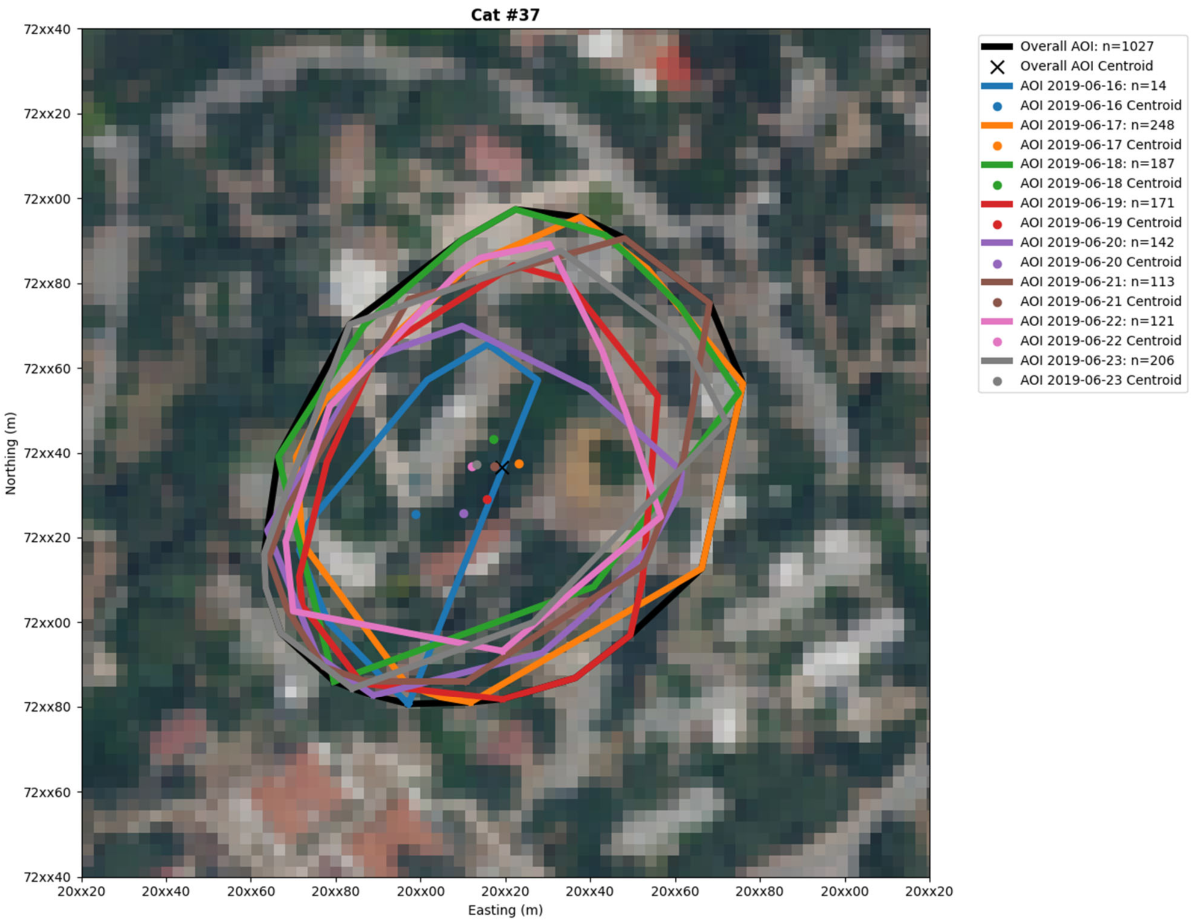
Cat #37
Northing (m)
72xx40
72xx20
72xx00
72xx80
72xx60
72xx40
72xx20
72xx00
72xx80
72xx60
72xx40
Easting (m)
20xx20
20xx40
20xx60
20xx80
20xx00
20xx20
20xx40
20xx60
20xx80
20xx00
20xx20
Overall AOI: n=1027
Overall AOI Centroid
AOI 2019-06-16: n=14
AOI 2019-06-16 Centroid
AOI 2019-06-17: n=248
AOI 2019-06-17 Centroid
AOI 2019-06-18: n=187
AOI 2019-06-18 Centroid
AOI 2019-06-19: n=171
AOI 2019-06-19 Centroid
AOI 2019-06-20: n=142
AOI 2019-06-20 Centroid
AOI 2019-06-21: n=113
AOI 2019-06-21 Centroid
AOI 2019-06-22: n=121
AOI 2019-06-22 Centroid
AOI 2019-06-23: n=206
AOI 2019-06-23 Centroid

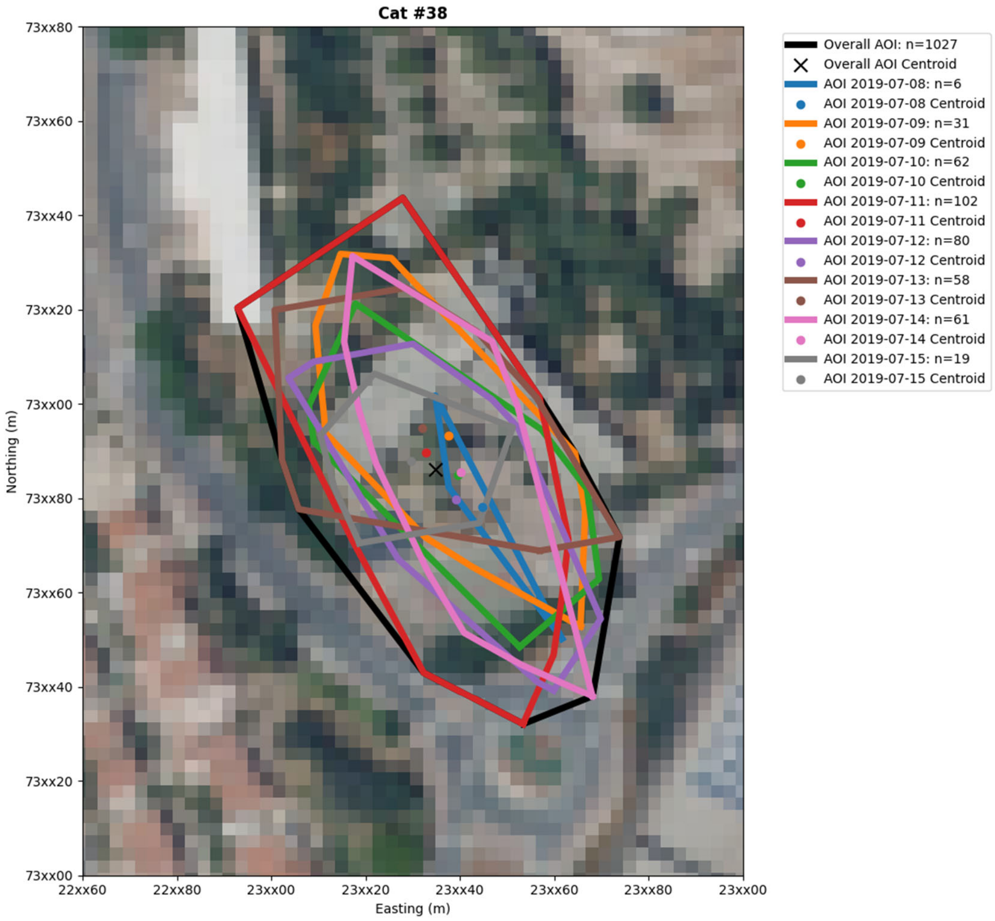
Cat #38
Northing (m)
73xx80
73xx60
73xx40
73xx20
73xx00
73xx80
73xx60
73xx40
73xx20
73xx00
Easting (m)
22xx60
22xx80
23xx00
23xx20
23xx40
23xx60
23xx80
23xx00
Overall AOI: n=1027
Overall AOI Centroid
AOI 2019-07-08: n=6
AOI 2019-07-08 Centroid
AOI 2019-07-09: n=31
AOI 2019-07-09 Centroid
AOI 2019-07-10: n=62
AOI 2019-07-10 Centroid
AOI 2019-07-11: n=102
AOI 2019-07-11 Centroid
AOI 2019-07-12: n=80
AOI 2019-07-12 Centroid
AOI 2019-07-13: n=58
AOI 2019-07-13 Centroid
AOI 2019-07-14: n=61
AOI 2019-07-14 Centroid
AOI 2019-07-15: n=19
AOI 2019-07-15 Centroid

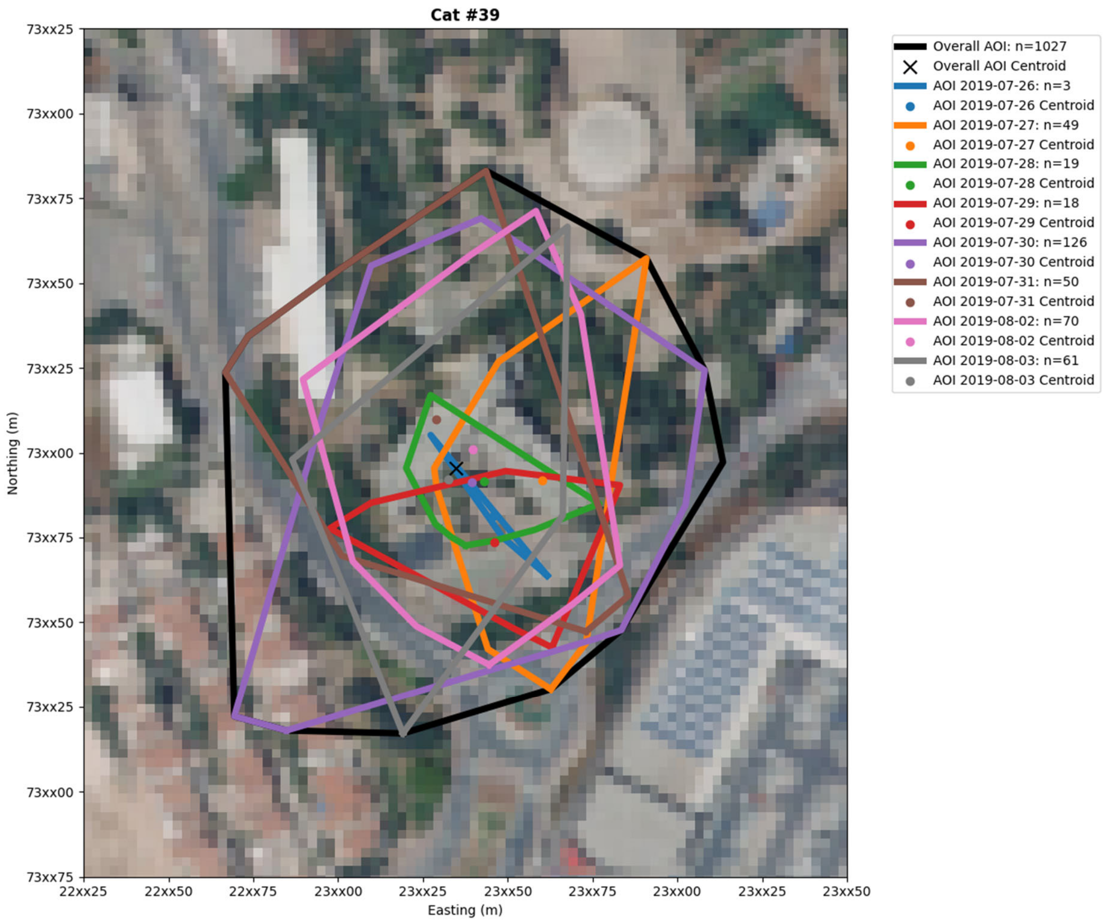
Cat #39
Overall AOI: n=1027
Overall AOI Centroid
AOI 2019-07-26: n=3
AOI 2019-07-26 Centroid
AOI 2019-07-27: n=49
AOI 2019-07-27 Centroid
AOI 2019-07-28: n=19
AOI 2019-07-28 Centroid
AOI 2019-07-29: n=18
AOI 2019-07-29 Centroid
AOI 2019-07-30: n=126
AOI 2019-07-30 Centroid
AOI 2019-07-31: n=50
AOI 2019-07-31 Centroid
AOI 2019-08-02: n=70
AOI 2019-08-02 Centroid
AOI 2019-08-03: n=61
AOI 2019-08-03 Centroid
Northing (m)
73xx25
73xx00
73xx75
73xx50
73xx25
73xx00
73xx75
73xx50
73xx25
73xx00
73xx75
Easting (m)
22xx25
22xx50
22xx75
23xx00
23xx25
23xx50
23xx75
23xx00
23xx25
23xx50

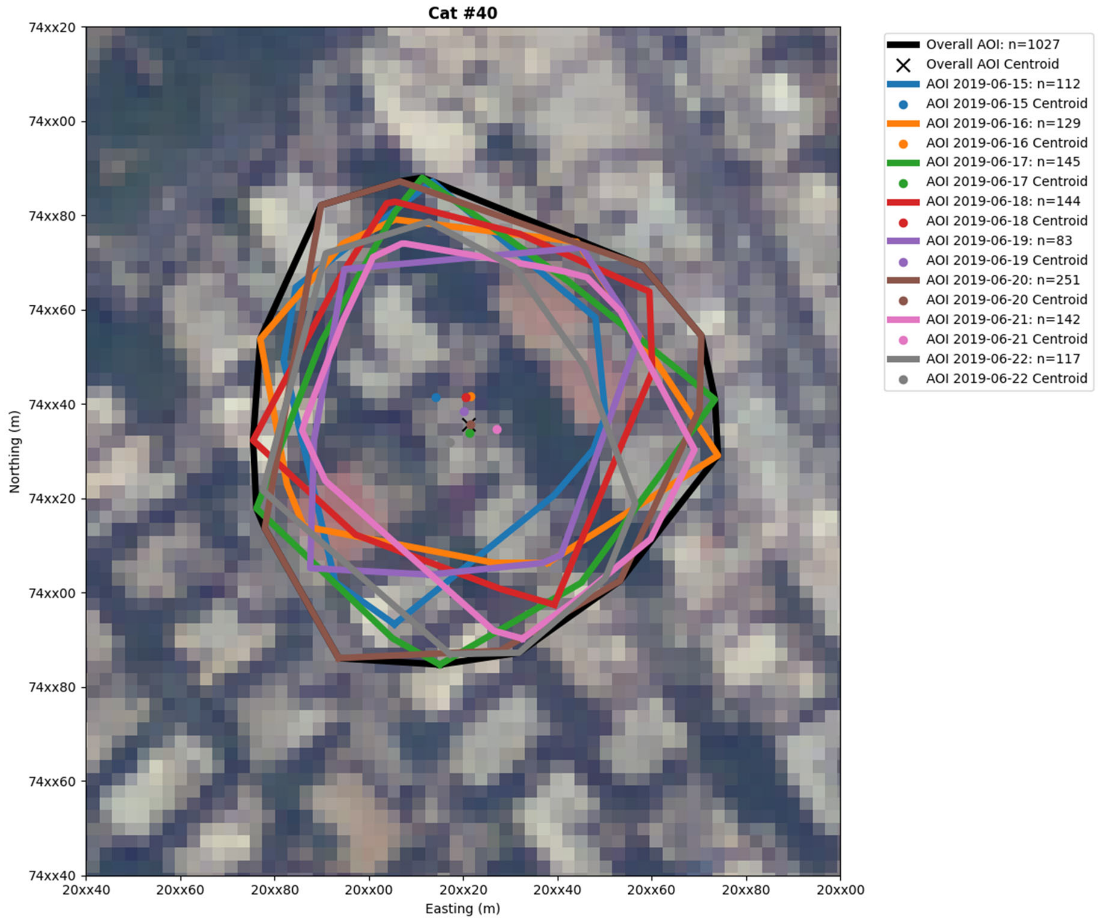
Cat #40
Overall AOI: n=1027
Overall AOI Centroid
AOI 2019-06-15: n=112
AOI 2019-06-15 Centroid
AOI 2019-06-16: n=129
AOI 2019-06-16 Centroid
AOI 2019-06-17: n=145
AOI 2019-06-17 Centroid
AOI 2019-06-18: n=144
AOI 2019-06-18 Centroid
AOI 2019-06-19: n=83
AOI 2019-06-19 Centroid
AOI 2019-06-20: n=251
AOI 2019-06-20 Centroid
AOI 2019-06-21: n=142
AOI 2019-06-21 Centroid
AOI 2019-06-22: n=117
AOI 2019-06-22 Centroid
Northing (m)
74xx20
74xx00
74xx80
74xx60
74xx40
74xx20
74xx00
74xx80
74xx60
74xx40
Easting (m)
20xx40
20xx60
20xx80
20xx00
20xx20
20xx40
20xx60
20xx80
20xx00

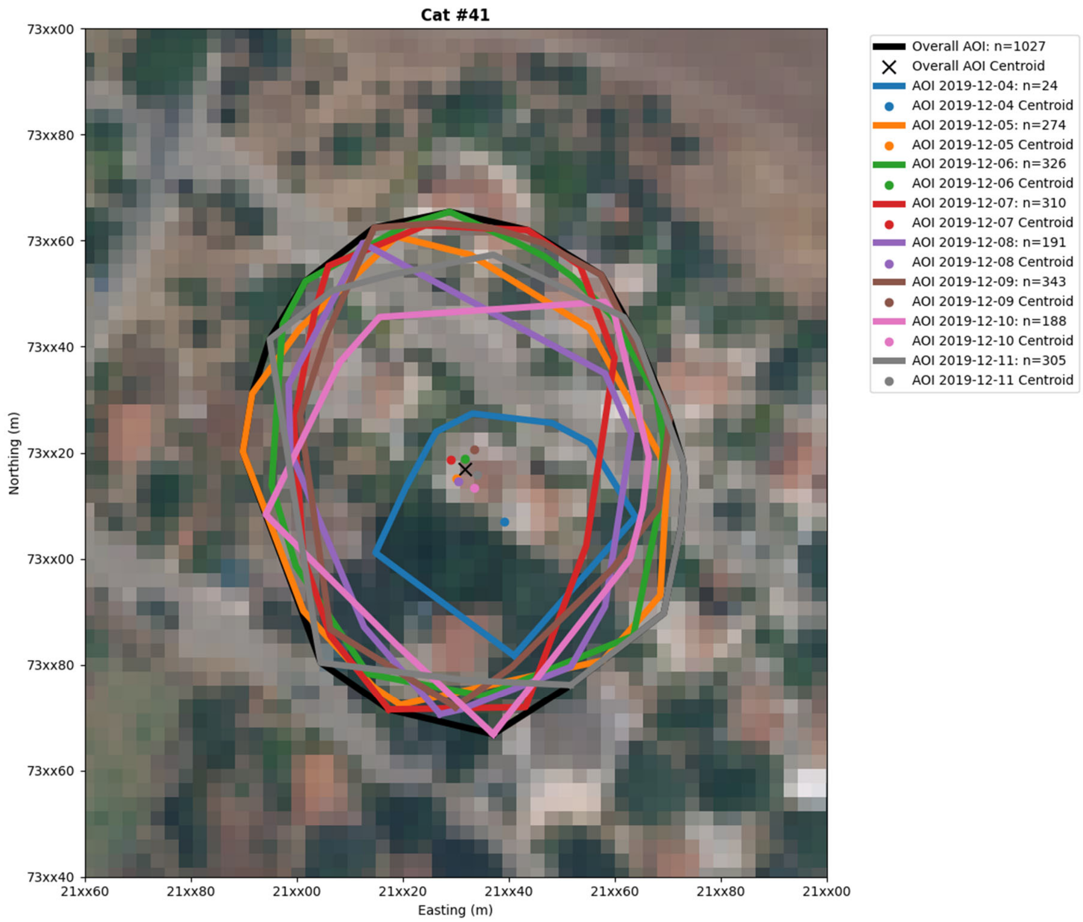
Cat #41
73xx00
73xx80
73xx60
73xx40
73xx20
73xx00
73xx80
73xx60
73xx40
Northing (m)
21xx60
21xx80
21xx00
21xx20
21xx40
21xx60
21xx80
21xx00
Easting (m)
Overall AOI: n=1027
Overall AOI Centroid
AOI 2019-12-04: n=24
AOI 2019-12-04 Centroid
AOI 2019-12-05: n=274
AOI 2019-12-05 Centroid
AOI 2019-12-06: n=326
AOI 2019-12-06 Centroid
AOI 2019-12-07: n=310
AOI 2019-12-07 Centroid
AOI 2019-12-08: n=191
AOI 2019-12-08 Centroid
AOI 2019-12-09: n=343
AOI 2019-12-09 Centroid
AOI 2019-12-10: n=188
AOI 2019-12-10 Centroid
AOI 2019-12-11: n=305
AOI 2019-12-11 Centroid

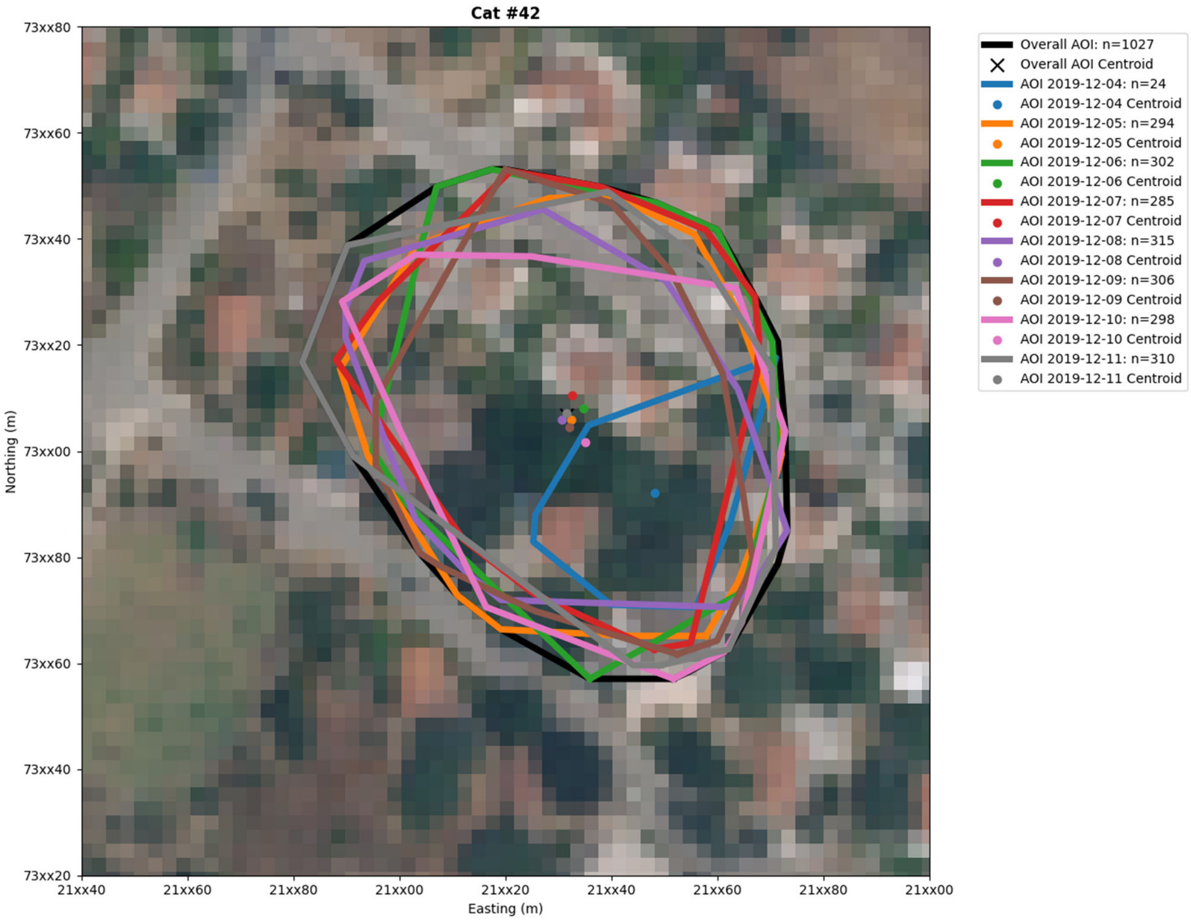
Cat #42
73xx80
73xx60
73xx40
73xx20
73xx00
73xx80
73xx60
73xx40
73xx20
Northing (m)
21xx40
21xx60
21xx80
21xx00
21xx20
21xx40
21xx60
21xx80
21xx00
Easting (m)
Overall AOI: n=1027
Overall AOI Centroid
AOI 2019-12-04: n=24
AOI 2019-12-04 Centroid
AOI 2019-12-05: n=294
AOI 2019-12-05 Centroid
AOI 2019-12-06: n=302
AOI 2019-12-06 Centroid
AOI 2019-12-07: n=285
AOI 2019-12-07 Centroid
AOI 2019-12-08: n=315
AOI 2019-12-08 Centroid
AOI 2019-12-09: n=306
AOI 2019-12-09 Centroid
AOI 2019-12-10: n=298
AOI 2019-12-10 Centroid
AOI 2019-12-11: n=310
AOI 2019-12-11 Centroid

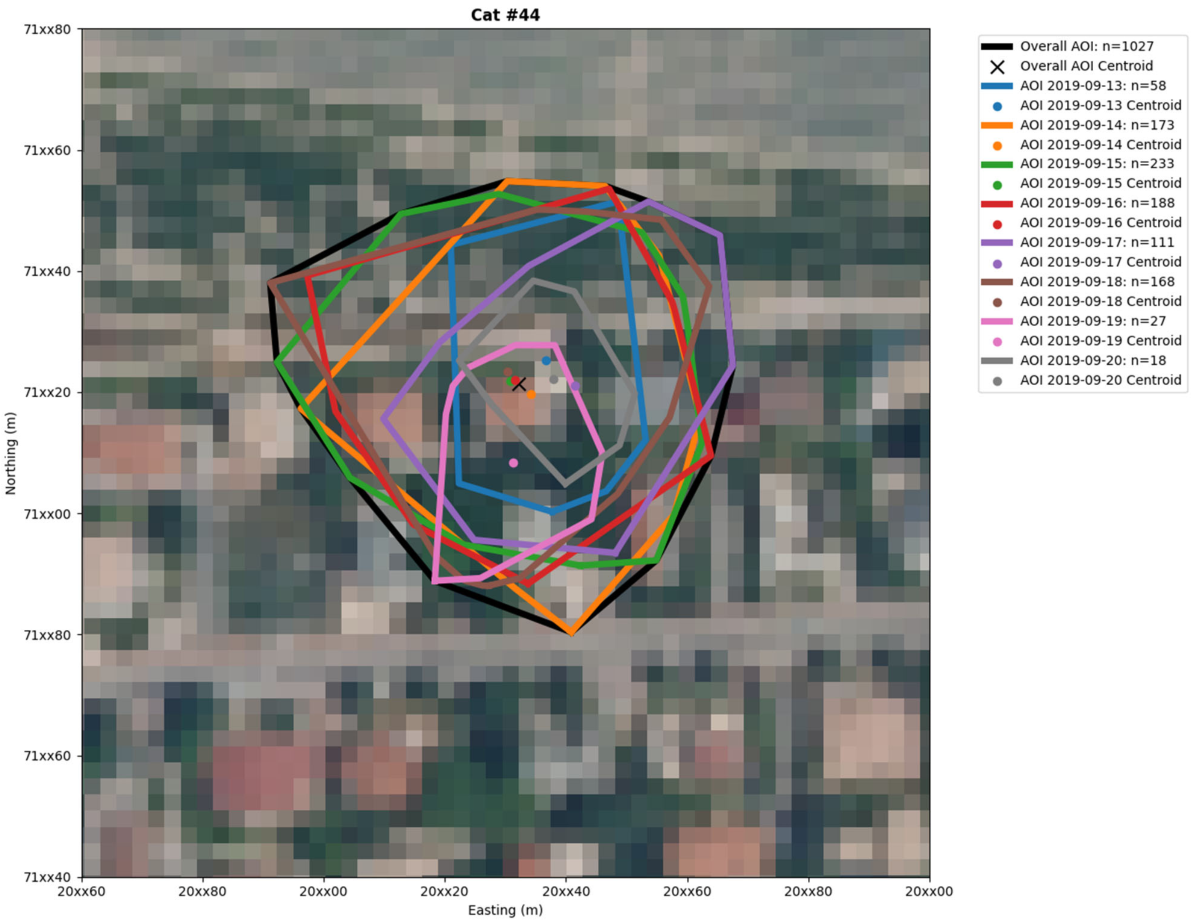

Cat #44
Northing (m)
Easting (m)
71xx80
71xx60
71xx40
71xx20
71xx00
71xx80
71xx60
71xx40
20xx60
20xx80
20xx00
20xx20
20xx40
20xx60
20xx80
20xx00
Overall AOI: n=1027
Overall AOI Centroid
AOI 2019-09-13: n=58
AOI 2019-09-13 Centroid
AOI 2019-09-14: n=173
AOI 2019-09-14 Centroid
AOI 2019-09-15: n=233
AOI 2019-09-15 Centroid
AOI 2019-09-16: n=188
AOI 2019-09-16 Centroid
AOI 2019-09-17: n=111
AOI 2019-09-17 Centroid
AOI 2019-09-18: n=168
AOI 2019-09-18 Centroid
AOI 2019-09-19: n=27
AOI 2019-09-19 Centroid
AOI 2019-09-20: n=18
AOI 2019-09-20 Centroid

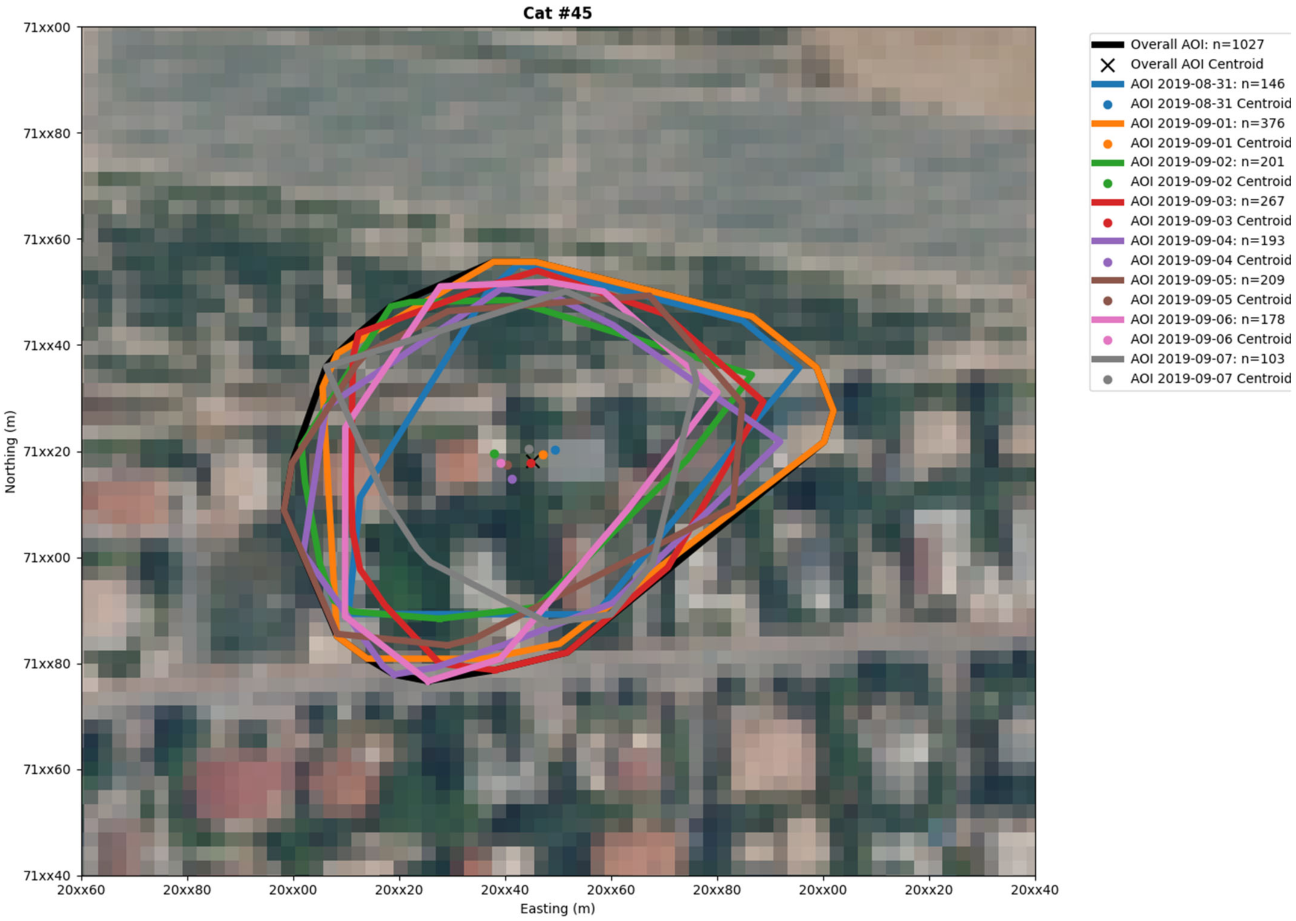

Cat #45
Northing (m)
Easting (m)
71xx00
71xx80
71xx60
71xx40
71xx20
71xx00
71xx80
71xx60
71xx40
20xx60
20xx80
20xx00
20xx20
20xx40
20xx60
20xx80
20xx00
20xx20
20xx40
Overall AOI: n=1027
Overall AOI Centroid
AOI 2019-08-31: n=146
AOI 2019-08-31 Centroid
AOI 2019-09-01: n=376
AOI 2019-09-01 Centroid
AOI 2019-09-02: n=201
AOI 2019-09-02 Centroid
AOI 2019-09-03: n=267
AOI 2019-09-03 Centroid
AOI 2019-09-04: n=193
AOI 2019-09-04 Centroid
AOI 2019-09-05: n=209
AOI 2019-09-05 Centroid
AOI 2019-09-06: n=178
AOI 2019-09-06 Centroid
AOI 2019-09-07: n=103
AOI 2019-09-07 Centroid

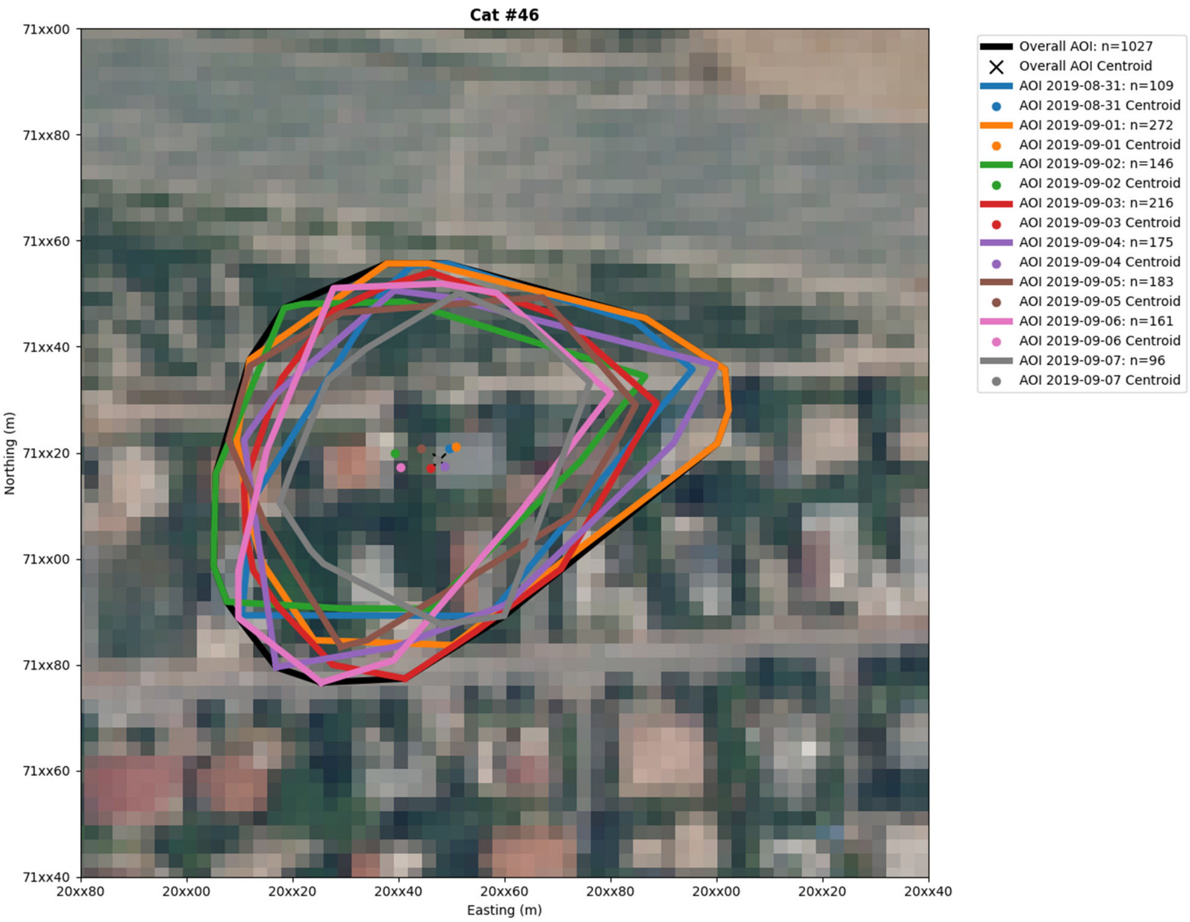
Cat #46
Overall AOI: n=1027
Overall AOI Centroid
AOI 2019-08-31: n=109
AOI 2019-08-31 Centroid
AOI 2019-09-01: n=272
AOI 2019-09-01 Centroid
AOI 2019-09-02: n=146
AOI 2019-09-02 Centroid
AOI 2019-09-03: n=216
AOI 2019-09-03 Centroid
AOI 2019-09-04: n=175
AOI 2019-09-04 Centroid
AOI 2019-09-05: n=183
AOI 2019-09-05 Centroid
AOI 2019-09-06: n=161
AOI 2019-09-06 Centroid
AOI 2019-09-07: n=96
AOI 2019-09-07 Centroid
Northing (m)
Easting (m)
71xx00
71xx80
71xx60
71xx40
71xx20
71xx00
71xx80
71xx60
71xx40
20xx80
20xx00
20xx20
20xx40
20xx60
20xx80
20xx00
20xx20
20xx40

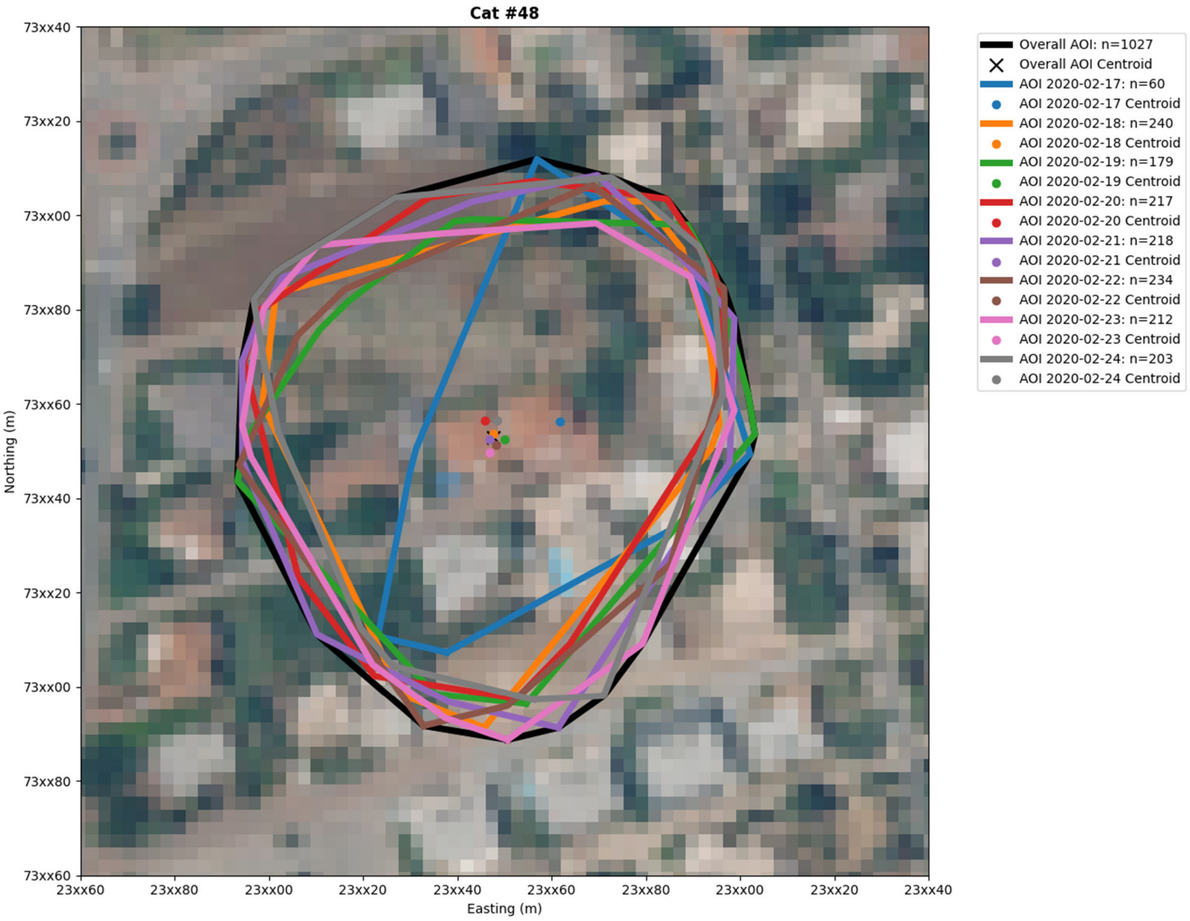
Cat #48
Overall AOI: n=1027
Overall AOI Centroid
AOI 2020-02-17: n=60
AOI 2020-02-17 Centroid
AOI 2020-02-18: n=240
AOI 2020-02-18 Centroid
AOI 2020-02-19: n=179
AOI 2020-02-19 Centroid
AOI 2020-02-20: n=217
AOI 2020-02-20 Centroid
AOI 2020-02-21: n=218
AOI 2020-02-21 Centroid
AOI 2020-02-22: n=234
AOI 2020-02-22 Centroid
AOI 2020-02-23: n=212
AOI 2020-02-23 Centroid
AOI 2020-02-24: n=203
AOI 2020-02-24 Centroid
Northing (m)
Easting (m)
73xx40
73xx20
73xx00
73xx80
73xx60
73xx40
73xx20
73xx00
73xx80
73xx60
23xx60
23xx80
23xx00
23xx20
23xx40
23xx60
23xx80
23xx00
23xx20
23xx40

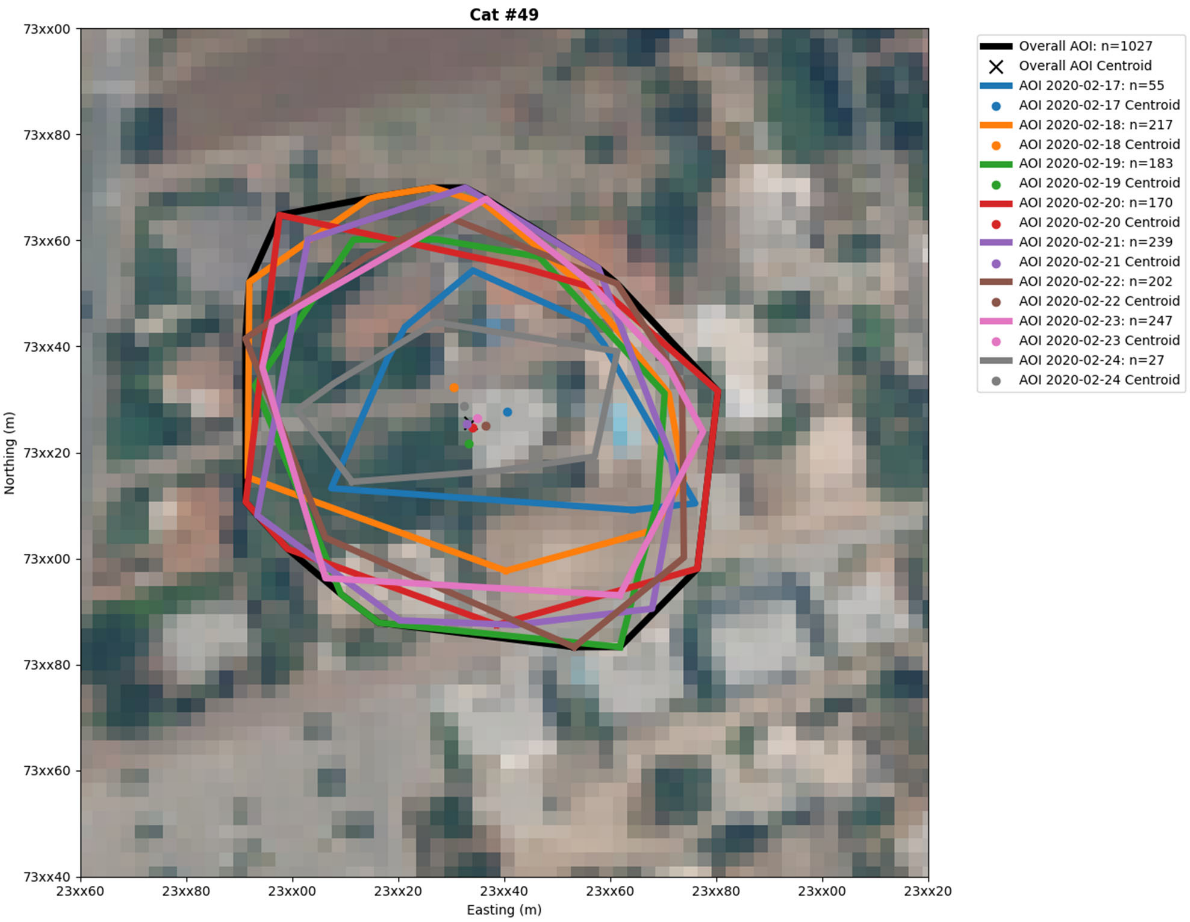
Cat #49
73xx00
73xx80
73xx60
73xx40
73xx20
73xx00
73xx80
73xx60
73xx40
Northing (m)
23xx60
23xx80
23xx00
23xx20
23xx40
23xx60
23xx80
23xx00
23xx20
Easting (m)
Overall AOI: n=1027
Overall AOI Centroid
AOI 2020-02-17: n=55
AOI 2020-02-17 Centroid
AOI 2020-02-18: n=217
AOI 2020-02-18 Centroid
AOI 2020-02-19: n=183
AOI 2020-02-19 Centroid
AOI 2020-02-20: n=170
AOI 2020-02-20 Centroid
AOI 2020-02-21: n=239
AOI 2020-02-21 Centroid
AOI 2020-02-22: n=202
AOI 2020-02-22 Centroid
AOI 2020-02-23: n=247
AOI 2020-02-23 Centroid
AOI 2020-02-24: n=27
AOI 2020-02-24 Centroid

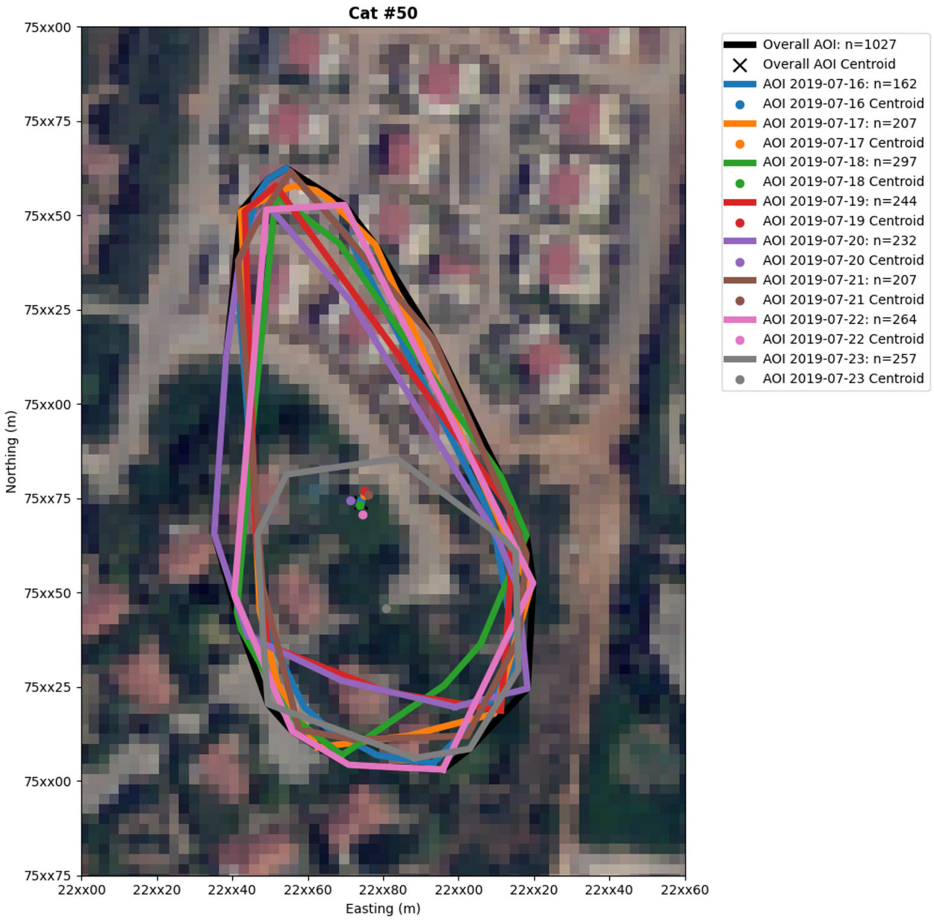
Cat #50
75xx00
75xx75
75xx50
75xx25
75xx00
75xx75
75xx50
75xx25
75xx00
75xx75
Northing (m)
22xx00
22xx20
22xx40
22xx60
22xx80
22xx00
22xx20
22xx40
22xx60
Easting (m)
Overall AOI: n=1027
Overall AOI Centroid
AOI 2019-07-16: n=162
AOI 2019-07-16 Centroid
AOI 2019-07-17: n=207
AOI 2019-07-17 Centroid
AOI 2019-07-18: n=297
AOI 2019-07-18 Centroid
AOI 2019-07-19: n=244
AOI 2019-07-19 Centroid
AOI 2019-07-20: n=232
AOI 2019-07-20 Centroid
AOI 2019-07-21: n=207
AOI 2019-07-21 Centroid
AOI 2019-07-22: n=264
AOI 2019-07-22 Centroid
AOI 2019-07-23: n=257
AOI 2019-07-23 Centroid

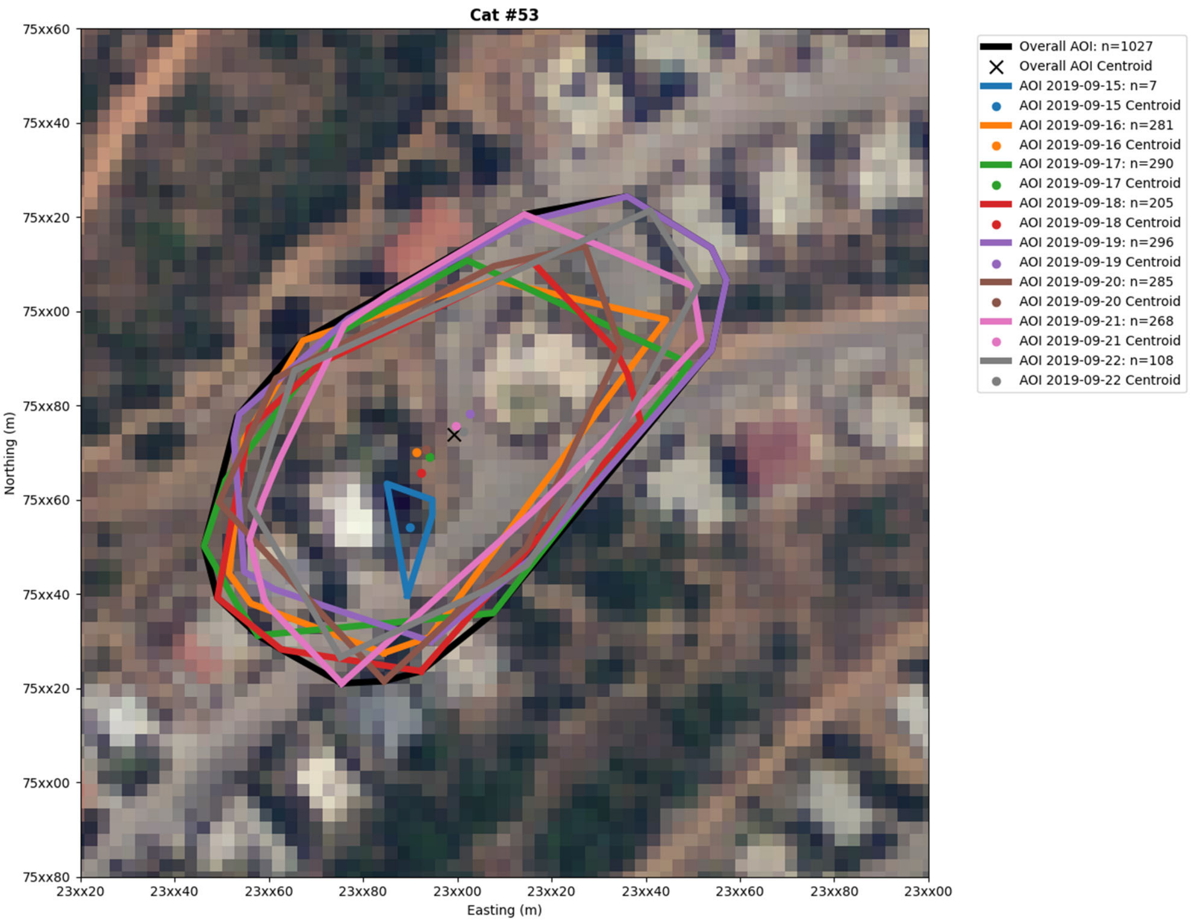
Cat #53
Overall AOI: n=1027
Overall AOI Centroid
AOI 2019-09-15: n=7
AOI 2019-09-15 Centroid
AOI 2019-09-16: n=281
AOI 2019-09-16 Centroid
AOI 2019-09-17: n=290
AOI 2019-09-17 Centroid
AOI 2019-09-18: n=205
AOI 2019-09-18 Centroid
AOI 2019-09-19: n=296
AOI 2019-09-19 Centroid
AOI 2019-09-20: n=285
AOI 2019-09-20 Centroid
AOI 2019-09-21: n=268
AOI 2019-09-21 Centroid
AOI 2019-09-22: n=108
AOI 2019-09-22 Centroid
Northing (m)
75xx60
75xx40
75xx20
75xx00
75xx80
75xx60
75xx40
75xx20
75xx00
75xx80
Easting (m)
23xx20
23xx40
23xx60
23xx80
23xx00
23xx20
23xx40
23xx60
23xx80
23xx00

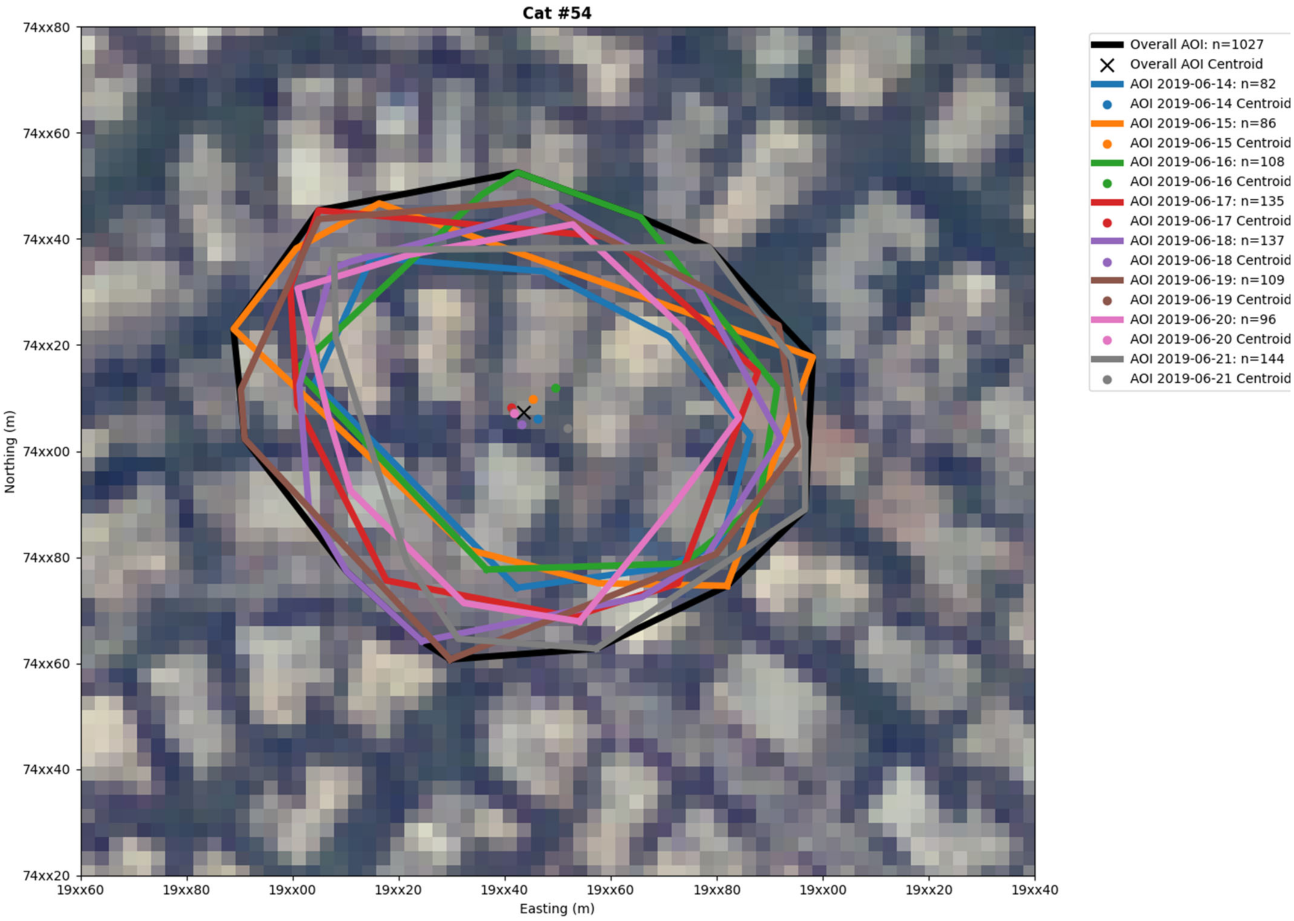
Cat #54
Overall AOI: n=1027
Overall AOI Centroid
AOI 2019-06-14: n=82
AOI 2019-06-14 Centroid
AOI 2019-06-15: n=86
AOI 2019-06-15 Centroid
AOI 2019-06-16: n=108
AOI 2019-06-16 Centroid
AOI 2019-06-17: n=135
AOI 2019-06-17 Centroid
AOI 2019-06-18: n=137
AOI 2019-06-18 Centroid
AOI 2019-06-19: n=109
AOI 2019-06-19 Centroid
AOI 2019-06-20: n=96
AOI 2019-06-20 Centroid
AOI 2019-06-21: n=144
AOI 2019-06-21 Centroid
Northing (m)
74xx80
74xx60
74xx40
74xx20
74xx00
74xx80
74xx60
74xx40
74xx20
Easting (m)
19xx60
19xx80
19xx00
19xx20
19xx40
19xx60
19xx80
19xx00
19xx20
19xx40

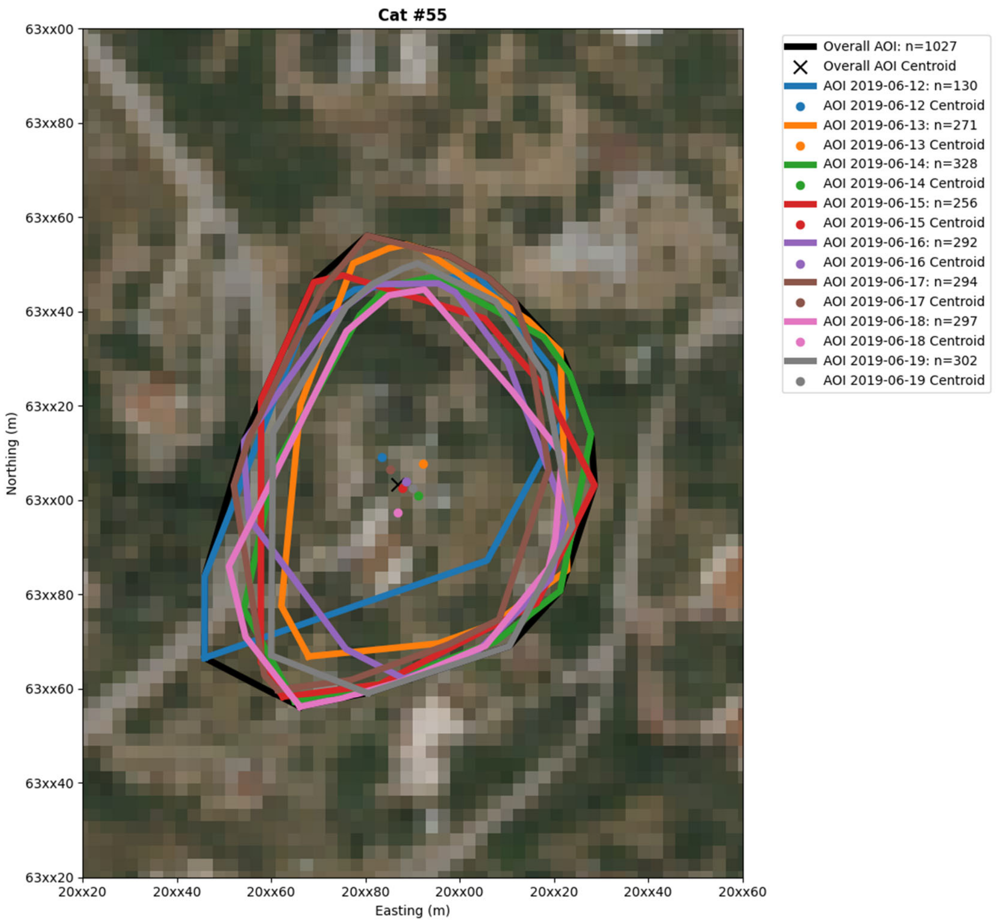
Cat #55
Overall AOI: n=1027
Overall AOI Centroid
AOI 2019-06-12: n=130
AOI 2019-06-12 Centroid
AOI 2019-06-13: n=271
AOI 2019-06-13 Centroid
AOI 2019-06-14: n=328
AOI 2019-06-14 Centroid
AOI 2019-06-15: n=256
AOI 2019-06-15 Centroid
AOI 2019-06-16: n=292
AOI 2019-06-16 Centroid
AOI 2019-06-17: n=294
AOI 2019-06-17 Centroid
AOI 2019-06-18: n=297
AOI 2019-06-18 Centroid
AOI 2019-06-19: n=302
AOI 2019-06-19 Centroid
Northing (m)
63xx00
63xx80
63xx60
63xx40
63xx20
63xx00
63xx80
63xx60
63xx40
63xx20
Easting (m)
20xx20
20xx40
20xx60
20xx80
20xx00
20xx20
20xx40
20xx60

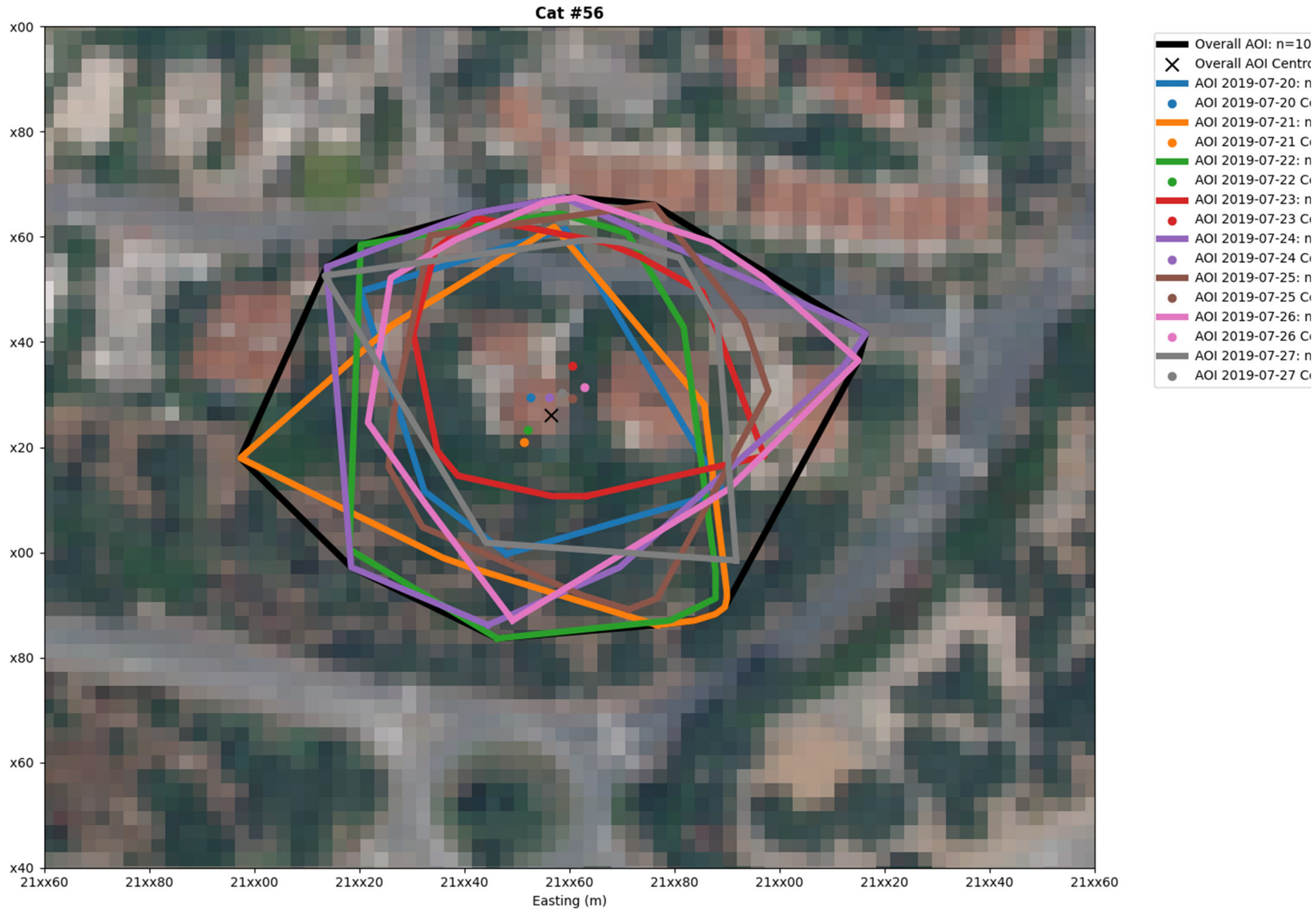
Cat #56
Overall AOI: n=10
Overall AOI Centro
AOI 2019-07-20: n
AOI 2019-07-20 C
AOI 2019-07-21: n
AOI 2019-07-21 C
AOI 2019-07-22: n
AOI 2019-07-22 C
AOI 2019-07-23: n
AOI 2019-07-23 C
AOI 2019-07-24: n
AOI 2019-07-24 C
AOI 2019-07-25: n
AOI 2019-07-25 C
AOI 2019-07-26: n
AOI 2019-07-26 C
AOI 2019-07-27: n
AOI 2019-07-27 C
x00
x80
x60
x40
x20
x00
x80
x60
x40
Easting (m)
21xx60
21xx80
21xx00
21xx20
21xx40
21xx60
21xx80
21xx00
21xx20
21xx40
21xx60

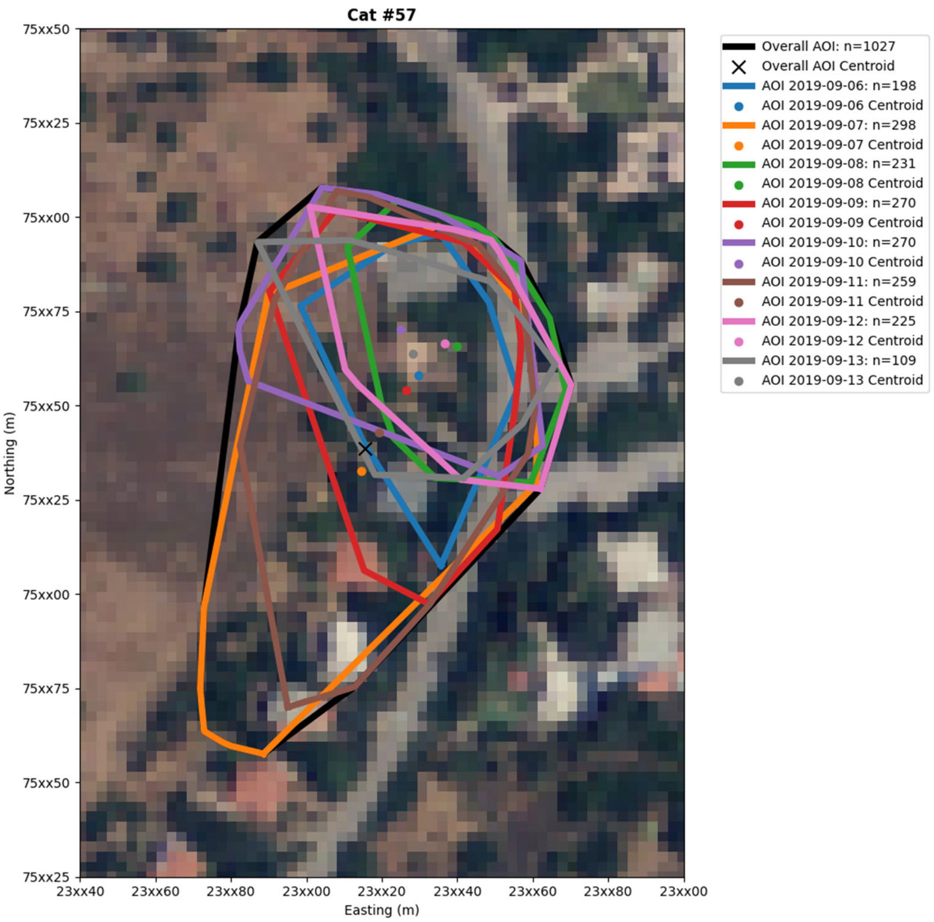
Cat #57
75xx50
75xx25
75xx00
75xx75
75xx50
75xx25
75xx00
75xx75
75xx50
75xx25
Northing (m)
23xx40
23xx60
23xx80
23xx00
23xx20
23xx40
23xx60
23xx80
23xx00
Easting (m)
Overall AOI: n=1027
Overall AOI Centroid
AOI 2019-09-06: n=198
AOI 2019-09-06 Centroid
AOI 2019-09-07: n=298
AOI 2019-09-07 Centroid
AOI 2019-09-08: n=231
AOI 2019-09-08 Centroid
AOI 2019-09-09: n=270
AOI 2019-09-09 Centroid
AOI 2019-09-10: n=270
AOI 2019-09-10 Centroid
AOI 2019-09-11: n=259
AOI 2019-09-11 Centroid
AOI 2019-09-12: n=225
AOI 2019-09-12 Centroid
AOI 2019-09-13: n=109
AOI 2019-09-13 Centroid

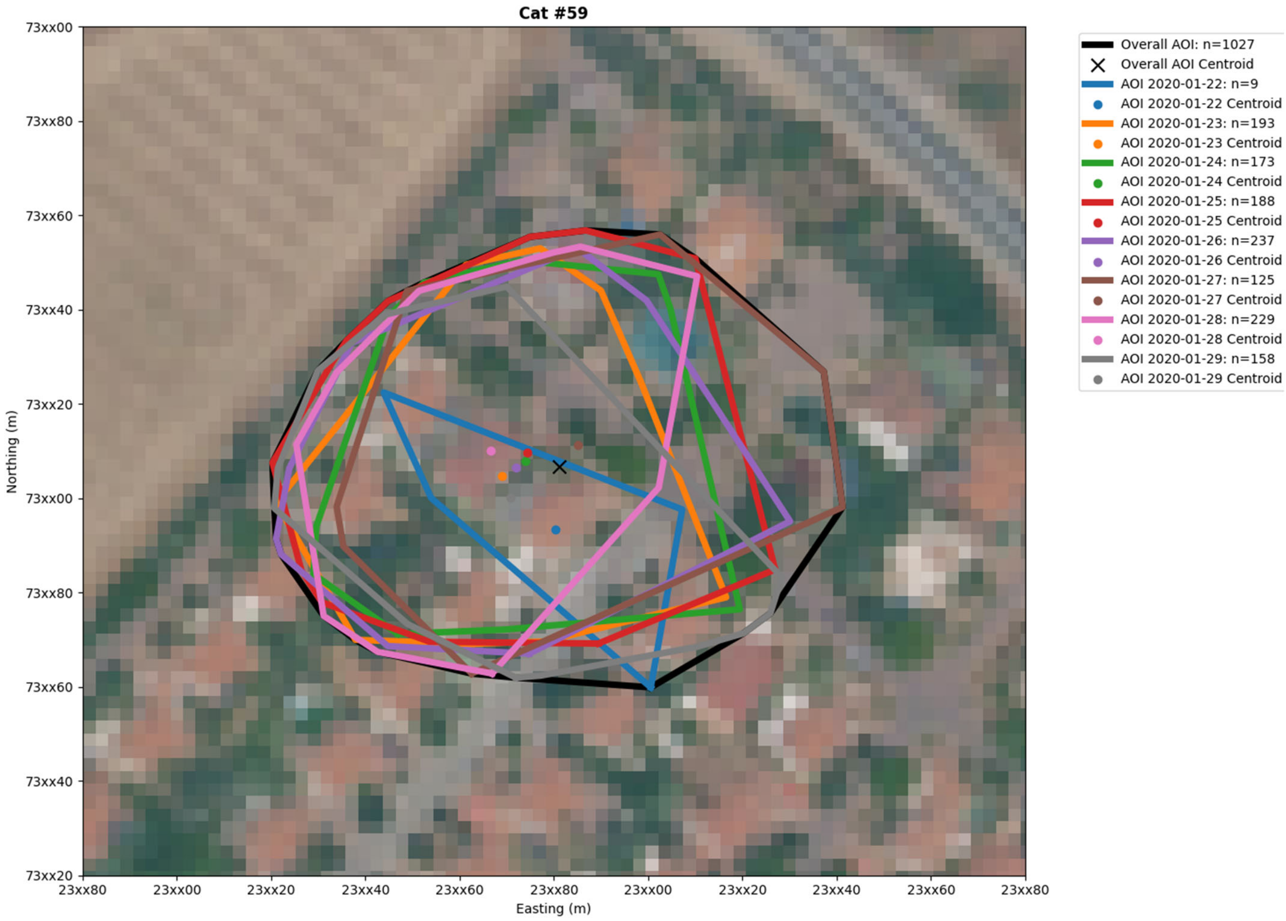
Cat #59
73xx00
73xx80
73xx60
73xx40
73xx20
73xx00
73xx80
73xx60
73xx40
73xx20
Northing (m)
23xx80
23xx00
23xx20
23xx40
23xx60
23xx80
23xx00
23xx20
23xx40
23xx60
23xx80
Easting (m)
Overall AOI: n=1027
Overall AOI Centroid
AOI 2020-01-22: n=9
AOI 2020-01-22 Centroid
AOI 2020-01-23: n=193
AOI 2020-01-23 Centroid
AOI 2020-01-24: n=173
AOI 2020-01-24 Centroid
AOI 2020-01-25: n=188
AOI 2020-01-25 Centroid
AOI 2020-01-26: n=237
AOI 2020-01-26 Centroid
AOI 2020-01-27: n=125
AOI 2020-01-27 Centroid
AOI 2020-01-28: n=229
AOI 2020-01-28 Centroid
AOI 2020-01-29: n=158
AOI 2020-01-29 Centroid

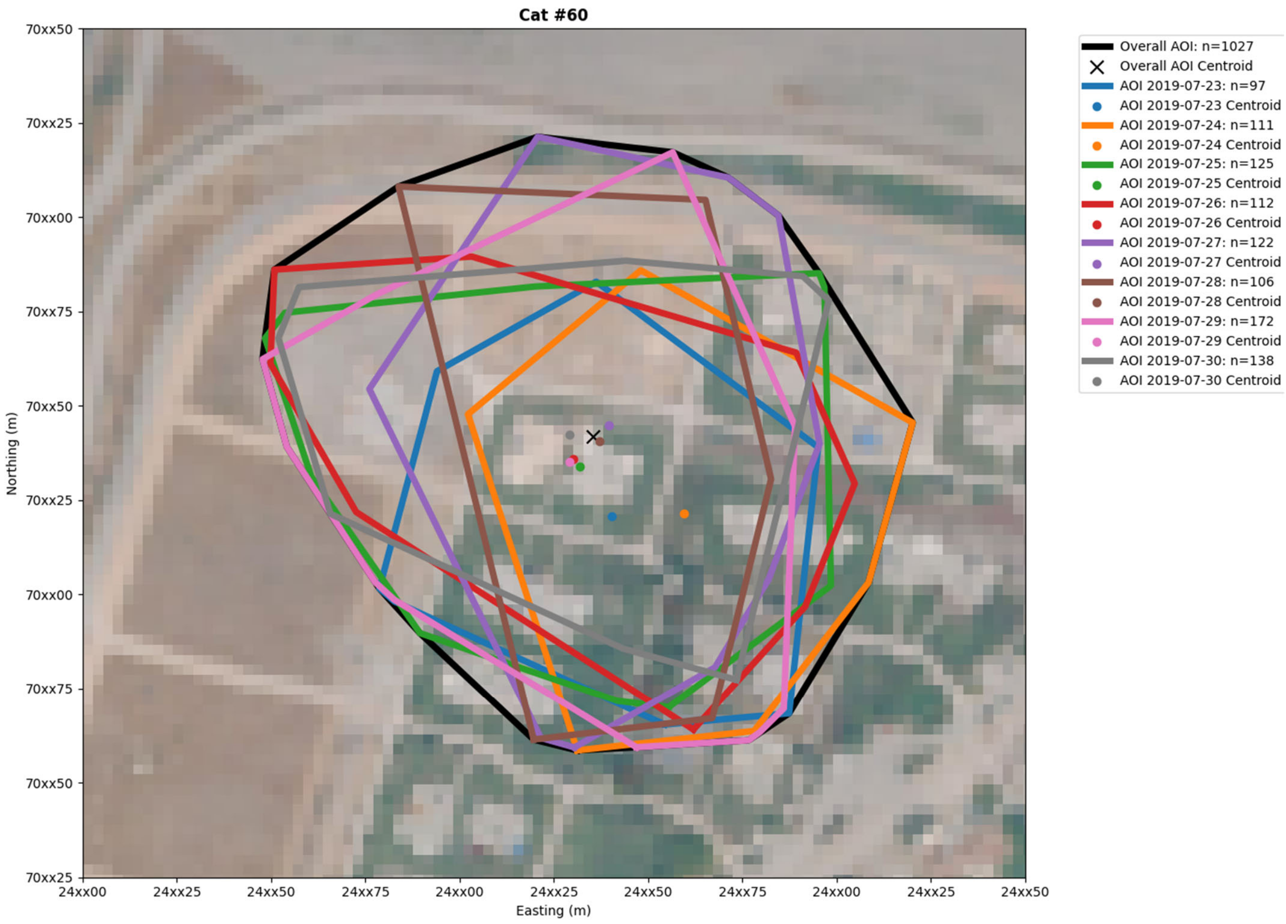
Cat #60
70xx50
70xx25
70xx00
70xx75
70xx50
70xx25
70xx00
70xx75
70xx50
70xx25
Northing (m)
24xx00
24xx25
24xx50
24xx75
24xx00
24xx25
24xx50
24xx75
24xx00
24xx25
24xx50
Easting (m)
Overall AOI: n=1027
Overall AOI Centroid
AOI 2019-07-23: n=97
AOI 2019-07-23 Centroid
AOI 2019-07-24: n=111
AOI 2019-07-24 Centroid
AOI 2019-07-25: n=125
AOI 2019-07-25 Centroid
AOI 2019-07-26: n=112
AOI 2019-07-26 Centroid
AOI 2019-07-27: n=122
AOI 2019-07-27 Centroid
AOI 2019-07-28: n=106
AOI 2019-07-28 Centroid
AOI 2019-07-29: n=172
AOI 2019-07-29 Centroid
AOI 2019-07-30: n=138
AOI 2019-07-30 Centroid

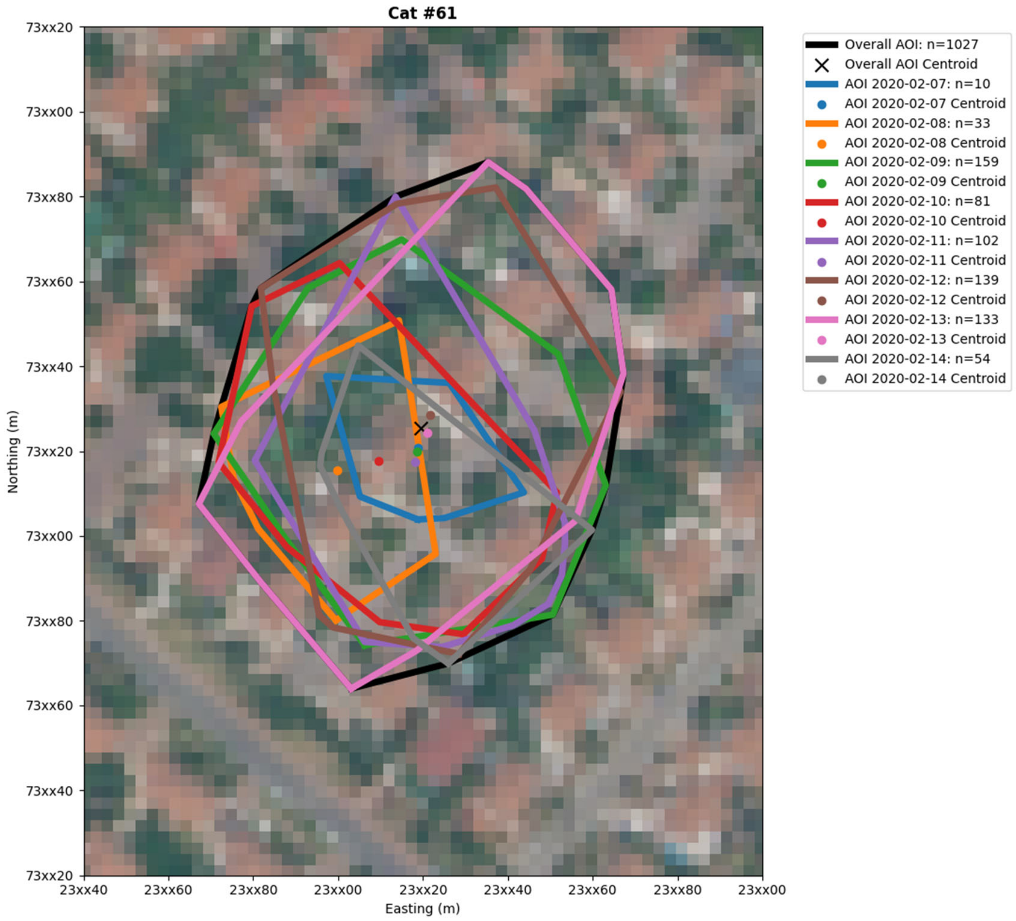
Cat #61
73xx20
73xx00
73xx80
73xx60
73xx40
73xx20
73xx00
73xx80
73xx60
73xx40
73xx20
Northing (m)
23xx40
23xx60
23xx80
23xx00
23xx20
23xx40
23xx60
23xx80
23xx00
Easting (m)
Overall AOI: n=1027
Overall AOI Centroid
AOI 2020-02-07: n=10
AOI 2020-02-07 Centroid
AOI 2020-02-08: n=33
AOI 2020-02-08 Centroid
AOI 2020-02-09: n=159
AOI 2020-02-09 Centroid
AOI 2020-02-10: n=81
AOI 2020-02-10 Centroid
AOI 2020-02-11: n=102
AOI 2020-02-11 Centroid
AOI 2020-02-12: n=139
AOI 2020-02-12 Centroid
AOI 2020-02-13: n=133
AOI 2020-02-13 Centroid
AOI 2020-02-14: n=54
AOI 2020-02-14 Centroid
